# Tpc1 is an important Zn(II)_2_Cys_6_ transcriptional regulator required for polarized growth and virulence in the rice blast fungus

**DOI:** 10.1371/journal.ppat.1006516

**Published:** 2017-07-24

**Authors:** Rita Galhano, Adriana Illana, Lauren S. Ryder, Julio Rodríguez-Romero, Marie Demuez, Muhammad Badaruddin, Ana Lilia Martinez-Rocha, Darren M. Soanes, David J. Studholme, Nicholas J. Talbot, Ane Sesma

**Affiliations:** 1 Disease & Stress Biology Dept. John Innes Centre, Norwich, United Kingdom; 2 School of Biosciences, University of Exeter, Geoffrey Pope Building, Exeter, United Kingdom; 3 Centre for Plant Biotechnology and Genomics (CBGP), Universidad Politécnica de Madrid, (UPM) – Instituto Nacional de Investigación y Tecnología Agraria y Alimentaria (INIA), Pozuelo de Alarcón, Madrid, Spain; 4 Dept. Biotecnología y Biología Vegetal, UPM, Madrid, Spain; Scottish Crop Research Institute, UNITED KINGDOM

## Abstract

The establishment of polarity is a critical process in pathogenic fungi, mediating infection-related morphogenesis and host tissue invasion. Here, we report the identification of *TPC1* (Transcription factor for Polarity Control 1), which regulates invasive polarized growth in the rice blast fungus *Magnaporthe oryzae*. *TPC1* encodes a putative transcription factor of the fungal Zn(II)_2_Cys_6_ family, exclusive to filamentous fungi. Tpc1-deficient mutants show severe defects in conidiogenesis, infection-associated autophagy, glycogen and lipid metabolism, and plant tissue colonisation. By tracking actin-binding proteins, septin-5 and autophagosome components, we show that Tpc1 regulates cytoskeletal dynamics and infection-associated autophagy during appressorium-mediated plant penetration. We found that Tpc1 interacts with Mst12 and modulates its DNA-binding activity, while Tpc1 nuclear localisation also depends on the MAP kinase Pmk1, consistent with the involvement of Tpc1 in this signalling pathway, which is critical for appressorium development. Importantly, Tpc1 directly regulates *NOXD* expression, the p22^phox^ subunit of the fungal NADPH oxidase complex via an interaction with Mst12. Tpc1 therefore controls spatial and temporal regulation of cortical F-actin through regulation of the NADPH oxidase complex during appressorium re-polarisation. Consequently, Tpc1 is a core developmental regulator in filamentous fungi, linking the regulated synthesis of reactive oxygen species and the Pmk1 pathway, with polarity control during host invasion.

## Author summary

Cellular polarity is an intrinsic feature of filamentous fungal growth and pathogenesis. In this study, we identified a gene required for fungal polar growth and virulence in the rice blast fungus *Magnaporthe oryzae*. This gene has been named *TPC1* (Transcription factor for Polarity Control 1). The Tpc1 protein belongs to the fungal Zn(II)_2_Cys_6_ binuclear cluster family. This DNA-binding motif is present exclusively in the fungal kingdom. We have characterised defects associated with lack of Tpc1 in *M*. *oryzae*. We show that Tpc1 is involved in polarised growth and virulence. The *M*. *oryzae* Δ*tpc1* mutant shows a delay in glycogen and lipid metabolism, and infection-associated autophagy–processes that regulate appressorium-mediated *M*. *oryzae* plant infection. The saprophytic fungus *Neurospora crassa* contains a Tpc1 homolog (NcTpc1) involved in vegetative growth and sustained tip elongation, suggesting that Tpc1-like proteins act as core regulators of polarised growth and development in filamentous fungi. A comparative transcriptome analysis has allowed us to identify genes regulated by Tpc1 in *M*. *oryzae* including NoxD, an important component of the fungal NADPH complex. Significantly, Tpc1 interacts with Mst12, a component of the Pmk1 signalling pathway essential for appressorium development, and modulates Mst12 binding affinity to *NOXD* promoter region. We conclude that Tpc1 is a key regulator of polarity in *M*. *oryzae* that regulates growth, autophagy and septin-mediated reorientation of the F-actin cytoskeleton to facilitate plant cell invasion.

## Introduction

Rice blast disease is one of the most serious diseases of cultivated rice worldwide and is caused by the filamentous, ascomycete fungus *Magnaporthe oryzae*[1,2]. The disease is initiated when a conidium lands on the rice leaf surface. Here it germinates to produce a single germ tube that differentiates at its tip to develop a specialised infection structure called an appressorium[3]. During the initial stages of appressorium formation, a septum defines the developing appressorium from the rest of the germ tube following a single mitotic division in the germ tube[4]. When the appressorium matures, the three conidial cells and germ tube collapse due to infection-associated autophagy and are no longer viable after 24h[4]. Subsequently, a penetration peg emerges from the base of the appressorium and ruptures the leaf cuticle. A toroidal filamentous actin network forms at the base of the appressorium pore, scaffolded by septin GTPases[5]. Assembly of the four core septin GTPases is regulated by the Nox2 NADPH oxidase complex, which is required for re-modelling of the F-actin cytoskeleton and assembling the exocyst at the appressorium pore [6,7,8]. F-actin ring formation is necessary for penetration peg emergence and re-establishment of polarized growth at the point of plant penetration. After penetration, the fungal peg grows as a narrow, short primary invasive hypha[9], before differentiating into bulbous invasive hyphae during colonisation of the first invaded host cell[10]. Disease symptoms appear between 72h and 96h after initial infection and coalesce into large spreading necrotic lesions from which the fungus sporulates. *M*. *oryzae* has also the capacity to penetrate roots by means of hyphopodia and can colonize root tissue and spread systemically throughout the plant under laboratory conditions [11,12].

In this study, we report the identification of a novel Zn(II)_2_Cys_6_ transcriptional regulator involved in the early stages of plant infection by *M*. *oryzae*. The Zn(II)_2_Cys_6_ binuclear cluster domain (IPR001138, PF00172) is exclusively found in the fungal kingdom[13,14]. The six cysteine residues bind two zinc atoms, which coordinate folding of the domain involved in DNA-binding. Most Zn(II)_2_Cys_6_ proteins have been studied in *Saccharomyces cerevisiae* and *Aspergillus* species[13,15,16]. Typically, the Zn(II)_2_Cys_6_ proteins are pathway-specific activators under the control of major regulators[15,16,17,18]. The regulator of galactose catabolism in yeast, Gal4p[19], and the regulators of acetate assimilation FacB[20] and the aflatoxin cluster AflR[21] in *A*. *nidulans*, are among the best studied examples. Several Zn(II)_2_Cys_6_ transcriptional regulators have been studied in the rice blast fungus (S1 Table). Of the 175 members of the Zn(II)_2_Cys_6_ binuclear cluster family present in *M*. *oryzae* (S2 Table), only nine of them (MoCod1, MoCod2, Pig1, Tra1, Tdg3, Xlr1, Ara1, Far1 and Far2) have been examined in any detail[22,23,24,25,26,27] (S1 Table). A high-throughput gene knockout approach of 104 Zn(II)_2_Cys_6_ proteins in *M*. *oryzae* revealed large variation in their biological functions, and reported seven additional Zn(II)_2_Cys_6_ proteins to be required for plant infection, including Gpf1 and Cln2[28]. However, despite this information, the mechanistic insights into how the Zn(II)_2_Cys_6_ proteins govern *M*. *oryzae* cellular processes are largely unknown.

In this study, we characterize a novel mutant of *M*. *oryzae* that shows defects in pathogenicity and vegetative growth following its selection from a *M*. *oryzae* T-DNA insertional library. The T-DNA insertion is located within a gene (MGG_01285) encoding a Zn(II)_2_Cys_6_ binuclear cluster protein, which we name *TPC1*. This gene was not included in the large-scale gene knockout analysis of 104 Zn(II)_2_Cys_6_ proteins [28], although a global gene expression analysis of transcription factors revealed that *TPC1* is overexpressed during development (conidiation, germination and appressorium formation), oxidative stress (methyl viologen treatment) and carbon starvation [29]. Here, we reveal the involvement of this transcriptional regulator in polarized growth, cell patterning and virulence in *M*. *oryzae*. Among the genes regulated by Tpc1 we found *NOXD*, an important component of the fungal NADPH complex. Significantly, Tpc1 interacts with Mst12 and mis-localises in the Δ*pmk1* background, linking Tpc1 to this pathogenicity-associated MAPK signalling pathway. We provide mechanistic insight into the role of Tpc1, a key regulator of polarity in *M*. *oryzae* that controls growth, autophagy and septin-mediated reorientation of the F-actin cytoskeleton to facilitate plant colonisation.

## Results

### *M*. *oryzae TPC1* mutants show defects in development and pathogenicity

**Fig 1:**
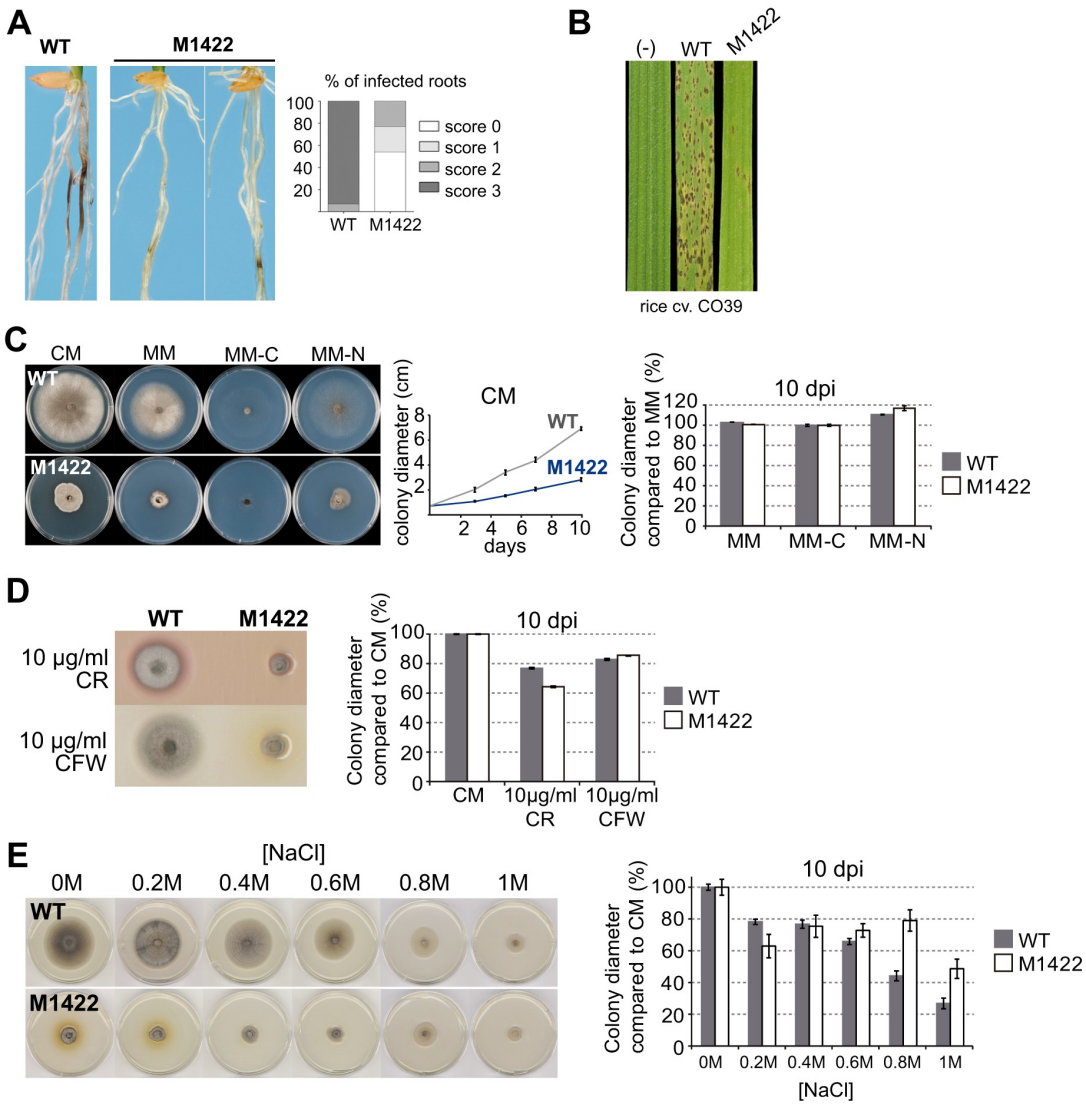
M1422 mutant shows severe defects in pathogenicity and vegetative growth together with increase tolerance to nitrogen depletion, calcofluor white (CFW) and hyperosmotic stress. **(A)** M1422 shows reduced disease symptoms on rice roots. Photographs were taken 15 days after inoculation. Wild-type (WT) strain Guy11 presents maximum lesion severity (score 3) in 95% of the infected roots compared to the M1422-infected seedlings that show mild (score 1–2) or null (score 0) necrotic symptoms on roots. Lesions were scored on a scale 0–3, based on colour intensity and extension of the necrotic lesion[30]. **(B)** M1422 is strongly impaired in its ability to infect rice leaves. **(C)** M1422 displays impaired ability to grow on complete medium (CM) and minimal medium (MM) depleted of carbon (MM-C) and nitrogen (MM-N). Relative to strain growth on MM, WT is more affected than M1422 on MM-N. **(D)** Sustained growth ratio difference in M1422 and WT strains on CM with 10 μg ml^-1^ Congo Red (CR) and 10 μg ml^-1^ CFW. Relative to growth on CM, M1422 shows stronger growth defects on CR compared to WT. **(E)** Colony images of CM plates with increased concentrations of sodium chloride (NaCl) (left). Diameter of WT and M1422 colonies measured at 10 dpi reveals increased tolerance to NaCl (right graph). All error bars represent the standard deviation of at least three independent biological replicas. All colony images were captured at 10 days post inoculation (dpi).

In order to identify novel infection-related genes we screened a total of 300 T-DNA transformants for their ability to infect rice roots using a *M*. *oryzae* insertional library[30]. The M1422 mutant developed very restricted disease lesions on roots and was selected for further characterization (Fig 1A). On leaves, M1422 produced only a small number of resistant-type lesions (Fig 1B and S1A Fig). Colonies of M1422 were also compact and reduced in size, when compared with the wild-type (Fig 1C).

The insertion site of the T-DNA within M1422 was located 0.9 kb downstream of the start codon of locus MGG_01285 in the *M*. *oryzae* genome (S1B Fig). This gene encodes a putative transcription factor that belongs to a Zn(II)_2_Cys_6_ binuclear cluster family. We named this gene *T**ranscription factor for* *P**olarity* *C**ontrol* *1* (*TPC1*). The predicted coding region of *TPC1* is ~2.6 kb long and encodes 839 amino acids (http://fungi.ensembl.org/index.html; MG8). The *TPC1* predicted amino acid sequence includes a putative nuclear localisation signal (NLS) and one Zn(II)_2_Cys_6_ binuclear cluster DNA binding domain (S1B Fig).

A single T-DNA insertion in M1422 genome was detected by Southern blot hybridisation using the hygromycin phosphotransferase gene as a probe (S1C Fig). We also generated a second mutant in *TPC1* by targeted gene replacement (S2A and S2B Fig). We complemented both M1422 and Δ*tpc1* with a C-terminal *TPC1*:*GFP* gene fusion under control of its native promoter. The complemented mutants recovered normal mycelial growth, colonial morphology and full virulence on rice (S1D, S1E and S2C Figs). We conclude that mutants M1422 and Δ*tpc1* are impaired in *TPC1* function.

Two striking characteristics of M1422 and Δ*tpc1* were their impaired hyphal growth and colony morphology (Fig 1C, S1E and S2C Figs). Vegetative growth of Tpc1-lacking strains was severely compromised in both complete (CM) and minimal (MM) medium (p < 0.01), and showed compact colonies and non-invasive colony morphology (S1E, S1F and S2D Figs). In *Neurospora crassa*, a class of mutants with polarity defects also exhibited this type of colony morphology[31].

To analyse integrity of the cell wall, development of M1422 was evaluated in the presence of the anionic dyes, Congo Red (CR) and Calcofluor White (CFW), which interfere with fungal cell wall assembly by binding to β-1,4-glucan and chitin, respectively [32]. Additive growth defects were observed on M1422 development in the presence of CR but not in CFW (Fig 1D). In addition, mycelial growth was affected by NaCl-induced hyperosmotic stress (Fig 1E). High concentrations of NaCl (0.6M - 1.0M) changed the growth ratio in colonial size between wild-type and M1422, leading to an increase in the relative growth rate of M1422 compared to wild-type. Therefore, the lack of Tpc1 affected plant virulence, vegetative growth, colony morphology and hyperosmotic stress adaptation.

### *M*. *oryzae TPC1* is required for conidiogenesis and appressorium development

**Fig 2:**
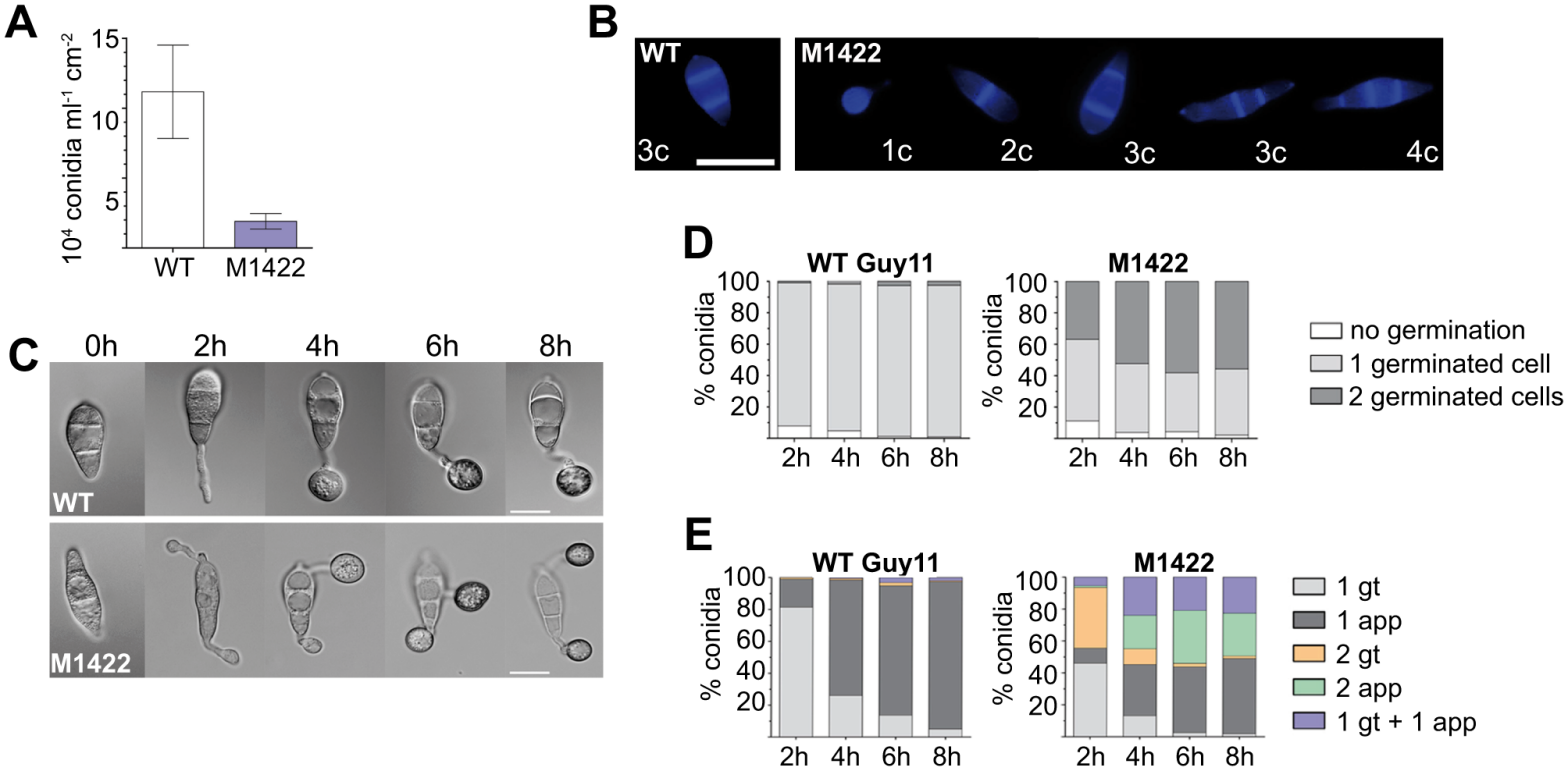
*M*. *oryzae TPC1* is required for conidiogenesis and infection-related development. **(A)** M1422 mycelia produces less conidia per cm^2^ than mycelia of wild-type (WT) strain on CM (mean±SD, n>300, three independent experiments). **(B)** Defects in the number of cells *per* conidium and conidia morphology of M1422. The M1422 mutant produces 1-, 2-, 3- (normal and abnormal morphology) and 4-celled conidia (respectively 1c, 2c, 3c and 4c in the panel) compared to the 3-celled conidia uniformly produced by the WT. Scale bar = 20 μm. **(C)** M1422 conidia inoculated onto glass coverslips showed an increased frequency of conidia germination from two cells. Scale bar = 10μm. **(D)** Quantification of M1422 defects in germination rate and development of germ tubes per conidia on coverslips. Two germ tubes germinated frequently from M1422 conidia. **(E)** Quantification of M1422 defects in the formation of infection-related structures; germ tubes (gt) and appressoria (app). M1422 conidia form at least one appressorium during infection-related development on coverslips (mean from >300 conidia; three biological repeats).

We observed that M1422 and Δ*tpc1* mutants sporulated poorly compared to wild-type (Fig 2A and S2E Fig). In addition, M1422 asexual spores showed defects in septation (numbers of cell per conidium) and conidial morphology (Fig 2B). Wild-type conidia were uniformly pyriform, three-celled spores. By contrast, in M1422, although the majority of conidia were three-celled (80%), a significant percentage of two-celled conidia (17%), single-celled (2%) and four-celled conidia (1%) were observed. Up to 26% of spores showed abnormal morphology in contrast to wild-type where less than 4% were misshapen (n> 300). We also found that appressorium development was affected in M1422 (Fig 2C). On hydrophobic coverslips, wild-type conidia germinated to form one germ tube that emerged from the apical cell and formed an appressorium within 4h-8h (Fig 2C and 2D). In M1422, 40% of conidia germinated from two cells. This percentage increased to 50%-60% with extended incubation time (4h-8h). Formation of two appressoria was rarely observed in wild-type conidia (Fig 2E). We conclude that M1422 is impaired in the normal spatial patterning of appressorium development.

### Polarity is coupled to autophagy and glycogen/lipid degradation in *M*. *oryzae*

**Fig 3:**
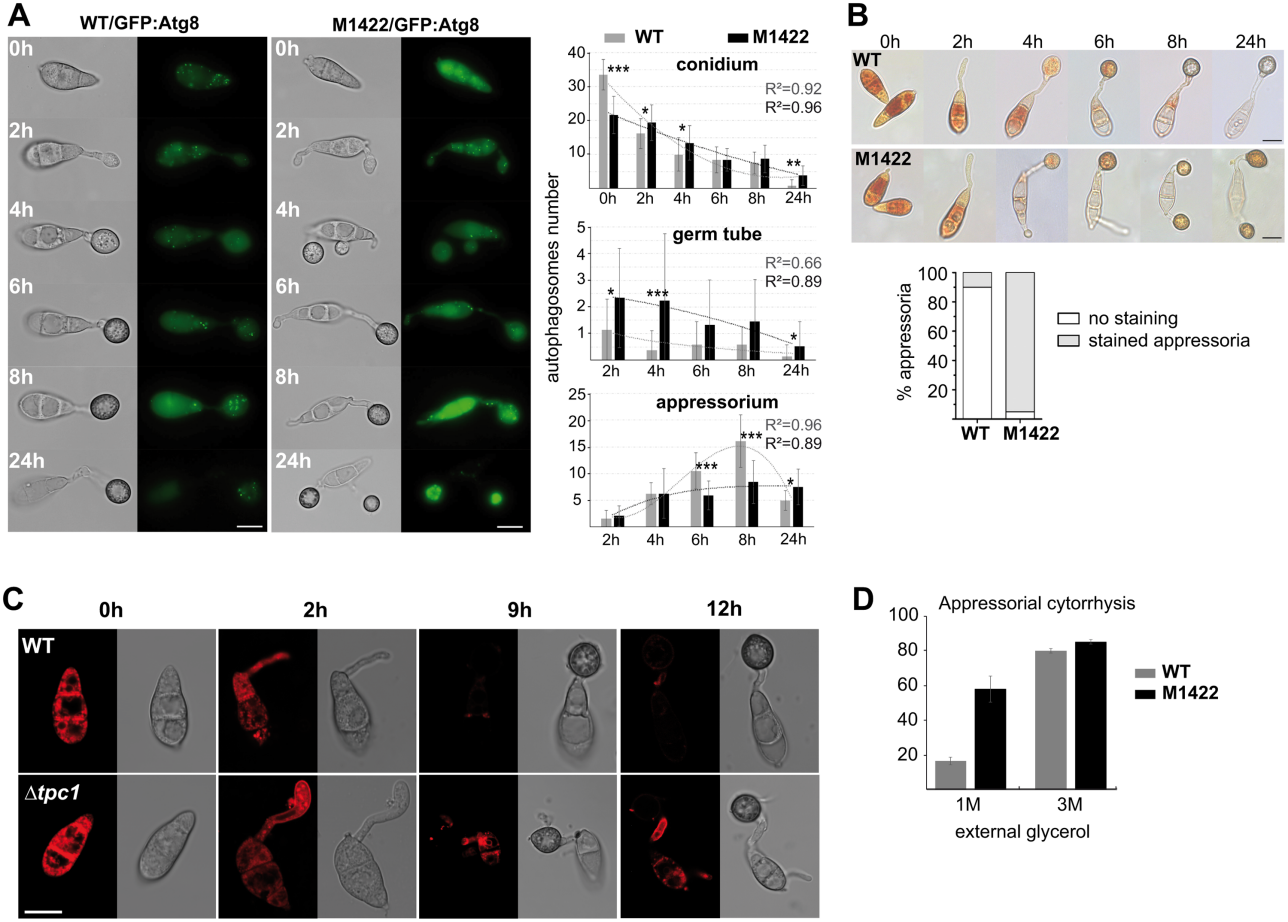
Infection-associated autophagy and glycogen/lipid degradation are impaired in Tpc1-lacking strains. **(A)** Impaired cellular localisation of autophagosomes in M1422 during infection-related development. Epifluorescence images of wild-type (WT) Guy11 and M1422 transformants expressing *GFP*:*MoATG8*. Scale bar = 10 μm. Bar charts show mean autophagosome numbers and trend lines present in conidium, germ tube, and appressorium at the time points indicated for each strain (mean ± SD; three biological repeats). Asterisks indicate significant differences of the medians with p values of <0.01 (*), <0.001(**) or <0.0001 (***) using the Mann-Whitney (Wilcoxon) W-test. **(B)** Glycogen metabolism is delayed in the M1422 mutant. Conidia from Guy11 and M1422 inoculated onto glass coverslips were exposed to potassium iodide (KI) solution at 0h, 2h, 4h, 6h, 8h and 24h after inoculation. The KI solution stains glycogen within the conidia, but not simple sugars such as glucose and fructose. Scale bar = 10 μm. Bar charts show the relative percentage of stained appressoria with KI solution for each strain at 24h after inoculation (three biological replicas). **(C)** Lipid droplet mobilization during appressorium maturation visualised using Nile red staining samples and confocal laser scanning microscopy. Fluorescence signals of all images were captured using the same parameters. Δ*tpc1*structures showing consistently higher fluorescence signals than the WT are evident at 9h and 12h. **(D)** In cytorrhysis assays with glycerol, Δ*tpc1* appressoria collapse quicker than WT appressoria, suggesting that glycerol concentration inside Δ*tpc1* appressoria is lower (mean±SD; three biological repeats; n>500).

The impairment of appressorium-mediated plant infection by *TPC1* mutants suggested that it might play a critical role in penetration peg development[33]. Appressorium function is known to depend on autophagic cell death of conidia, prior to appressorium maturation[4,34]. Therefore, we investigated whether infection-associated autophagy proceeded normally and if conidia underwent autophagic cell death. A *GFP*:*MoATG8* construct was introduced into M1422 to determine the spatial and temporal dynamics of autophagy (Fig 3A). *MoATG8* encodes an autophagic, ubiquitin-like protein involved in autophagosome function and has been shown to be a reliable marker for autophagy[4,34]. Compared to the wild-type Guy11 (33.5±4.4), *GFP*:*MoATG8*-labeled autophagosomes accumulated in M1422 conidia in significantly smaller numbers (21.6±5.5; p<0.01). In both strains, the number of conidial autophagosomes decreased during germination, appressorium maturation and at the onset of spore cell death and was significantly lower in M1422 conidia and germ tubes (Fig 3A). However, autophagosome numbers increased significantly during appressorium maturation (8h; 16.1±4.9) and dropped considerably after conidial death (24h; 5.0±1.8) in wild-type, whereas autophagosome number remained relatively constant in M1422 during appressorium maturation (8.4±4.1) and even after conidial cell death (7.5±3.3).

Appressorium development is accompanied by rapid degradation of glycogen from conidia during germination and from appressoria during turgor generation[35,36]. We therefore determined glycogen levels during appressorium development using potassium iodide (KI). Comparative analysis of KI staining between wild-type Guy11 and M1422 showed differences during the onset and later stages of conidial cell death (8h and 24h; Fig 3B). In Guy11 glycogen depletion was observed (no staining) within both conidial cells and appressoria during development. In the M1422 mutant, conidial cells were also depleted of glycogen although the appressorium still contained high levels of glycogen (95%) during maturation. We also looked at lipid metabolism, which is an additional driver of turgor generation in *M*. *oryzae*. The triacylglycerol lipase degrades lipid bodies that move to the appressorium during development[24,37]. Accordingly, we followed lipid body distribution during appressorium maturation in Δ*tpc1* using Nile red (Fig 3C). We consistently visualised delayed degradation of lipid bodies in conidia and germ tubes in Δ*tpc1*, which were evident at 9h and 12h after germination on coverslips. Using a cytorrhysis assay, in which hyperosmotic concentrations of a solute are applied to collapse appressoria, we estimated the internal solute concentration and turgor of appressoria of the two strains. We observed that Δ*tpc1* appressoria clearly collapsed at higher rates than the WT at 1M concentration of glycerol (Fig 3D). This suggests decreased in turgor within Δ*tpc1* appressoria, consistent with the observed delayed degradation of glycogen and lipid bodies. When considered together, these observations point that autophagy, and glycogen/lipid metabolisms are delayed during appressorium development in Tpc1-lacking strains.

### Tpc1 is required for re-establishment of polarity during appressorium-mediated plant infection

**Fig 4:**
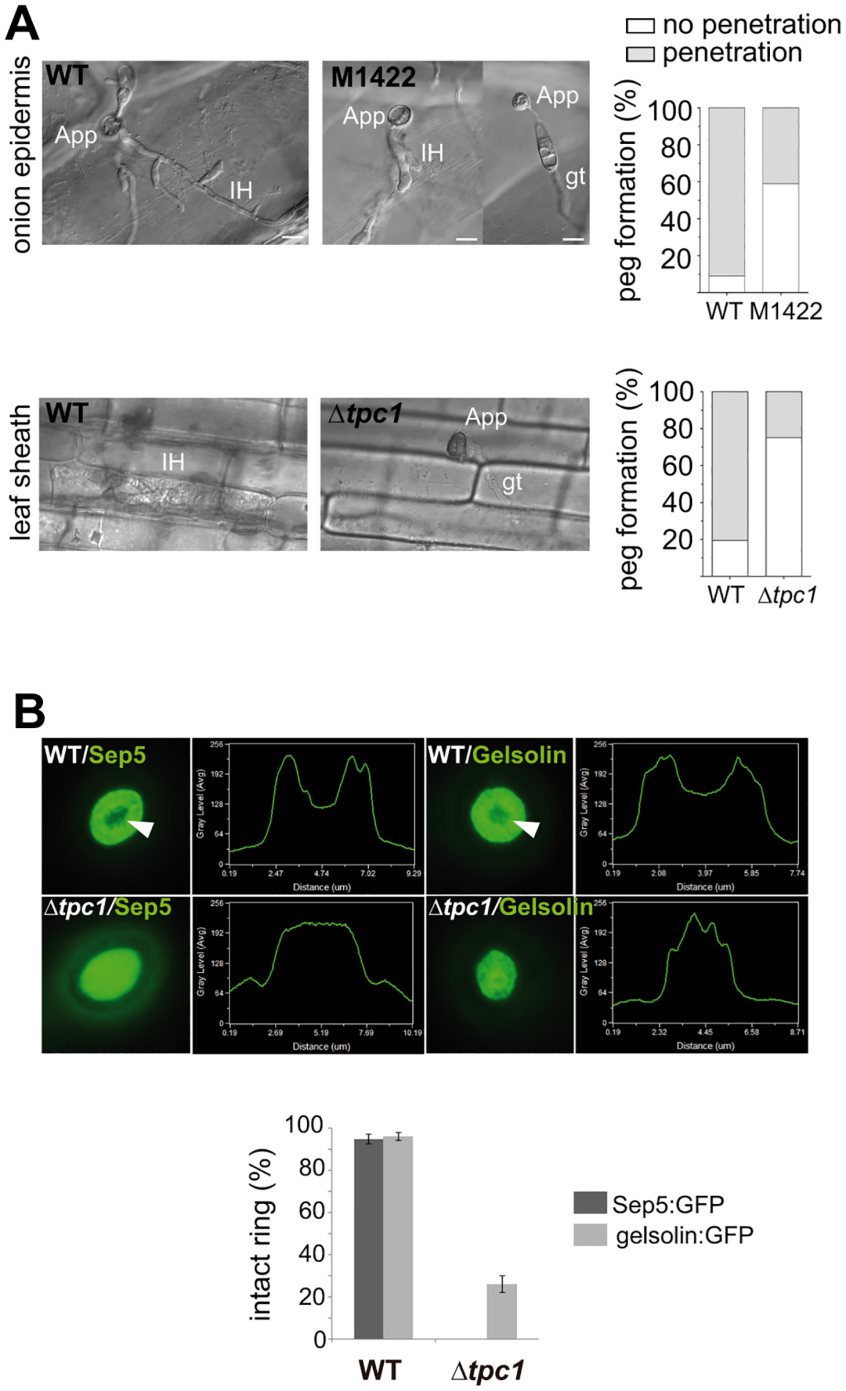
*M*. *oryzae* Tpc1 is required for re-establishment of polarity in appressoria. **(A)** Tpc1-lacking mutants are impaired in appressorium-mediated penetration. After 24 hpi wild-type (WT) appressoria formed on surface of the onion strip and leaf sheath have penetrated the underlying epidermal cell and formed invasive hyphae (IH). M1422 and Δ*tpc1* show a defective invasion of onion epidermis and leaf sheath, respectively. Scale bar = 10 μm. Bar charts show the relative percentage of appressorial-penetration of onion epidermis and leaf sheath for each strain at 24 hpi (three biological replicas). **(B)** Micrographs of F-actin ring organization visualized by expression of gelsolin:GFP and Sep:GFP in Guy11 and Δ*tpc1* strains. The Δ*tpc1* mutant produces aberrant septin and actin rings; mis-localization of Sep5:GFP is more severe. The linescan graphs show fluorescence in a transverse section of individual appressoria. White arrowheads point appressorial pores.

Following maturation of the appressorium, a penetration peg emerges from the appressorial pore to penetrate the plant cuticle and successfully colonise the plant host. To assess whether repolarization was impaired in the M1422 mutant, a penetration assay was performed on onion epidermis and rice leaf sheath (Fig 4A). After 24h, 91% of wild-type conidia formed an appressorium effectively, penetrated and invaded onion epidermal cells. The majority of M1422 conidia (60%) germinated and produced an appressorium, but failed to penetrate and invade onion cells. Only 40% of M1422 appressoria formed a penetration peg, but were not able to invade the onion epidermal cells and spread away from the point of penetration. Similarly, on rice leaf sheath preparations 81% of wild-type conidia penetrated successfully compared to 21% of Δ*tpc1* mutant spores, which managed to develop a penetration peg but hardly ever spread to adjacent cells.

To examine how formation of the germ tube and penetration peg was compromised, we investigated cellular organization of the F-actin cytoskeleton[38], using the actin-binding protein fimbrin tagged with GFP (S3 Fig). Once wild-type conidia attached to the surface, fimbrin:GFP spots were observed at the periphery of the germinating cells (0h). However, conidia harvested from M1422 instead localised F-actin randomly at the periphery of the three cells of conidia and not preferentially in the germinating cell (white arrowheads, S3 Fig). The most clear mis-localisation defects were observed in mutant appressoria. Fimbrin was localised in discrete puncta at the periphery of Guy11 appressoria, but in contrast was dispersed within appressoria of the mutant (6h). Furthermore, the F-actin network was more diffuse and several pores were observed in M1422 mature appressoria (white arrowheads, 24h). These results suggest that re-polarization of the appressorium is adversely affected in the M1422 mutant. To confirm this, we also tracked gelsolin:GFP and Sep5:GFP in Δ*tpc1* mutant. The use of gelsolin:GFP and Sep5:GFP to follow actin reorganization has helped to understand cytoskeleton dynamics during infection-related development[6]. The disorganisation of the appressorial cytoskeleton and actin ring was evident in Δ*tpc1*. Sep5 was mis-localised in all mutant appressoria and only 26% of mutant appressoria formed an intact actin ring with a central pore (Fig 4B). Consequently, *TPC1* is required for the correct penetration peg emergence in *M*. *oryzae*.

### Tpc1 is a nuclear protein regulated by the Pmk1 signalling cascade

**Fig 5:**
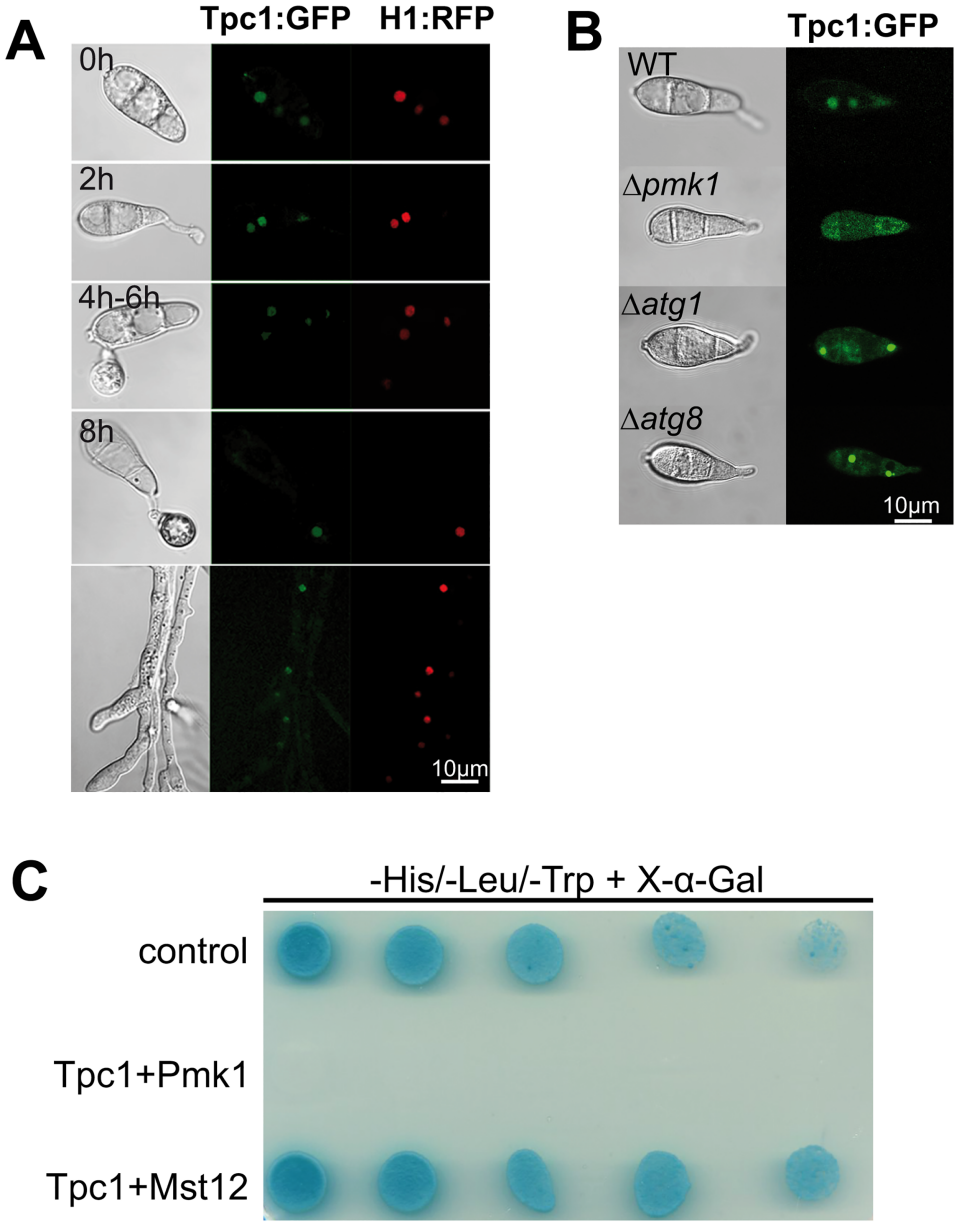
Tpc1 shows steady state nuclear localisation and is regulated by the Pmk1 signalling pathway. **(A)** Nuclear localisation of Tpc1:GFP during infection-related development and vegetative hyphae by confocal microscopy. Conidia and mycelium from a WT strain expressing both histone *H1*:*RFP* and *TPC1*:*GFP* protein fusions. **(B)** Confocal images of conidia harvested from *M*. *oryzae* Δ*pmk1*, Δ*atg1 and* Δ*atg8* mutants expressing *TPC1*:*GFP*. Tpc1 is not visualized in the nuclei of Δ*pmk1* mutant. **(C)** Yeast two-hybrid experiment showing Tpc1 interacts with Mst12 but not with Pmk1. Yeast colonies co-transformed simultaneously with the pGAD-TPC1 (bait Tpc1), and pGBK-Pmk1 and pGBK-Mst12 (prey Pmk1 and Mst12) vectors grow in high stringency media (-His/-Ade/-Leu/-Trp/+X-α-Gal) at specified concentrations: 1 x 10^6^, 1 x 10^5^, 1 x 10^4^, 1 x 10^3^, 1 x 10^2^ cells, each a 10μl droplet. The interaction of proteins expressed by prey and bait vectors generates a blue-coloured colony due to the activation of α-galactosidase expression in the presence of X-α-Gal. Positive control cells contain pGBKT7-53 and pGADT7-T plasmids co-transformed into Y2HGold.

The Tpc1:GFP fusion protein co-localised with histone H1:RFP in nuclei of vegetative hyphae, attached conidia (30 min), and germinated conidia (Fig 5A). Moreover, whenever Tpc1:GFP was observed in nuclei, GFP fluorescence was never observed in the cytoplasm or other organelles within conidia. The results are consistent with *TPC1* encoding a transcription factor that acts within the nucleus during the initial stages of spore germination and appressorium development, and correlate with the observed overexpression of *TPC1* in these fungal structures [29].

To investigate whether *TPC1* is associated with specific or multiple regulatory networks, *TPC1*:*GFP* localisation was observed in conidia of different mutant backgrounds (Fig 5B). In the Δ*pmk1* MAPK mutant[39], Tpc1:GFP was observed within the cytoplasm but not in nuclei. By contrast, strong GFP fluorescence was visualised in conidial nuclei of the autophagy-defective Δ*atg1* and Δ*atg8* mutants, compared to the fluorescence observed in M1422 complemented with *TPC1*:*GFP* or Guy11 expressing *TPC1*:*GFP*. These results suggest that Tpc1 activity is associated with the Pmk1 MAP kinase signalling pathway, which regulates appressorium formation[39], and the control of autophagy[34].

We further analysed the link with between Tpc1 and the Pmk1 pathway by looking at the ability of Tpc1 to interact with components of this pathway in a yeast two-hybrid system. Strikingly, we observed that Tpc1 interacted with Mst12, a transcription factor that functions downstream of Pmk1[40], although Tpc1 did not interact with Pmk1 itself (Fig 5C). The mis-localisation of Tpc1 in Δ*pmk1* and its interaction with Mst12 strongly support Tpc1 involvement in this pathogenicity-associated MAPK signalling cascade.

### Tpc1-like transcriptional regulators are found exclusively in filamentous fungi

We investigated the phylogenetic relationship of Tpc1 to other putative *Magnaporthe* Zn(II)_2_Cys_6_ proteins and the closest orthologues of Tpc1 in other fungal species (S4 and S5 Figs). We observed that the six cysteine residues of the DNA-binding domain (DBD) in the Zn(II)_2_Cys_6_ proteins were ordered in a conserved pattern, CX_2_CX_6_CX_5-12_CX_2_CX_6-8_C (S4A and S4B Fig). In *M*. *oryzae*, the Zn(II)_2_Cys_6_ binuclear cluster family is diverse and composed of 175 members (S2 Table). The closest orthologues of Tpc1 (MGG_01285) were identified using BLASTP and used to construct a phylogenetic tree (S4C and S5 Figs). Tpc1 clustered in a group with sequences from other Sordariomycetes, such as *Fusarium graminearum*, *N*. *crassa*, *Chaetomium globosum* and *Podospora anserina*. Based on this tree, Tpc1 is a single copy gene and has not been subject to paralogous duplications. Our phylogenetic analysis reflected the diversification of the Zn(II)_2_Cys_6_-containing proteins in the fungal kingdom. Interestingly, we did not find putative homologues of Tpc1 in *S*. *cerevisiae* or *Schizosaccharomyces pombe* using a BLASTP search.

In *F*. *graminearum*, it is remarkable that only 16% (46/296) of the mutants lacking Zn(II)_2_Cys_6_ transcription factors showed a phenotype, compared to the 42% (30/72) of *N*. *crassa* mutants or the 59% (61/104) of *M*. *oryzae* mutants[28,41,42]. Among the *F*. *graminearum* mutants with clear phenotypes is found the orthologue of *M*. *oryzae TPC1* (*FgTPC1* = FGSG_08769; GzZC108), which is required for plant infection, perithecia formation, synthesis of mycotoxins (ZEA, zearalenone; and DON, deoxynivalenol) and growth[41]. Similar to *M*. *oryzae* (Fig 1E and S2F Fig), the Δ*fgtpc1* mutant is more resistant than wild-type to hyperosmotic and oxidative stresses.

We further investigated functional conservation of Tpc1 in the saprotrophic filamentous fungus *N*. *crassa*, and characterized a *N*. *crassa NcTPC1* deletion mutant (Δ*nctpc1*; NCU05996), obtained from the Fungal Genetic Stock Centre[43]. The analysis of the alignment of *M*. *oryzae* and *N*. *crassa* Tpc1 proteins showed that they share 67% amino acid identity (S6A Fig). Strikingly, the Δ*nctpc1* mutant was severely reduced in vegetative growth compared to an isogenic wild-type strain (p <0.01) (S6B Fig), and its vegetative hyphae also formed compacted colonies. In addition, we observed that the Δ*nctpc1* mutant of *N*. *crassa* was less severely affected when exposed to increasing osmotic stress using NaCl, compared with the *N*. *crassa* wild-type strain (p <0.01) (S6C Fig). Similar tolerance effect was also found in *F*. *graminearum* Tpc1[41] and in *M*. *oryzae* Tpc1 (Fig 1E). Consequently, *N*. *crassa* Tpc1 also plays a significant role in growth and development of the fungus and its responses to abiotic stress.

### Oxidation-reduction processes are significantly affected in the Δ*tpc1* mutant

Tpc1 contains a Zn(II)_2_Cys_6_ binuclear cluster DNA binding domain, which is found only in fungal proteins considered bonafide transcription regulators[13,14]. We carried out a comparative transcriptome analysis using the wild-type strain and the *TPC1* deletion mutant to identify the biological processes and genes regulated by Tpc1. For this experiment, RNA was extracted from fungal material grown on cellophane on top of CM agar plates (S2D Fig). We considered it to be an optimal condition since fungal hypha is able to penetrate the cellophane, i.e. a change in polar growth occurs under these conditions, and allow us to obtain enough amount of RNA for subsequent microarray analysis. We identified 215 down-regulated genes and 185 genes to be up-regulated with at least a two-fold change in expression level in the Δ*tpc1* mutant (S3 Table). We classified all the genes that were up- and down-regulated into four functional groups according to potential roles in signalling (13 genes), cell wall biosynthesis or modulation of plant response (secreted proteins; 140 genes), metabolism (127 genes) and other functions (54 genes). Sixty-six genes encoded proteins that lacked any known domain. Remarkably, two gene ontology (GO) terms were found significantly enriched among these differentially expressed genes, the oxidation-reduction process (GO:0055114; 57 genes; p<0.001) and the oxidoreductase activity (GO:0016491; 58 genes; p<0.001).

Within the signalling functional group, two down-regulated genes encoded phosphatidyl ethanolamine-binding proteins (PEBP) that have been shown to regulate protein kinase A (PKA) and mitogen-activated protein kinase (MAPK) pathways[44,45]. Amongst the up-regulated genes, eight of them encoded transcriptional regulators, which suggests a link between the gene networks controlled by these transcriptional regulators and Tpc1.

The largest group of mis-regulated genes comprised 140 genes coding for secreted or cell wall-related proteins. Within this group, more than half of the members (74 genes) had no matches in databases. However, twenty-four genes were potentially involved in cell wall remodeling, and encompassed different types of glycosyl hydrolases (GH10, GH18, GH32, GH43, GH61 and GH81), seventeen proteases and two secreted phospholipases A2. Three Mas3/Gas1 paralogues and several effector proteins such as a Bas2-like, Bas113 and avrPi54 were also found[46,47,48,49]. The up-down regulation of two CFEM G-protein coupled receptors[50], including *PTH11*[51], suggested an alteration in the ability of the Δ*tpc1* mutant to perceive external signals.

The second largest group of genes with altered expression levels encoded proteins related with primary and secondary metabolism (127 genes). We found a significant number of them participating in oxidation-reduction processes (46%) and transport (9%). Alteration in nitrogen and glycerol metabolism was evidenced by the expression changes of four NmrA-like regulatory proteins[52], enzymes involved in amino acid biosynthesis, a glycerol kinase and the glycerol dehydrogenase Gcy1, an enzyme also associated with redox regulation in yeast[53]. Down-regulation of an α-glucosidase supported the glycogen degradation delay of Tpc1-lacking strains.

The last functional group included 54 genes encoding proteins that carry a wide range of biochemical roles. The reduced expression of the autophagy gene *ATG22* and the up-regulation of three small chaperones Hsp20-like suggested the unbalanced signals for survival and cell death existent in Δ*tpc1*[54]. Microtubule-dependent vesicle trafficking and cell cycle were also affected in the mutant as inferred from the misregulation of two dynamins, one kinesin light chain, one Marvel protein, two cyclins and the Cdc26 subunit. Genes involved in silencing pathways, spliceosomal snRNP assembly, tRNA processing, RNA-mediated heterochromatin silencing and translational arrest were also misregulated in Δ*tpc1*, highlighting alterations in other cellular processes that regulate gene expression.

### Gene-deletion analysis of down-regulated transcripts in Δ*tpc1* identifies a major pathogenicity gene in *M*. *oryzae*

The majority of the Zn(II)_2_Cys_6_ binuclear cluster proteins are transcriptional activators and only few of them have been shown to act as repressors[14]. To identify novel pathogenicity genes we focused on genes that could play a role in *TPC1*-associated defects. Five out of the 133 down-regulated genes were selected for gene replacement (S3 Table; S7 Fig), including the conidiation-related gene *CON6*[55], a glycosyl transferase 18 gene (*GH18*) that undergoes a 50-fold increased expression *in planta*[48], and the two signaling-associated *PEBP* genes (S7 Fig). The *PRO41/HAM-6* gene, which is required for hyphal fusion in *Neurospora crassa* and sexual development in *Sordaria macrospora* was also selected for the analysis[56,57]. We confirmed by RT-PCR that the five genes were down-regulated in the Δ*tpc1* mutant (S8A Fig).

Among the six deletion mutants generated, only Δ*pro41*/Δ*ham-6* displayed a severe pathogenicity-deficient phenotype (S7 Fig). Despite the links found between conidiogenesis and pathogenicity in *M*. *oryzae*[58,59,60], the Δ*con6* mutant behaved like wild-type *in planta*. Similarly, Δ*gh18*, Δ*pebp1*, Δ*pebp2*, and the double mutant Δ*pebp1*Δ*pebp2* did not show any pathogenicity-associated defects possibly due to redundancy in related gene functions. Consequently, we selected Δ*pro41*/Δ*ham-6* mutants for further characterization.

### *M*. *oryzae NOXD/PRO41* is required for superoxide production, sexual development and plant penetration

The open reading frame of *M*. *oryzae PRO41* was initially annotated in the EnsemblFungi database as *HAM-6*, a *N*. *crassa* gene required for cell fusion[61]. However, the orthologue of this protein was first characterized in *S*. *macrospora* and named Pro41[57,62]. Pro41 is a novel ER membrane protein required for fruiting body maturation in *S*. *macrospora*. Later, Pro41 was found to be the functional orthologue of the p22^phox^ subunit of the NADPH oxidase complex in both *Podospora anserina* and *Botrytis cinerea*[63,64]. Therefore, we renamed the Pro41/Ham-6 protein NoxD (S8B Fig).

**Fig 6:**
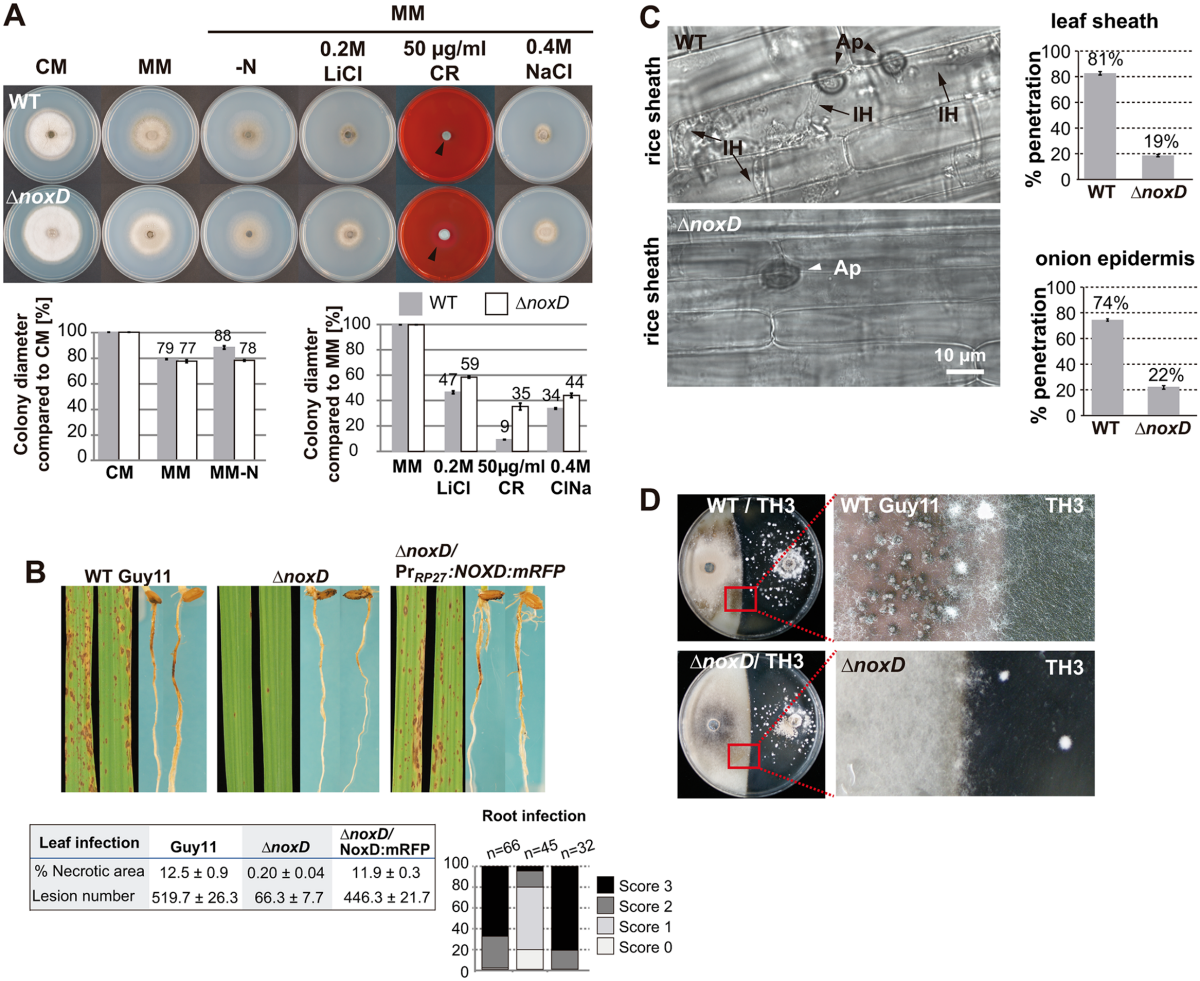
The *M*. *oryzae* NoxD protein is required for plant penetration and sexual reproduction. **(A)** Eight-day old wild-type (WT) Guy11 and Δ*noxD* strains grown on different media and stress conditions (additional information in S8C and S8D Fig). The Δ*noxD* mutant showed accelerated growth rate compared to wild-type (WT) on CM. Δ*noxD* growth is affected in MM-N media and shows increase resistance to salts (LiCl, ClNa) and CongoRed (CR). Colony edge is indicated with a black arrowhead. **(B)** Leaf and root infection assays showing Δ*noxD* pathogenicity defects and functional complementation of Pr_RP27_:*NOXD*:mCherry construct. **(C)** Penetration assays using leaf sheaths and onion epidermal cells. It is very unusual to observe Δ*noxD* within the host cells. **(D)** Fertility assay in oatmeal agar plates pairing Guy11 and Δ*noxD* with the tester strain of opposite mating type TH3. No perithecia were observed from the cross of Δ*noxD*×TH3.

We looked at the growth of the *M*. *oryzae ΔnoxD* mutant in different media and stress conditions (Fig 6A and S8C Fig). Δ*noxD* grew slightly faster than the wild-type on CM and MM, under salt stress (0.2 M LiCl, 0.4 M NaCl) and in Congo Red (CR). However, we did not observe differences in growth under carbon starvation, calcofluor white (CFW) or basic conditions (pH 9.5). Increased resistance to CFW was previously observed for *M*. *oryzae* Δ*nox1* but not for Δ*nox2*[65], suggesting NoxD and Nox2 fulfill similar roles during cell wall biogenesis. Growth of *M*. *oryzae* Δ*noxD* and Δ*nox1*Δ*nox2* mutants in 1mM methyl viologen, 1mM H_2_O_2_ and 5mM H_2_O_2_ was similar or improved when compared to wild-type (S8D Fig). Thus, the lack of NoxD did not affect fungal growth under oxidative stressors in contrast to the growth defects displayed by *B*. *cinerea* NADPH oxidase mutants[64].

The infection ability of Δ*noxD* was severely affected on leaves and roots (Fig 6B and S7 Fig), in accordance with the strong penetration defects displayed by *Δnox1* and *Δnox2*[65]. The penetration defect displayed by Δ*noxD* was confirmed using rice leaf sheaths (81% in the wild-type versus 19% in the mutant) and onion epidermis penetration assays (74% in the wild-type versus 22% in the mutant; Fig 6C). Subsequently, we crossed the Δ*noxD* mutant with the rice isolate TH3, a *M*. *oryzae* strain of opposite mating type (Fig 6D). The inability to produce perithecia indicated that NoxD is required for sexual reproduction in *M*. *oryzae*.

**Fig 7:**
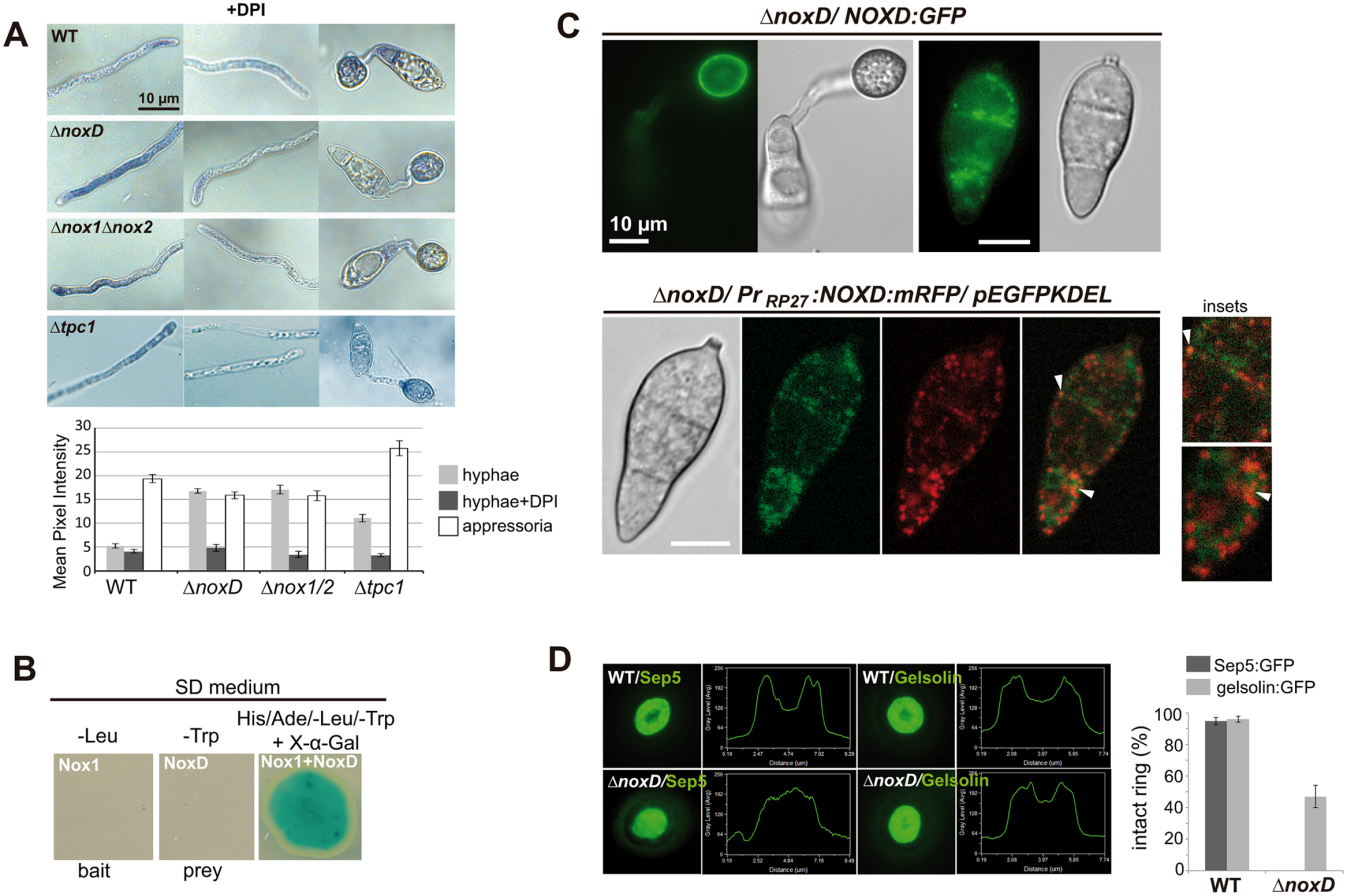
*M*. *oryzae* NoxD is required for ROS production in appressoria, localizes in ER-derived vesicles and plasma membrane, and interacts with Nox1. **(A)** Superoxide production is significantly affected in the mutants as shown by nitroblue tetrazolium (NBT) staining and quantification of pixel intensities in hyphal tips (mean±SD; n = 10). Both Δ*noxD* and Δ*nox1*Δ*nox2* produced less ROS in appressoria but higher amounts of ROS in hyphal tips compared to WT; Δ*tpc1* generated higher amounts of ROS in hyphal tips and appressoria. Addition of diphenylene iodonium (DPI) before NBT treatment abolishes the formation of dark precipitates associated with sites of superoxide generation. Values to calculate mean pixel intensity were for white 0 and black for 100. **(B)** A yeast two-hybrid screen reveals that NoxD interacts with Nox1. A yeast colony co-transformed simultaneous with the pGBK-NOX1 (bait Nox1) and pGAD-NOXD (prey NoxD) vectors grows in high stringency media (-His/-Ade/-Leu/-Trp/+X-α-Gal). The interaction of proteins expressed by prey and bait vectors generates a blue-coloured colony due to the activation of α-galactosidase expression in the presence of X-α-Gal. **(C)** Images of *NOXD* fusion constructs using GFP and mRFP under two different promoters, the *NOXD* promoter and the strong promoter of ribosomal protein Rp27, respectively. Both NoxD fusions localized in vesicles and plasma membrane. In conidia expressing *NOXD*:*mRFP*, fluorescence signal is observed in vesicles closely associated to ER membranes labelled with GFP containing the ER retention signal KDEL. After 8h, fluorescence signal is strongly detected at the plasma membrane of the appressoria. pEFGPKDEL encodes GFP with the ER retention signal KDEL. **(D)** Micrographs of F-actin ring organization visualized by expression of gelsolin:GFP and Sep:GFP in WT and Δ*noxD*. This mutant produces aberrant septin and actin rings; mis-localization of Sep5:GFP is more severe. The linescan graphs show fluorescence in a transverse section of individual appressoria.

To define whether superoxide production was impaired in the *ΔnoxD* mutant, we used nitroblue tetrazolium (NBT), which forms a dark-blue water-insoluble formazan precipitate upon reduction by superoxide radicals[65,66]. In the Δ*noxD* mutant, we observed an increase in superoxide production at hyphal tips and a significant reduction in appressoria based on mean pixel intensity measurements (p<0.01) (Fig 7A). This was previously described for Δ*nox1*Δ*nox2* mutants[65], and supports the existence of alternative routes for cellular ROS generation in *M*. *oryzae* during hyphal development. Since *Δtpc1* was affected in oxidation-reduction processes, we also included Δ*tpc1* in this analysis. Increased superoxide production was found in *Δtpc1* hyphal tips but to a lesser extent than *nox* mutants, while in appressoria *Δtpc1* showed the highest superoxide levels among the strains analyzed, indicating that the lack of Tpc1 affects superoxide production pathways in *M*. *oryzae*.

A yeast two-hybrid assay was used to identify putative NoxD interactors. We found that *M*. *oryzae* NoxD interacts with the Nox1 NADPH oxidase subunit (Fig 7B) but not with Nox2 or NoxR, supporting previous work in *B*. *cinerea* and *P*. *anserina*[63,64].

### NoxD is visualized in ER-associated vesicles and plasma membrane of appressoria and conidia

To localize NoxD we generated C-terminal mRFP (cherry variant) and GFP translational fusions under the control of strong or native promoters, respectively. Both constructs fully complemented Δ*noxD* plant infection defects (Fig 6B), which indicated that the C-terminal tag does not affect NoxD function, although expression of NoxD:mRFP was clearly stronger. *M*. *oryzae* NoxD was mainly observed in subapical vesicles and the plasma membrane of appressoria and conidia (Fig 7C). Co-localization of NoxD:mRFP with GFP containing the ER retention signal KDEL showed that the vesicles are closely associated with the ER, overlapping with some of them (Fig 7, white arrowheads). The subapical vesicles observed near plasma membranes and septa in *M*. *oryzae* structures correlated with the localisation of NoxD in *P*. *anserina* [63]. In *P*.*anserina*, these vesicles co-localised with the GFP:Idi7 reporter protein, suggesting that they originate from the ER and travel towards the vacuolar system [63].

### NoxD is required for septin ring assembly at the appressorial pore

The Nox2-NoxR complex is essential for septin-mediated cytoskeletal reorientation, whereas Nox1 is dispensable although may have important roles to play in maintenance and elongation of the penetration peg[6]. To test if NoxD was also involved in this process, we expressed the acting-binding protein gelsolin:GFP and Sep5:GFP in Δ*noxD*. In the wild-type, both a septin and gelsolin ring was present at the appressorium pore[6] (Fig 7D). In the Δ*noxD* mutant, however, Sep5:GFP formed a disorganized mass in the infection cell as previously reported for Δ*nox2* and Δ*noxR* expressing Sep5:GFP[6]. Gelsolin:GFP rings in Δ*noxD* also possessed distorted pores. Considering that gelsolin colocalizes with F-actin at the appressorial pore[6], the altered fluorescence pattern of gelsolin:GFP revealed that the toroidal F-actin ring was disorganized (Fig 7D). Previous reports showed that Sep5:GFP and gelsolin:GFP patterns in the Δ*nox1* mutant displayed normal conformation[6]. NoxD and Nox1 therefore appear to play alternative roles in cytoskeletal re-modeling in appressoria of *M*. *oryzae*.

### NOXD expression is regulated by Tpc1 and the Pmk1 pathway

**Fig 8:**
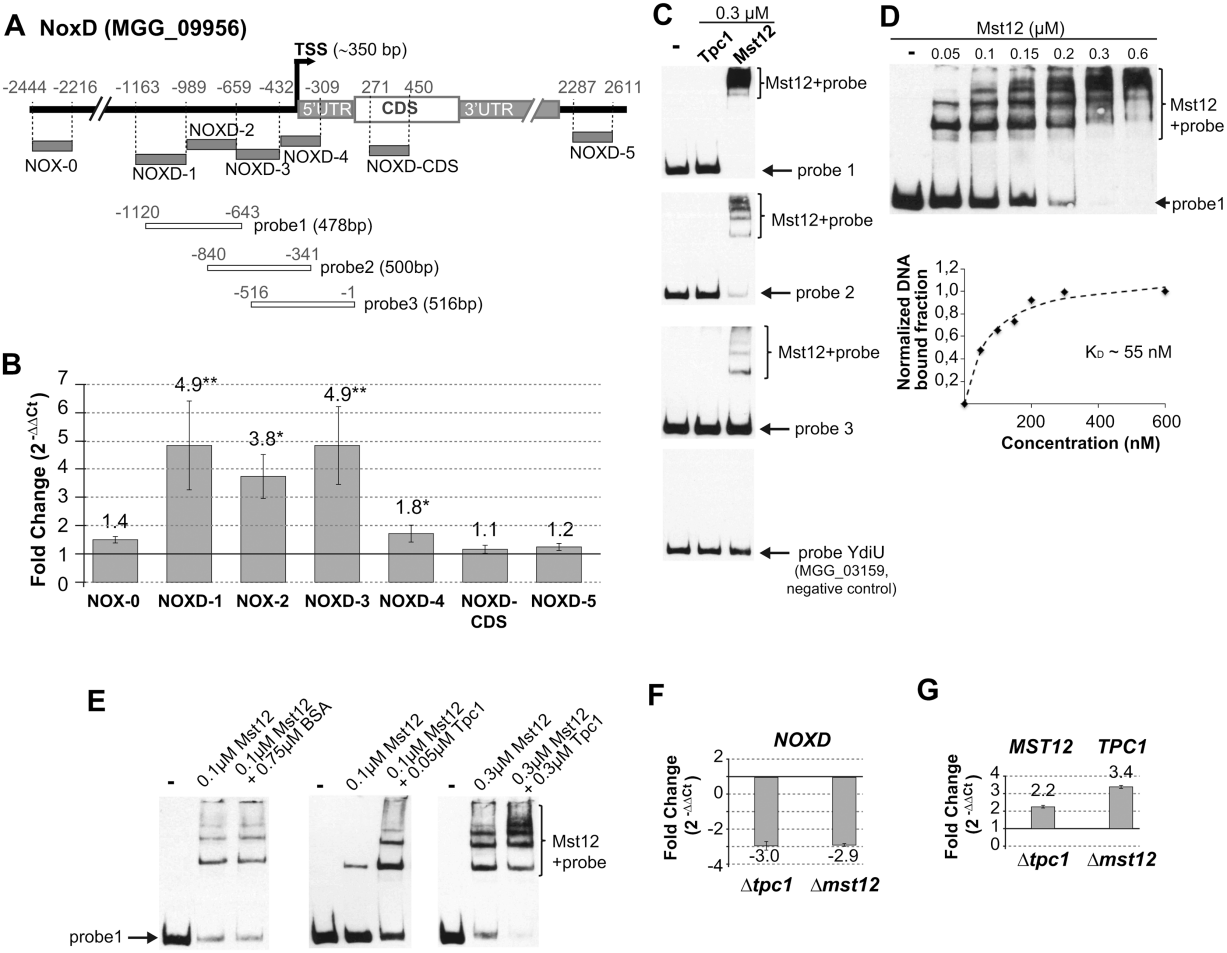
Tpc1 and Mst12 bind the promoter region of the *NOXD* gene. **(A)** Genomic location of fragments used for chromatin immunoprecipitation (ChIP) and electrophoretic mobility shift assays (EMSA). Transcription start site (TSS) was identified by 5’-end RACE (rapid amplification of cDNA ends) followed by sequencing. The 3’UTR length was estimated using EST data. **(B)** ChIP analysis with anti-GFP antibody was used to identify Tpc1:GFP levels associated with *NOXD* promoter (means±SD, n = 4). DNA isolated from chromatin immunoprecipitated with anti-GFP antibody was subjected to qPCR. The DNA in ChIP fractions prepared without antibody was used as negative control. The ratio of DNA in ChIP fractions with respect to that in the untagged wild-type cell extracts was calculated for *NOXD* region and normalized with the values for the *NOXD* site. The normalized ratios were plotted in the lower panel. Probes indicated by asterisks are statistically significant according to the Wilcoxon-Mann-Whitney test and have a *p* value <0.05 (*) or <0.01 (**). **(C)** EMSA with Mst12 and Tpc1. Mst12 recognises strongly probe 1, and with less affinity probes 2 and 3. Tpc1 cannot directly bind to the *NOXD* promoter. The promoter region of a YdiU-containing protein was used as a negative control. **(D)** DNA-binding analysis of Mst12 to *NOXD* promoter. Dissociation constant (K_D_) was estimated using bound probes against protein concentration and the fitting equation y = mx/(k+x). **(E)** DNA-binding experiments showing Tpc1 promoting Mst12 DNA-binding affinity to NoxD probe 1 using different amounts of Tpc1. BSA (bovine serum albumin) resuspended in Tpc1 buffer was used as a negative control; BSA does not stimulate Mst12 binding to the probe. **(F)** Analysis of *NOXD* transcript abundance in Δ*tpc1* and Δ*mst12* mutants by qPCR. **(G)** Transcript abundance of *MST12* and *TPC1* in Δ*tpc1 and* Δ*mst12* backgrounds, respectively. cDNA concentration in the mutants was normalised using actin and wild-type levels of the genes analysed (referred as 1; mean±SD, n = 3).

The down-regulation of *NOXD* in Δ*tpc1* suggested that this gene may be directly regulated by Tpc1. To investigate this idea, we carried out chromatin immunoprecipitation (ChIP) followed by qPCR (Fig 8A and 8B). We observed that the promoter region of *NOXD* comprising the NOXD1, NOXD2 and NOXD3 fragments immunoprecipitated with Tpc1:GFP, which indicated that *NOXD* expression is regulated *in vivo* by this transcription factor. In addition, we performed electrophoretic mobility shift assays (EMSA) with *M*. *oryzae* Tpc1 and Mst12 since both proteins can interact in yeast two-hybrid assays (Fig 5C). We found that Mst12 strongly recognised the probe 1 located between -1120 and -643 upstream of the start codon of the *NOXD* gene (Fig 8C). Mst12 also recognized probes 2 and 3, but less strongly. Mst12 bound to the probes produced multiple bands, possibly due to the presence of several protein molecules on the biotinylated DNA (Fig 8D). Intriguingly, Tpc1 itself was not capable of recognizing any of the three probes under the conditions tested (Fig 8C). However, the addition of Tpc1 to Mst12 increased its DNA-binding capacity (Fig 8E), which is consistent with both, the ability of these proteins to interact, and with Tpc1 as modulator of Mst12 DNA-binding affinity. Increasing amounts of Tpc1 did not alter significantly Mst12affinity. Importantly, the promoter regions tested using these *in vitro* DNA-binding assays correlated perfectly with the enriched fragments obtained in the ChIP analysis, which supports that Mst12 and Tpc1 are part of a complex that coordinately regulate NoxD expression. To further confirm these results, we checked *NOXD* expression levels in the Δ*mst12* mutant and corroborated that they were reduced (Fig 8F). We also observed that *MST12* and *TPC1* genes were overexpressed when the corresponding partner was not present in the fungal cell (Fig 8G). We conclude that Tpc1 regulates NoxD expression through its interaction with Mst12 and confirm the link between Tpc1 and the participation of the Pmk1 pathway in the regulation of NoxD expression.

## Discussion

To cause disease in rice, *M*. *oryzae* forms a specialised cell called an appressorium, the development of which involves transitions from polarised to isotropic cellular growth, followed by rapid turgor-driven polarisation to penetrate the leaf surface. Understanding how these cellular transitions occur is critical to controlling the disease at an early stage, prior to entering the plant. In this study, we have identified a transcription factor, Tpc1 that plays a key role in regulating plant infection, due to its role in polarity control. We have also identified one putative mechanism by which it acts, via the regulated synthesis of reactive oxygen species and control of the NADPH oxidase complex, which regulates septin assembly and F-actin re-modelling at the base of the appressorium. Furthermore, we have found that Tpc1 directly participates in the Pmk1 pathway and is required for infection-associated autophagy, which are both essential pre-requisites for appressorium formation and function.

**Fig 9:**
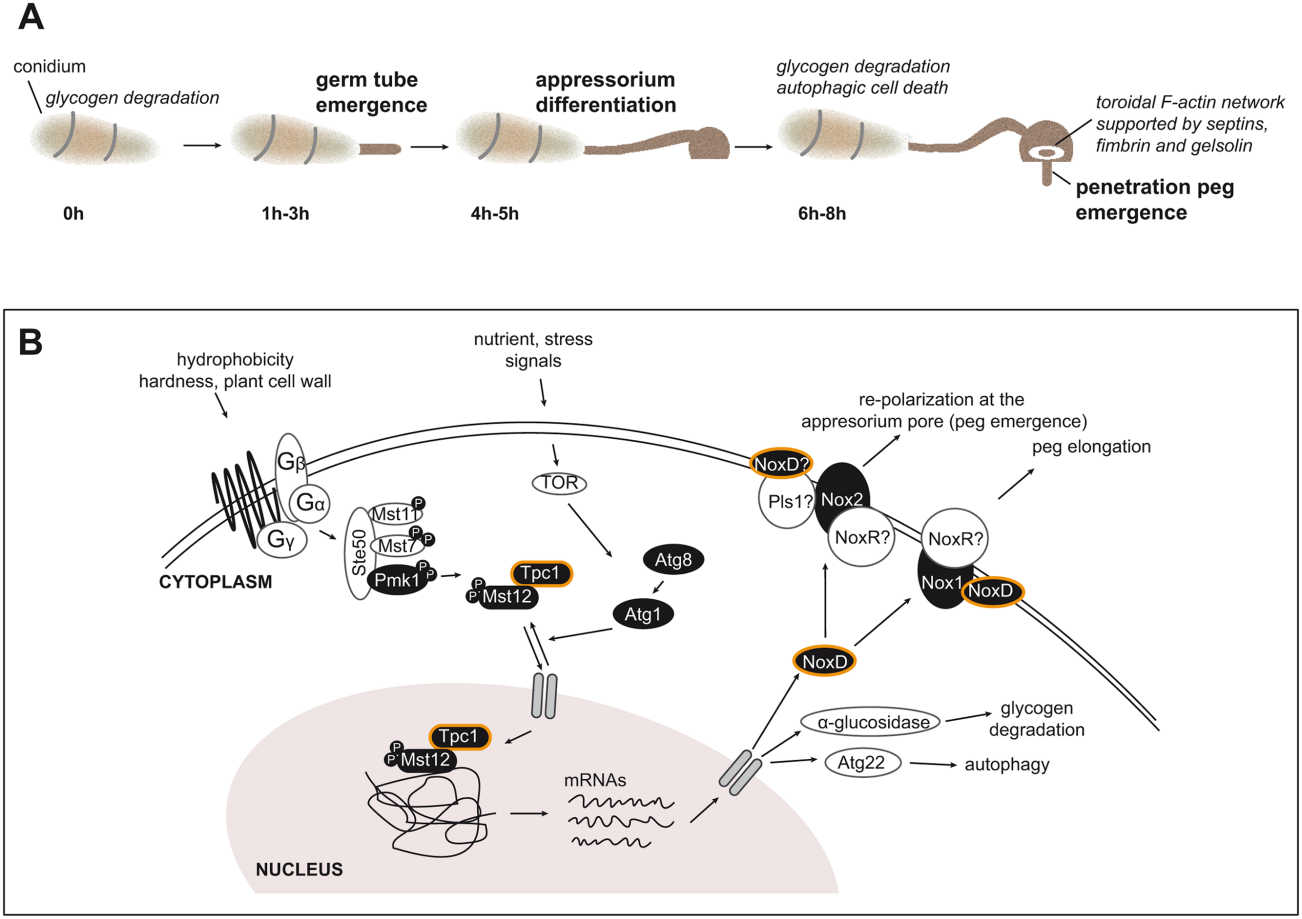
Polar growth and associated processes that take place during appressorium maturation in *M*. *oryzae* and model of Tpc1-mediated plant penetration. **(A)** Polarity factors regulate the emergence and elongation of the germ tube and the penetration peg required for appressorium development and function. Polarity is concomitant with autophagic cell death and glycogen/lipid degradation–cellular processes that also control appressorium function. **(B)** During germination, environmental signals trigger the Pmk1 kinase cascade and the control of autophagy to activate Tpc1 function. Nuclear-localised Tpc1 in turn activates transcription of genes required for polar growth, autophagy, and glycogen degradation. Tpc1 interacts with Mst12 and this complex is required for regulation of the expression of several genes, including NoxD. Polarity is associated with cytoskeletal dynamics that is controlled by Tpc1 through the Nox1 and Nox2 NADPH oxidase complexes. NoxD interacts with Nox1 and maybe with Nox2 indirectly. Interactions of NoxR and Pls1 with Nox1 and/or Nox2 need to be confirmed. To initiate plant infection Nox1/Nox2 NADPH oxidases regulate the formation of the F-actin network at the appressorium pore, which leads to penetration peg emergence and subsequent elongation. Mutant strains used in this study are highlighted in black.

We observed that the *TPC1* mutants formed compact colonies, which resembled the colony morphology shared by a class of mutants with polarity defects in *N*. *crassa*[31]. Conidial germination, and growth of vegetative hyphae were severely impaired in the two mutants lacking functional Tpc1, supporting defects in sustained tip elongation and establishment of polarity in apically-growing hyphae. Autophagy plays a major role in supplying amino acids, fatty acids, and glucose to maintain cellular functions during stress and starvation[67]. The absence of Tpc1 function altered the onset of infection-associated autophagy which occurs during appressorium development[68]. Conidial cell death is necessary to initiate appressorium penetration and it is regulated by the Pmk1 pathway [33]. Although M1422 conidia appeared able to undergo conidial cell death, the cellular localization of autophagosomes and glycogen/lipid deposits suggested that the process was delayed. Consistent with this observation, Tpc1:GFP was also highly expressed in *Δatg1* and *Δatg8* mutants impaired in autophagy, suggesting that the expression of *TPC1* is de-repressed as a consequence of the inability to carry out autophagy and may therefore be an upstream positive regulator of infection-associated autophagy during appressorium maturation (Fig 9).

Autophagic cell death is linked with appressorium function and penetration in *M*. *oryzae*[4], and mutants lacking Tpc1 are also penetration defective. The formation of a penetration peg at the base of the appressorial pore is a cellular process intrinsically linked to polar growth[69,70]. The F-actin cytoskeleton plays a crucial role during germ tube re-polarisation and penetration peg emergence. We therefore investigated cytoskeletal dynamics during appressorium maturation in the mutant background. The network of F-actin observed with fimbrin:GFP in mature wild-type appressoria (8h-24h) delineated the appressorial pore, which was strikingly absent in M1422. This result together with the mis-localization of Sep5:GFP and gelsolin:GFP in Δ*tpc1* indicated that the F-actin network is disturbed in Tpc1-lacking strains.

The microarray analysis helped us to identify gene expression changes due to the lack of Tpc1, which correlated with the observed involvement of this protein in glycogen metabolism, autophagy and polar growth. Oxidation-reduction processes were also significantly affected in Δ*tpc1* including superoxide production pathways, likely due in part to the down-regulation of the fungal homologue of the p22^phox^ NADPH subunit, the *NOXD* gene[63,64]. The *M*. *oryzae* Δ*noxD* mutant was unable to infect rice leaves and roots. We established an interaction of NoxD with Nox1, but not with NoxR or Nox2, and confirmed the requirement of NoxD for superoxide generation and sexual reproduction in *M*. *oryzae*, consistent with NoxD functions in *B*. *cinerea* and *P*. *anserina*[63,64]. We also identified Δ*noxD* defects in repolarization of the F-actin cytoskeleton during infection-related development, supporting the previous role described for the *M*. *oryzae* NADPH oxidase complex[6]. Remarkably, the disorganization pattern of gelsolin:GFP and Sep5:GFP in Δ*noxD* was similar to that observed in Δ*nox2* and Δ*noxR* mutants, whereas Δ*nox1* formed nearly intact ring shapes[6]. This result suggests that Nox1 and NoxD participate differently in septin-mediated cytoskeleton organization despite their interaction, and strengthens the view of the fungal NADPH oxidase as a dynamic complex[71]. It seems likely that a Nox2-dependent process initiates septin ring formation, while Nox1 is necessary for maintenance of this conformation (Fig 9). NoxD may therefore be associated at a relatively early stage in recruiting Nox1 to the appressorium pore, perhaps explaining why its absence results in a more severe phenotype with respect to actin and septin assembly at the pore. The role of NoxD, however, highlights that the Nox1 and Nox2 complexes are both necessary for penetration peg elaboration and extensive polar growth. It is worth noting that the tetraspanin *PLS1* deletion mutants exhibit the same phenotype as Δ*nox2* in *M*. *oryzae* and *P*. *anserina*[6,72], suggesting that Pls1 may act as the missing link between Nox2 and NoxD subunits of the fungal NADPH oxidase complex (Fig 9). The recent discovery in *B*. *cinerea* of the RasGAP protein homologue IQGAP and its interaction with NoxD also points to IQGAP as a scaffold protein of the fungal NADPH complex[73]. In mammals, Nox complexes can act upstream[74] or downstream [75] of MAPK signaling pathways. IQGAP also interacts with different modules of MAPK- and Ca-dependent signalling cascades[73], pointing the link between Nox complexes and signalling cascades. Interestingly, the *B*. *cinerea* Δ*noxD* mutant showed growth defects in the presence of oxidative stressors in contrast to the wild-type growth exhibited by the *M*. *oryzae* Δ*noxD* mutant, which suggests a diversification of the cellular functions of NoxD in fungi. This result also hints differences in the regulation of ROS-mediated signalling pathways in the fungal kingdom.

Importantly, two lines of evidence support the direct involvement of Tpc1 in NoxD expression regulation. The ChIP analysis demonstrates that Tpc1:GFP immunoprecipitates *in vivo* with the *NOXD* promoter region. Tpc1 also regulates Mst12 DNA-binding activity *in vitro* using the corresponding *NOXD* promoter region, and indicates a direct participation of Tpc1 in the MAPK Pmk1 signalling pathway. Despite the ability of Tpc1 and Mst12 to regulate *NOXD* expression together, and their participation in common cellular processes such as penetration peg formation and plant invasion[9], Δ*mst12* and Δ*tpc1* mutants have different colony morphology. In contrast to Tpc1, Mst12 is dispensable for growth and appressorial turgor generation[9]. Consequently, Tpc1 has the ability to modulate expression of genes that participate in additional cellular processes, either by interacting with other transcription factors, or activating directly the expression of different genes.

Here, we identified one potential mechanism by which the transcription factor Tpc1 regulates appressorium maturation and plant infection. The loss of pathogenicity associated with *M*. *oryzae* and *F*. *graminearum TPC1* mutants and similar growth defects associated with the *N*. *crassa* Δ*nctpc1* mutant, suggest that Tpc1 plays a key role as a transcriptional regulator in the re-establishment of polarity and the response to numerous signalling pathways, such as the Pmk1 MAP kinase and Atg1 kinase cascades. The role of Tpc1 in appressorium-mediated plant infection appears to be associated with the NADPH oxidase-dependent re-polarisation process of the appressorium, and the associated physiological changes such as autophagy, glycogen/lipid mobilisation and asymmetric reorganization of the F-actin cytoskeleton. Future studies will allow further dissection of this role and precise definition of the biological processes regulated by Tpc1 in filamentous fungi.

## Materials and methods

### Strains, media and plant infections

*M*. *oryzae* was routinely incubated in a controlled temperature room at 25°C with a 12h light/dark cycle. Media composition of complete medium (CM), minimal medium (MM), minimal medium without carbon (MM-C) or nitrogen (MM-N), and DNA extraction and hybridisation were all as previously described[76]. Growth tests were carried out with 7 mm plugs of mycelium from Guy11 and the M1422 mutant strains as initial inoculum. The wild-type *Neurospora crassa* strain and isogenic deletion mutant NCU05996 were obtained from the Fungal Genetics Stock Centre (FGSC, Kansas City, Missouri, USA). Vogel’s minimal medium was used for cultivation of *N*. *crassa* strains at 25°C with a 12h light/ dark cycle and for stock-keeping at 4°C (http://www.fgsc.net/Neurospora/NeurosporaProtocolGuide.htm). Growth tests were carried out on Vogel plates with 5 mm plugs of mycelium from *N*. *crassa* wild-type (wt) and *NcTPC1* KO strains. Plates were incubated at 25°C for 2 days. *M*. *oryzae* leaf and root infection assays were carried out, as previously described [30,77].

### Conidiation, onion/leaf sheath penetration assays, cytorrhysis assay and glycogen/Nile red staining

Conidia were harvested using 2 ml of sterile water per plate after fungal cultures were incubated at 25°C for a period of 10 days on CM. Calculations were then carried out to determine the number of conidia generated *per* cm^2^ of mycelium using a Neubauer counting chamber. Values are the mean ± SD from >300 conidia of each strain, which were measured using the ImageJ software [78]. Photographs were taken using the Zeiss Axioskop 2 microscope camera using differential interference contrast (DIC) microscopy or epifluorescence. Conidia were stained with 5μl calcofluor white (CFW) solution (Fluka) and incubated at 25°C for 30 minutes. Cell number *per* conidium was determined by visualizing septa with CFW. Appressorium-mediated penetration of onion epidermal strips was assessed using a procedure based on Chida and Sisler[79]. A conidial suspension at a concentration of 1 x 10^5^ conidia mL^-1^ was prepared and dropped onto the adaxial surface of epidermal layers taken from onion. The strips were incubated in a moist chamber at 25°C and penetration events scored 24h to 48h later by recording images with an Olympus IX81 inverted microscope system. Leaf sheath assays were carried out as previously described [10]. Glycogen staining solution contained 60 mg of KI and 10 mg of I_2_ per milliliter of distilled water. Glycogen deposits are visible immediately. For cytorrhysis assays, 10^5^ spores were allowed to form appressoria for 18h on coverslips prior the addition of external glycerol (1M or 3M). After 10 minutes in glycerol ~500 appressoria were analyzed in each biological replica; experiment was carried out by triplicate. To visualize lipid droplets, conidia were allowed to germinate in water on coverslips. After 0h, 2h, 9h and 12h water was removed and conidia directly stained with Nile red (Nile Red Oxazone (9-diethylamino-5Hbenzo[alpha]phenoxazine-5-one; Sigma). Nile red was used to 2.5 mg/ml diluted in 50mM Tris/Maleate, pH 7.5 and polyvinylpyrrolidone (PVP) (2–3% w/v). Lipid droplets begin to fluoresce within seconds. Samples were visualized under a confocal laser scanning microscope using a 561 nm excitation wave length and a long pass emission filter (592–700 nm). All images were taken using the same parameters.

### Generation of mutant strains by gene replacement

Gene deletion constructs were generated using multisite gateway technology (Invitrogen) as previously described[77,80]. *TPC1*, *CON6*, *GH18*, *PEBP2* and *NOXD* coding sequences were replaced by the hygromycin resistance cassette and *PEBP1* by the sulfonylurea resistance cassette in the gene replacement constructs. Primers for constructing entry plasmids are described in S4 Table. Fungal transformants generated by *Agrobacterium*-mediated transformation [81] were selected growing in DCM solid media supplied with 5-fluoro-2’-deoxyuridine (50μM) and 200μg/ml Hygromycin or 150μg/ml Chlorymuronethyl in the case of Δ*pebp1*. DCM is 1.7 g yeast N-base without amino acids, 1.0 g NH_4_NO_3_, 2.0 g of L-asparagine and 10 g of D-glucose. Knockout strains were confirmed by PCR or Southern blotting using radioactive probes (^32^P; primers listed in S4 Table). Sequence data and gene numbers used in this study were taken from EnsemblFungi (*Magnaporthe oryzae* MG8; http://fungi.ensembl.org/index.html).

### Generation and cellular localisation of fluorescently tagged proteins

To determine the localisation of Tpc1, live-cell imaging was performed using a *M*. *oryzae* Guy11 strain containing two constructs, histone H1 tagged with red fluorescent protein (*H1*:*RFP*; tdTomato) to visualize nuclei [82], and *TPC1*:*GFP*. For the construction of a functional *TPC1*:*GFP* gene fusion, primers were designed in order to amplify the *TPC1* (MGG_01285) promoter region and ORF from genomic DNA of *M*. *oryzae* Guy11 (S4 Table). The *TPC1_GFP_F* forward primer was designed approximately 1.3 kb upstream from the *TPC1* start codon to include a substantial component of the promoter sequence. The *TPC1_GFP_R* reverse primer spanned the stop codon and contained a complementary region to the *GFP* sequence. GFP primers were designed to amplify the 1.4 kb sGFP:TrpC construct cloned in pGEMT. Both fragments were joined together by fusion nested PCR. The amplicons were cloned into pGEMT-easy digested with *Eco*RI. The 4.3 kb *TPC1*:*GFP* fragment was gel purified and cloned into pCB1532 that had previously been digested with *Eco*RI. The pCB1532 vector contains the 2.8 kb *ILV1* gene, which encodes the acetolactate synthase-encoding allele bestowing resistance to sulfonylurea[83]. The resulting plasmid pCB1532-*TPC1*:*GFP* was used to transform protoplasts of M1422 mutant. For all rounds of PCR amplification, Phusion High-Fidelity DNA polymerase (Finnzymes, Thermo Fischer Scientific Inc.) was used, following the manufacturers’ guidelines for PCR conditions.

The *GFP*:*MoATG8*[34] and the *FIM*:*GFP* constructs were used to transform protoplasts of M1422 mutant. Protoplast generation and transformation were carried out as previously described[76]. The *GFP*:*MoATG8* and the *FIM*:*GFP* protein fusion vectors were generated using the native *M*. *oryzae MoATG8* gene (MGG_01062) and the native *M*. *oryzae* fimbrin-encoding gene (MGG_04478), respectively. Both fragments were cloned into pCB1532 vector that contains the 2.8 kb *ILV1* gene, which encodes the acetolactate synthase allele conferring sulfonylurea resistance. Transformants showing identical growth and colony morphology to the background strain were selected for further examination using epifluorescence or confocal microscopy. At least three different transformants of each were independently analysed.

The *TPC1*:*GFP* gene fusion was cloned into pCB1532 vector (SUR^R^) and used to transform protoplasts of Guy11 expressing Histone H1 fused with red fluorescent protein (*H1*:*RFP*)[33], and also introduced into isogenic Δ*pmk1*, Δ*atg1* and Δ*atg8* mutants. Transformants were selected for further examination using confocal microscopy and verified as containing a single copy of the gene fusion construct by Southern blot hybridisation. At least three different transformants of each were used in all experiments.

### RNA isolation and global gene expression profile using microarrays

Using a modified protocol of LiCl method[77], RNA was extracted from 8-day old fungal mycelia grown on cellophane placed on top of CM agar plates (S2E Fig). Two to three additional washes with phenol:chloroform were implemented to avoid RNA degradation from cellophane samples. RNA quality control was carried out with Agilent RNA 6000 Nano kit (ref. 5067–1504). Four biological replicates were independently hybridized for each transcriptomic comparison. Each of these replicates derived from three technical repetitions. Slides were Agilent Magnaporthe II Oligo Microarrays 4x44K (ref. 015060). Background correction and normalization of expression data were performed as previously described[77]. Hybridizations and statistical analysis were conducted by the Genomics Facility at the National Biotechnology Centre (Madrid, Spain). The GO term analysis was carried out with gProfiler[84]. Enriched motifs were not found when using the promoter regions of the 185 up-regulated genes. Microarray data are available in the ArrayExpress database (EMBL_EBI) under accession number E-MTAB-4127.

### Yeast-two hybrid screen

In-Fusion Cloning based on *in vitro* homologous recombination was performed to generate vectors including NoxD and Tpc1 into the pGADT7 prey vector, and Nox1, Nox2 NoxR, Pmk1 and Mst12 into the pGBKT7 bait vector. Genes were amplified from *M*. *oryzae* cDNA derived from mycelia grown on liquid CM using primers with a 15bp overhang and restriction site complementary to the target vector (S4 Table). For NoxD, a 435bp fragment was amplified, for Nox1, a 1662bp fragment was amplified, for Nox2, a 1749bp fragment was amplified, and for NoxR, a 1578bp fragment was amplified. Respective fragments were cloned into pGBKT7 and pGADT7 plasmids linearized by digestion with *Eco*RI and *Sma*I. Yeast two-hybrid assays using pGADT7 or pGBKT7 (Clontech) based constructs were performed according to the manufacturer’s instructions (MATCHMAKER Gold Yeast Two-Hybrid System).

### Imaging of fluorescent fusion proteins

For the construction of NoxD:GFP, primers were designed to amplify the ORF including 2kb upstream of the start codon, GFP and TrpC terminator with 15bp overhangs complementary to adjacent fragments (S4 Table). Fragments were ligated into pCB1532[83], which carries the sulphonyl urea resistance cassette and had been digested with *BamH*I and *Hind*III and this construct transformed into of the wild-type strain Guy11 using protoplasts[6]. The NoxD:mRFP construct was generated using multi-site gateway technology (Life Technologies) with the entry mCherry-withSTOP and destination SULPH-R3R4 vectors[77], and PCR fragments amplified from *M*. *oryzae* genomic DNA using Phusion DNA polymerase (NEB) and primers detailed in S4 Table. Appressorium development assays were performed on hydrophobic borosilicate glass coverslips (Fisher Scientific), as described previously[6]. For epifluorescence microscopy, conidia were incubated on coverslips and observed at each time point using an IX-81 inverted microscope (Olympus) and a UPlanSApo X100/1.40 oil objective. All microscopic images were analyzed using MetaMorph (Molecular Devices). Confocal imaging was performed with a Leica SP8 microscope.

### qPCR and ROS detection

To confirm microarray results, the relative abundance of gene transcripts were analysed by qPCR (S4 Table). One μg of total RNA from 8-day old fungal mycelia grown on cellophane placed on CM agar was reverse transcribed using PrimeScript RT reagent Kit (Takara). The average threshold cycle (Ct) was normalized against actin transcript and relative quantification of gene expression was calculated by the 2^ΔΔCt^ method[85]. Primer efficiency was tested using dilutions of cDNA samples. qPCR reactions were carried out with 1 μl of reverse transcribed products and fast-start DNA master SYBR green I kit (Roche Diagnostics) in a final reaction of 20 μl using the following program: one cycle of 95°C for 4 min and 40 cycles of 94°C for 30 s and 60°C for 30 s. The Ct (threshold cycle) provided a measure for the starting copy numbers of the target genes. Three technical repetitions from three independent biological experiments were used for each gene. For ROS detection in *M*. *oryzae* fungal structures, NBT staining[65] and quantification method of pixel intensities in hyphal tips[86] were carried out as previously described.

### Chromatin immunoprecipitation (ChIP) and quantitative PCR (qPCR) analysis

Two strains, the Δ*tpc1* mutant expressing *TPC1*:*GFP* and *M*. *oryzae* wild-type Guy11 strain as negative control were used for this experiment. Mycelia were grown in liquid CM at 25°C for 48 h in a shaker (120 rpm), and collected using two layers of Miracloth. Harvested mycelia were washed extensively with sterile water. To crosslink DNA and proteins, one gram of each washed mycelium was treated with 1% formaldehyde in 20 mM HEPES pH 7.4 buffer for 20 min with continuous shaking at 100 rpm. Then, 0.125 M glycine was added and incubated at room temperature for an additional 10 min to stop crosslinking. Mycelia were harvested with Miracloth, rinsed with water removing excessive water by squeezing and immediately frozen in liquid nitrogen, grinded into a fine powder and stored at -80°C until used. ChIP was conducted according to published procedures with some modifications [87]. 600 mg of each mycelium powder was used for chromatin extraction and sonication. The powder was added into 10 ml of Extraction buffer 1 (0.4 M sucrose, 10 mM Tris-HCl pH 8, 10 mM MgCl_2_, 5 mM β-mercaptoethanol/β-ME and Protease Inhibitors Complete-PIC/Roche) and mixed by vortexing. The solution was filtered through a double layer of Miracloth and centrifuged at 5000 g for 10 min at 4°C. The pellet was resuspended in 1 ml of Extraction buffer 2 (0.25 M sucrose, 10 mM Tris-HCl pH 8, 10 mM MgCl_2_, 1% Triton X-100, 5 mM β-ME and PIC) and centrifuged at 5000 g for 10 min at 4°C. The pellet was resuspended in 300 μl of Extraction buffer 3 (1.7 M sucrose, 10 mM Tris-HCl pH 8, 0.15% Triton X-100, 2 mM MgCl_2_, 5 mM β-ME and PIC) and, carefully layered on the top of additional 600 μl of extraction buffer 3. Then, samples were centrifuged at 16000 g for 60 min at 4°C. The chromatin pellet was resuspended in 300 μl of Nuclei Lysis Buffer (50 mM Tris-HCl ph 8, 10 mM EDTA, 1% SDS and PIC) and sonicated for 25 min at 4°C, operating a pattern of 30 sec ON and 30 sec OFF, at high power level in the Bioruptor Plus (Diagenode, Liege, Belgium) to obtain DNA fragments ranging from 500 to 1,000 bp. The chromatin solution was centrifuged at maximum speed for 5 min at 4°C to pellet cell debris. The supernatant was kept as chromatin solution and a small aliquot (10%) was stored as input DNA control. For each immunoprecipitation, 15 μl of Dynabeads Protein A magnetic beads (ref. 10001D, Life Technologies) was washed twice with 500 μl ChIP dilution buffer (1.1% Triton X-100, 1.2 mM EDTA, 16.7 mM Tris-HCl pH 8, 167 mM NaCl and PIC). Then, anti-GFP antibody (ref. A6455, Life Technologies) was added and incubated with gentle rotation for 1h at 4°C in 50 μl ChIP dilution buffer. Prepared anti-GFP coated beads were washed twice with 500 μl ChIP dilution buffer and resuspended in 100 μl of ChIP dilution buffer. For each immunoprecipitation, the latter and 100 μl of chromatin solution were gathered together and diluted up to 1 ml of ChIP dilution buffer. All immunoprecipitations were incubated overnight at 4°C with gentle rotation, then washed with a serie of wash buffers (2 washes with Low Salt Wash Buffer: 150 mM NaCl, 0.1% SDS, 1% Triton X-100, 2 mM EDTA, 20 mM Tris-HCl pH 8; one wash with High Salt Wash Buffer: 500 mM NaCl, 0.1% SDS, 1% Triton X-100, 2 mM EDTA, 20 mM Tris-HCl pH 8; one wash with LiCl Wash Buffer: 0.25 M LiCl, 1% NP-40, 1% sodium deoxycholate, 1 mM EDTA, 10 mM Tris-HCl pH 8, 2 washes with TE Buffer: 10 mM Tris-HCl pH 8, 1 mM EDTA). Immunoprecipitated DNAs and Input DNA control were reverse-crosslinked at 95°C for 10 min with 200 μl of 10% chelex 100 resin to remove any trace of metals. DNA samples were treated with proteinase K that was inactivated afterwards. After centrifugation, supernatants of DNA samples were stored at -20°C until used. Immunoprecipitated chromatin was diluted 10 times for qPCR analysis (primers listed in S4 Table). This was performed using a Roche LightCycler 480 machine. qPCR reactions were carried out using either 2 μl of input DNA or 2 μl of immunoprecipitated chromatin in a final reaction of 12 μl with the following program: one cycle of 95°C for 5 min and 58 cycles of 94°C for 10 s, 60°C for 10 s and 72°C for 10 s. The Ct (threshold cycle) provided a measure for the starting copy numbers of DNA. Three technical repetitions from 4 independent biological experiments were used. Ct values were used to calculate ratios evaluating the fold difference between experimental samples (GFP-tagged or untagged wild-type strains) and normalized the input. We normalized with “Fold Enrichment Method” using the untagged strain. The Wilcoxon Mann Whitney test was applied to analyze the difference between two independent groups. Statgraphics software was used to make pairwise comparisons between GFP-tagged strain and untagged wild-type strain.

### Protein purification and EMSA

*M*. *oryzae MST12* and *TPC1* cDNAs derived from mycelial RNA were cloned by PCR using a high fidelity Q5 DNA polymerase (NEB), primers (S4 Table) and the restriction enzymes BamHI-NotI and EcoRI- NotI for *MST12* and *TPC1* respectively, into a modified pET28 vector (5,667bp; Novagen). *MST12-* and *TPC1*-containing plasmids were transformed in *E*. *coli* Rosetta DE3 (Novagen) and colonies grown in LB medium containing chloramphenicol (34 μg/L) and kanamycin (50 μg/L) until reaching OD_600nm_ = 0.8. Protein expression was induced 4 hours at 28°C with 1 mM IPTG (Sigma-Aldrich). Centrifuged cell pellets (30 min at 7000g) were resuspended in lysis buffer (20 mM sodium phosphate pH 8, 300 mM NaCl and one tablet of PIC/50 ml, 1 mM PMSF and 50 μg/ml Dnase I), lysed by sonication and pelleted at 4°C and high speed (20 min at 20,000g). Recombinant proteins were purified from clear lysate by metal affinity chromatography (HisTrap HP 1 ml, #17-5247-01 GE Healthcare) in denaturing conditions using 6 M Urea and eluted with 250 mM imidazole containing buffer. Samples were desalted on PD10 column (#17085101 GE Healthcare) to remove urea and imidazole using buffer (20 mM sodium phosphate pH 8, 10% glycerol and PIC). Protein samples purity was evaluated by SDS-PAGE.

EMSA probes were generated as follows. Amplified by PCR fragments using primers listed in S4 Table were prepared using modified Biotin 3’end DNA labeling procedure (#89818 Thermo-Scientific). Briefly, each ~500pb purified PCR products was KpnI-digested, purified and labelled (5 pmol of each probe) with Biotin-11-UTP and Terminal Deoxinucleotidyl Transferase at 37°C for 1 hour. Biotinylated probes were purified by Chloroform:IAA (24:1) extraction and stored at -20°C until use. EMSA reactions (20 μl) contained 10 mM Tris HCl pH 7.5, 50 mM KCl, 16 mM DTT, 1 mM ZnCl_2_, 1 mM MgCl_2_, 1% Glycerol, 50 ng/μl Poly dI-dC (#20148E Thermo-Scientific), 10 μg BSA, Protease inhibitor complete (Roche), and 80 fmol of biotinylated probe. Before probe addition proteins (0–12 μM) were incubated in binding buffer for 10 min, then probe was added and incubated during 30 min at room temperature before loading. The EMSA gel (0.2% agarose, 5% polyacrylamide, 1% glycerol in TBE 0.5x) was run for 2h 100V in TBE 0.5x and then transferred to a Hybond-XL nylon membrane (#RPN203S GE Healthcare) at 400 mA for 1 hour. The membrane was UV crosslinked at 120mJ/cm^2^. Detection was performed with stabilized streptavidin-horseradish peroxidase conjugate (#21134 Thermo-Scientific) and enhanced chemiluminescent substrates (#32106 Thermo-Scientific) following LightShift Chemiluminescent EMSA procedure (#20148 Thermo-Scientific).

### Phylogenetic analysis of Tpc1

First, 141 *M*. *oryzae* protein sequences containing a fungal Zn(II)_2_Cys_6_ binuclear cluster domain (PF00172) were identified from the *Magnaporthe* sequence database at the Broad Institute (http://www.broadinstitute.org/annotation/fungi/magnaporthe) and the Fungal Transcription Factor Database (http://ftfd.snu.ac.kr/intro.php). HMMsearch from HMMER3[88] was used to screen the genome assembly of *M*. *oryzae* proteins with the fungal Zn_2_Cys_6_ profile hidden Markov model pHMM zn_clus_ls.hmm (PF00172.13) from Pfam database[89] (http://pfam.xfam.org/). Subsequently, gene numbers were updated using the MG8 genome version of EnsemblFungi database (http://fungi.ensembl.org/index.html). Out of these 141 sequences, only 113 had a full length zinc cluster domain, and extra six closest sequences were included to build S5 Fig. Additional Zn(II)_2_Cys_6_ proteins found in Lu et colleagues[28] were included in S2 Table. Basic Local Alignment Search Tool (BLAST) was used to find orthologous proteins of TPC1/MGG_01285 (http://blast.ncbi.nlm.nih.gov/Blast.cgi). Protein sequences were pre-aligned using HMMalign and the pHMM zn_clus_ls.hmm (S4 Fig) from Pfam. The Zn(II)_2_Cys_6_ binuclear cluster domain region was extensively manually aligned in BioEdit (http://www.mbio.ncsu.edu/BioEdit/BioEdit.html). Unambiguous aligned positions were used for the subsequent phylogenetic analyses. The maximum likelihood (ML) analyses were performed with the program PhyML version 3.0.1[90]. All trees were visualised using the program Figtree (http://tree.bio.ed.ac.uk/software/figtree/).

### Accession numbers

*M*. *oryzae* sequence data from this article can be found in the GenBank/EMBL-EBI (EnsemblFungi) databases under the following accession numbers: *TPC1* (MGG_01285), *PMK1* (MGG_09565), *MST12* (MGG_12958), *ATG1* (MGG_06393), *ATG8* (MGG_01062), *CON6* (MGG_02246), *GH18* MGG_04732, *NOXD* (MGG_09956), *PEBP1* (MGG_06800), *PEBP2* (MGG_14045), *NOXR* (MGG_05280), *NOX1* (MGG_00750), *NOX2* (MGG_06559), *FIMBRIN* (MGG_04478) *GELSOLIN* (MGG_10059), *ACTIN* (MGG_03982), *YDIU* (MGG_03159) and *SEP5* (MGG_03087).

## Supporting information

S1 Fig M1422 phenotype defects are restored by re-introduction of *TPC1*:*GFP* construct. (PDF)

S2 Fig Gene-replacement strategy to generate *TPC1* deletion mutants. (PDF)

S3 Fig Cellular localisation of fimbrin in *M*. *oryzae* wild-type and M1422 strains. (PDF)

S4 Fig Phylogenetic analysis of *M*. *oryzae* Tpc1 protein. (PDF)

S5 Fig Maximum likelihood tree of *M*. *oryzae* Zn(II)_2_Cys_6_ cluster proteins. (PDF)

S6 Fig *M*. *oryzae* Tpc1 has an orthologue in *N*. *crassa* with similar roles in polarized growth development. (PDF)

S7 Fig Gene replacement of *M*. *oryzae CON6*, *GH18*, *NOXD*, *PEBP1* and *PEBP2* genes. (PDF)

S8 Fig NoxD is not involved in *M*. *oryzae* response to stress conditions. (PDF)

S1 Table List of characterized transcriptional regulators in *M*. *oryzae*. (PDF)

S2 Table Complete list of Zn(II)_2_Cys_6_ binuclear family of transcriptional regulators in *M*. *oryzae*. (PDF)

S3 Table List of differentially expressed genes in Δ*tpc1* identified by transcriptome analysis. (PDF)

S4 Table Primers used in this study. (PDF)
