# Supplementary material for: Tpc1 is an important Zn(II)2Cys6 transcriptional regulator required for polarized growth and virulence in the rice blast fungus
Source: PLoS Pathog. 2017 Jul 24;13(7):e1006516. doi: 10.1371/journal.ppat.1006516 (PMC5542705; doi:10.1371/journal.ppat.1006516)
Supplement: S1 Fig — (PDF) [file ppat.1006516.s001.pdf]

S1 Figure

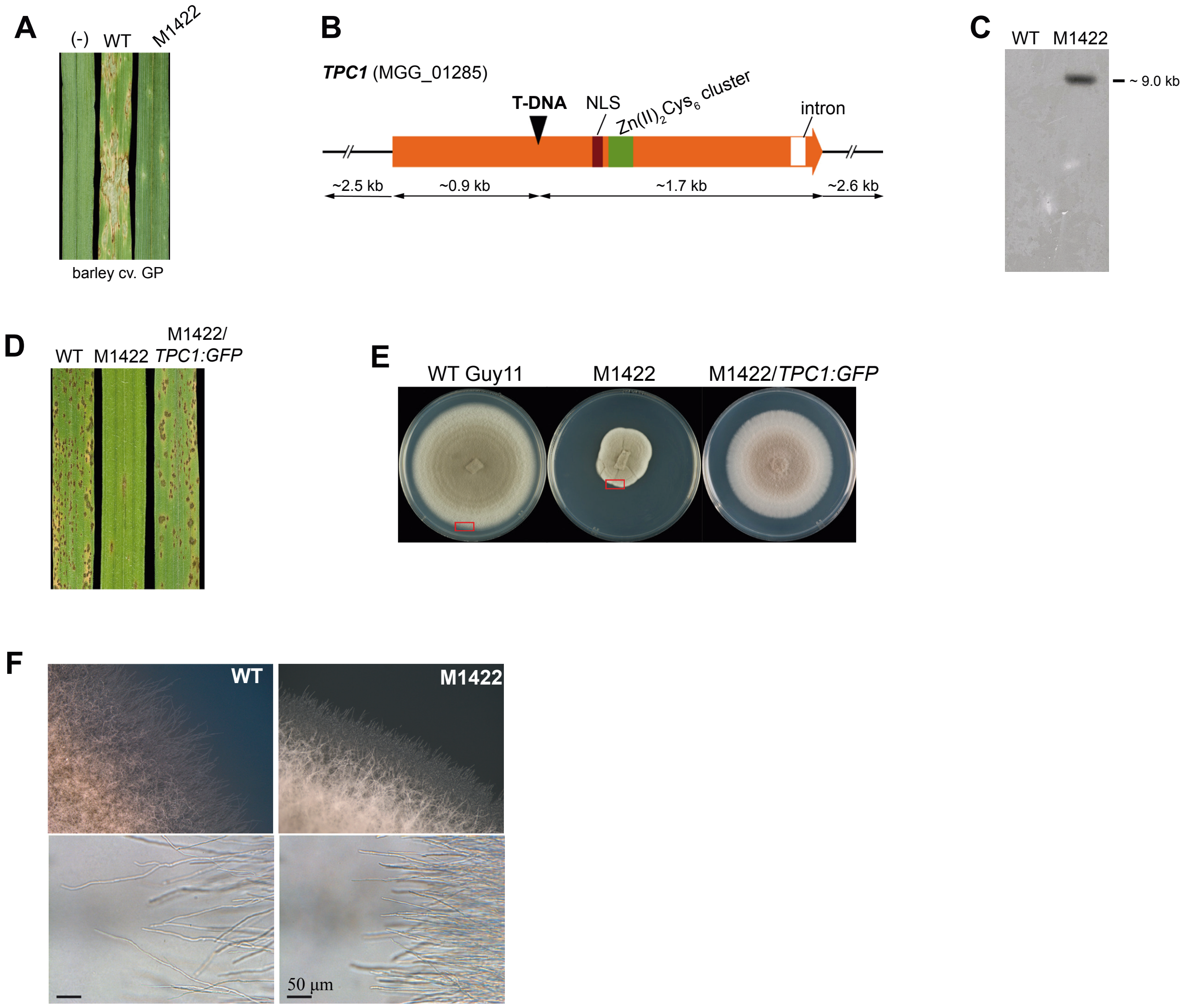

S1 Fig. M1422 phenotype defects are restored by re-introduction of *TPC1:GFP* construct. (A) M1422 is strongly impaired in its ability to infect barley leaves. **(B)** The T-DNA in M1422 is positioned 0.9 kb after the start codon of the MGG_01285 gene. This gene has been termed *TPC1* (*Transcription factor for Polarity Control-1*). Features of *TPC1*-encoding protein: NLS, nuclear localisation signal (brown box); Zn(II)$_2$Cys$_6$ binuclear cluster DNA binding domain (green box). **(C)** Southern hybridization of total genomic DNA digested with *Mfe*I and probed with hygromycin phosphotransferase. The presence of a single hybridizing restriction fragment of ~9 kb confirms a single T-DNA insertion event in M1422 genome. **(D)** Rice cv. CO39 leaves infected with a conidial suspension (10^5 conidia ml^{-1}) of Guy11, M1422 and M1422 complemented with *TPC1:GFP*. **(E)** Colonies of the wild-type (WT) strain Guy11, M1422 and M1422 complemented with *TPC1:GFP*. Photographs were taken after incubating on CM at 25°C for 10 days. **(F)** Colony edges of WT Guy11 strain show longer hyphal tips emerging from the edge of the colony compared to mutant M1422.
