# Supplementary material for: Tpc1 is an important Zn(II)2Cys6 transcriptional regulator required for polarized growth and virulence in the rice blast fungus
Source: PLoS Pathog. 2017 Jul 24;13(7):e1006516. doi: 10.1371/journal.ppat.1006516 (PMC5542705; doi:10.1371/journal.ppat.1006516)
Supplement: S2 Fig — (PDF) [file ppat.1006516.s002.pdf]

S2 Figure

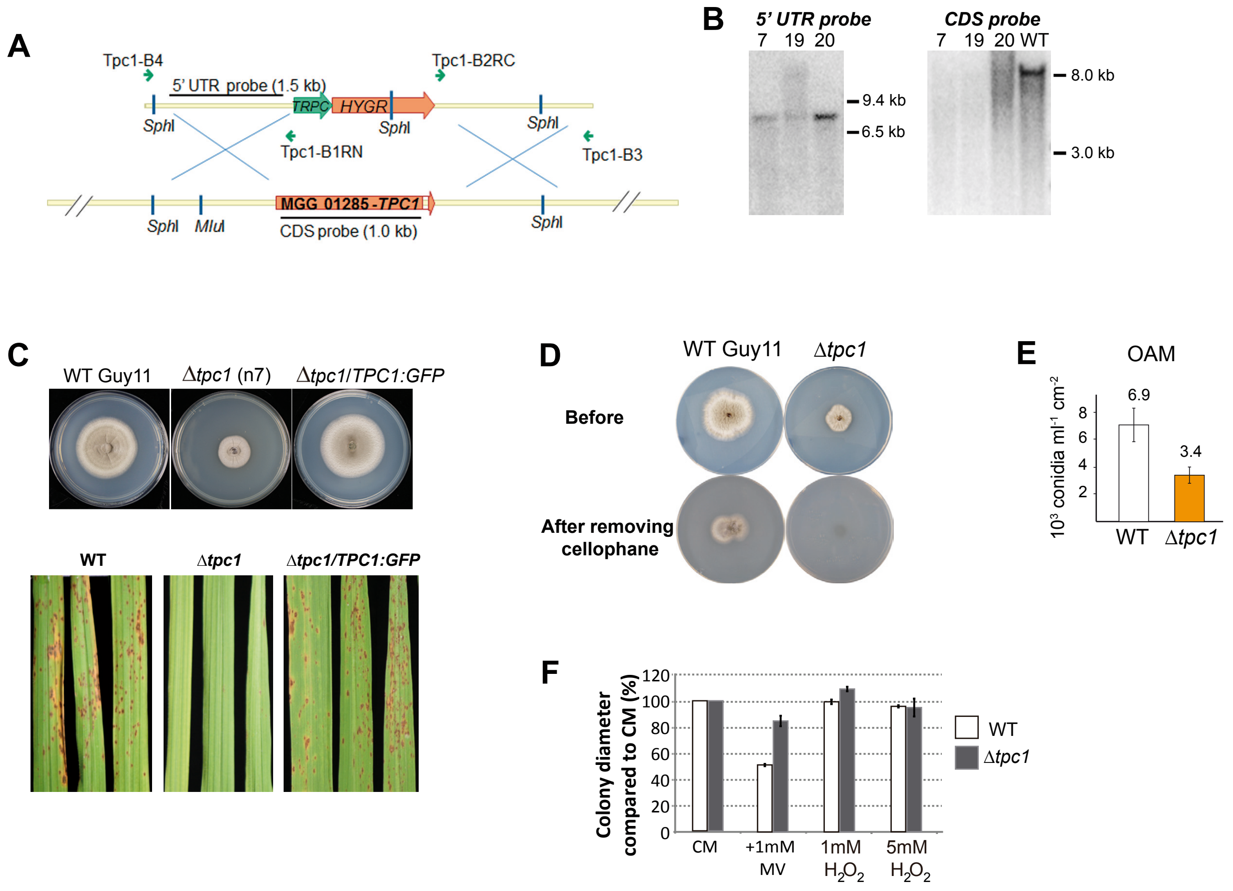

S2 Fig. Gene-replacement strategy to generate *TPC1* deletion mutants. (A) Gene replacement construct lacking the entire coding sequence (CDS) of *TPC1* was introduced into wild-type (WT) strain Guy11. **(B)** Confirmation of Δ*tpc1* mutants was carried out by Southern blotting. Total genomic DNA digested with *Mlu*I and probed with *TPC1* CDS (right panel) gave the expected band size (8.2 kb) in the WT strain and no bands in knockout strains. Similarly, total genomic DNA digested with *Sph*I and probed with *TPC1* 5'UTR (left panel) confirmed the presence of a 7.4 kb band in *TPC1* deletion mutants. **(C)** Introduction of *TPC1:GFP* fusion construct into the Δ*tpc1* mutant recovered its severe growth and pathogenicity defects. **(D)** CM plates covered with cellophane were used to grow WT and Δ*tpc1*. Eight-day old mycelia on CM plates covered with cellophane (upper plates) and six-day grown mycelia after removing the cellophane (lower plate). In contrast to the WT, Δ*tpc1* is unable to grow on CM after removing the cellophane, which suggested its inability to cross this plastic barrier. **(E)** Δ*tpc1* mycelia produces less conidia per cm^2 than WT mycelia on oat meal agar medium (OAM). Error bars represent the standard deviation of three independent experiments (n>300). **(F)** Effect of different oxidative stresses in WT and Δ*tpc1* strains. The Δ*tpc1* mutant is more resistant than WT on complete medium (CM) supplemented with 1mM methyl viologen (MV) and 1mM H_2O_2. Growth was monitored at 8 dpi. CM is taken as reference for each strain.
