# Supplementary material for: Tpc1 is an important Zn(II)2Cys6 transcriptional regulator required for polarized growth and virulence in the rice blast fungus
Source: PLoS Pathog. 2017 Jul 24;13(7):e1006516. doi: 10.1371/journal.ppat.1006516 (PMC5542705; doi:10.1371/journal.ppat.1006516)
Supplement: S3 Fig — (PDF) [file ppat.1006516.s003.pdf]

S3 Figure

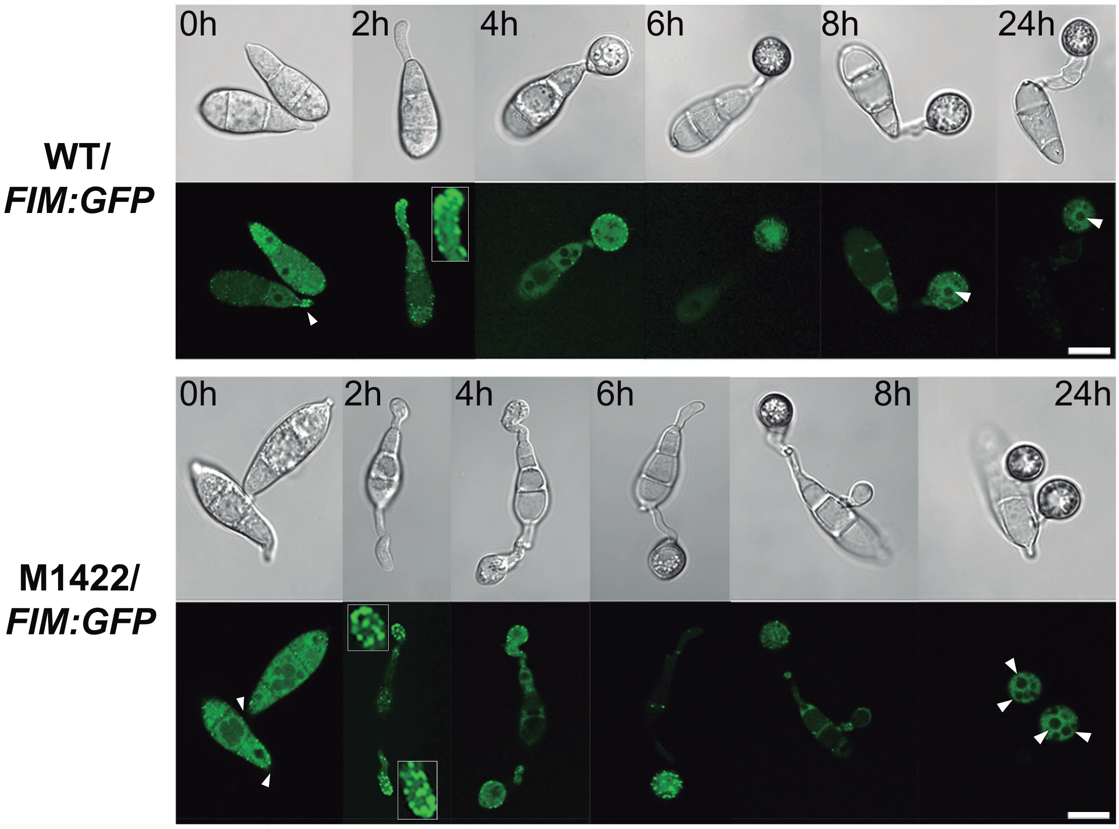

S3 Fig. Cellular localisation of fimbrin in *M. oryzae* wild-type and M1422 strains. Fimbrin is an actin-binding protein and its localization varies in the M1422 background during infection-related development compared to the wild-type strain (WT). Transformants expressing *FIM:GFP* at the times indicated. White arrowheads point germ tubes emergence at 0h and appressorial pores at 24h.
