# Supplementary material for: Tpc1 is an important Zn(II)2Cys6 transcriptional regulator required for polarized growth and virulence in the rice blast fungus
Source: PLoS Pathog. 2017 Jul 24;13(7):e1006516. doi: 10.1371/journal.ppat.1006516 (PMC5542705; doi:10.1371/journal.ppat.1006516)
Supplement: S4 Fig — (PDF) [file ppat.1006516.s004.pdf]

# S4 Figure

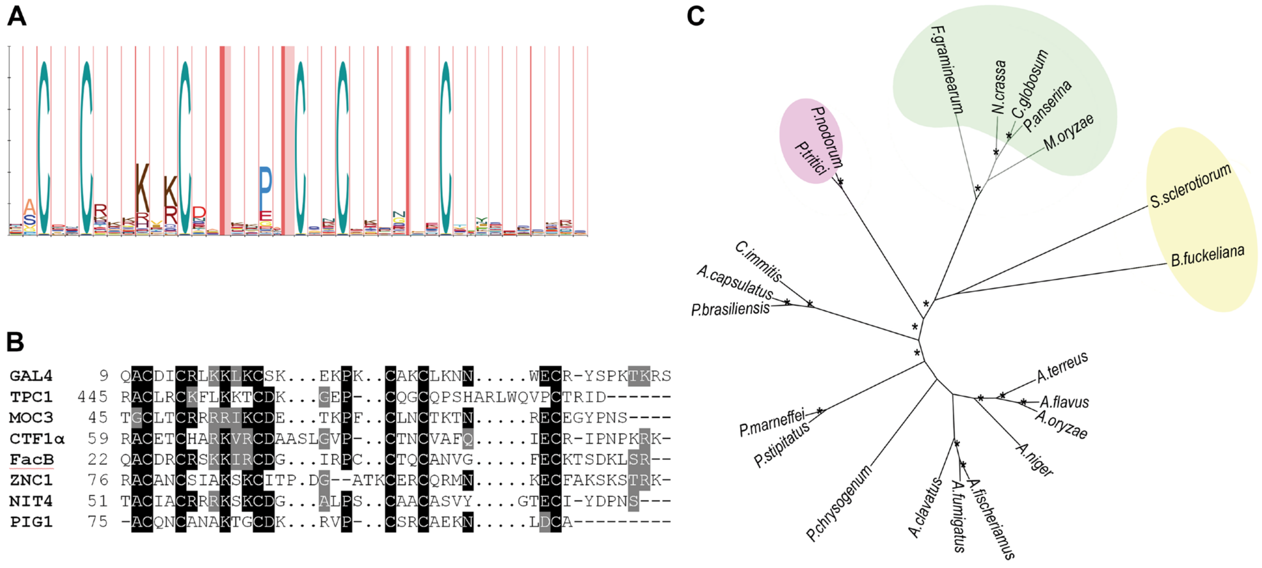


**S4 Fig. Phylogenetic analysis of *M. oryzae* Tpc1 protein. (A)** HMM pattern for Zn(II) $_2$Cys$_6$ binuclear cluster protein family. The fungal Zn(II)$_2$Cys$_6$ cluster protein family is characterised by 6 cysteine residues arranged as the motif $CX_2CX_6CX_{5\text{-}12}CX_2CX_{6\text{-}8}C$ that bind to two zinc atoms. **(B)** Homology of the Zn(II) $_2$Cys$_6$ binuclear cluster motif. Alignment constructed using HMME R3 and fungal Zn $_2$Cys$_6$ binuclear cluster domain Hidden Markov Model (PF00172.13) with Zn $_2$Cys$_6$ regions of the following fungal transcription factors: *Magnaporthe oryzae* TPC1 (MGG_01285) and PIG1 (MGG_07215); *Saccharomyces cerevisiae* GAL4 (YPL248C); *Aspergillus nidulans* FacB (ANID_00689); *Neurospora crassa* NIT4 (NCU08294); *Schizosaccharomyces pombe* MOC3 (SPAC821.07c) and *Nectria hematococca* CTF1α (AAA96824.1). Conservative aminoacid substitutions are indicated in gray boxes and identical aminoacids in black bo xes. **(C)** Maximum likelihood tree of *M. oryzae* Tpc1 protein (MGG_01285) with its closest fungal orthologues. Orthologous proteins of MGG_01285 were identified using BLAST and aligned manually in BioEdit program. This unrooted phylogenetic tree was construct ed using an alignment of 22 fungal sequences from different fungal species: *Ajellomyces capsulatus* (EH07230.1), *A. clavatus* (XP_001271908.1), *A. fischerianus* (XP_001266982.1), *A. flavus* (EED55362.1), *A. fumigatus* (XP_751792.1), *A. niger* (An04g06640), *A. or yzae* (XP_001820273.1), *A. terreus* (XP_001210781.1), *Botryotinia fuckeliana* (BC1G_06121), *Chaetomium globosum* (CHGG_09110), *Coccidioides immitis* (CIMG_00566), *Fusarium graminearum* (FG08769.1), *N. crassa* (NCU05996), *M. oryzae* (MGG_01285), *Podospora anserina* (XP_001906056.1), *Paracoccidioides brasiliensis* (EEH45457.1), *Penicillium chrysogenum* (Pc22g12400), *P. marneffei* (XP_002151174.1), *Phaeosphaeria nodorum* (SNOG_06665), *P. stipitatus* (EED24478.1), *Pyrenophora tritici* (XP_001931826.1) and *Sclerotinia sclerotiorum* (SS1G_00170). Fungal species within a pink, green and yellow balloon belong to the Dothideomycetes, Sordariomycetes and Leotiomycetes classes, respectively. The other species belong to Eurotiomycetes ( *Ajellomyces*, *Aspergillus, Paracoccidioides* and *Penicillium* species) and Euascomycetes ( *Coccidioides*) classes. Asterisks in the clades denote high likelihood ratio test (LRT) support values (*LRT > 80%).
