# Supplementary material for: Tpc1 is an important Zn(II)2Cys6 transcriptional regulator required for polarized growth and virulence in the rice blast fungus
Source: PLoS Pathog. 2017 Jul 24;13(7):e1006516. doi: 10.1371/journal.ppat.1006516 (PMC5542705; doi:10.1371/journal.ppat.1006516)
Supplement: S5 Fig — (PDF) [file ppat.1006516.s005.pdf]

S5 Figure

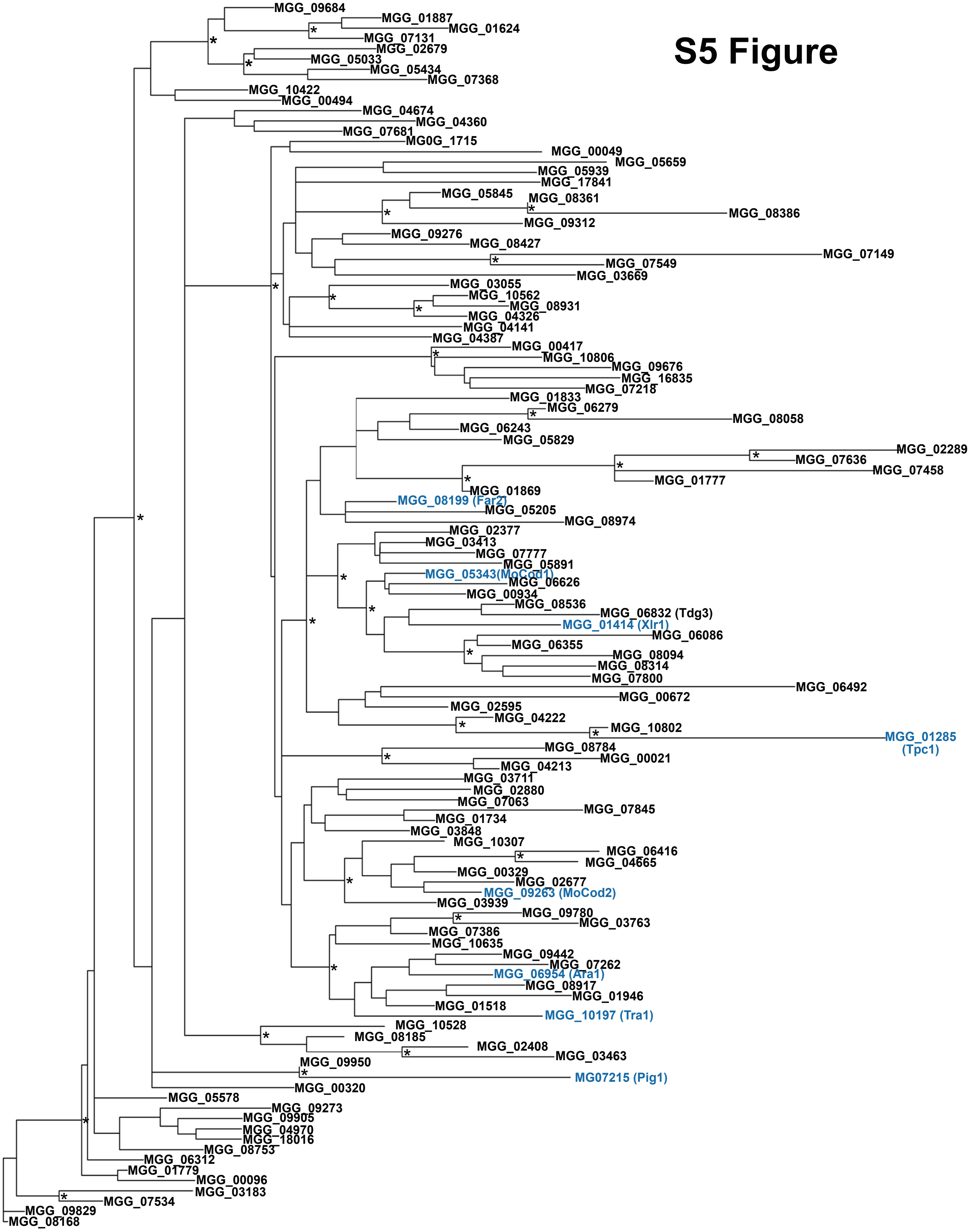

S5 Fig. Maximum likelihood tree of *M. oryzae* Zn(II)$_2$Cys$_6$ cluster proteins. Proteins containing Zn(II)$_2$Cys$_6$ domain were identified in the *Magnaporthe* database using PFAM pHMM zn_clus_ls.hmm. The unrooted phylogenetic tree was constructed using an alignment of 119 zinc cluster *M. oryzae* sequences. Asterisks in the clades denote high LRT support values (* LRT > 80%).
