# Supplementary material for: Tpc1 is an important Zn(II)2Cys6 transcriptional regulator required for polarized growth and virulence in the rice blast fungus
Source: PLoS Pathog. 2017 Jul 24;13(7):e1006516. doi: 10.1371/journal.ppat.1006516 (PMC5542705; doi:10.1371/journal.ppat.1006516)
Supplement: S6 Fig — (PDF) [file ppat.1006516.s006.pdf]

S6 Figure

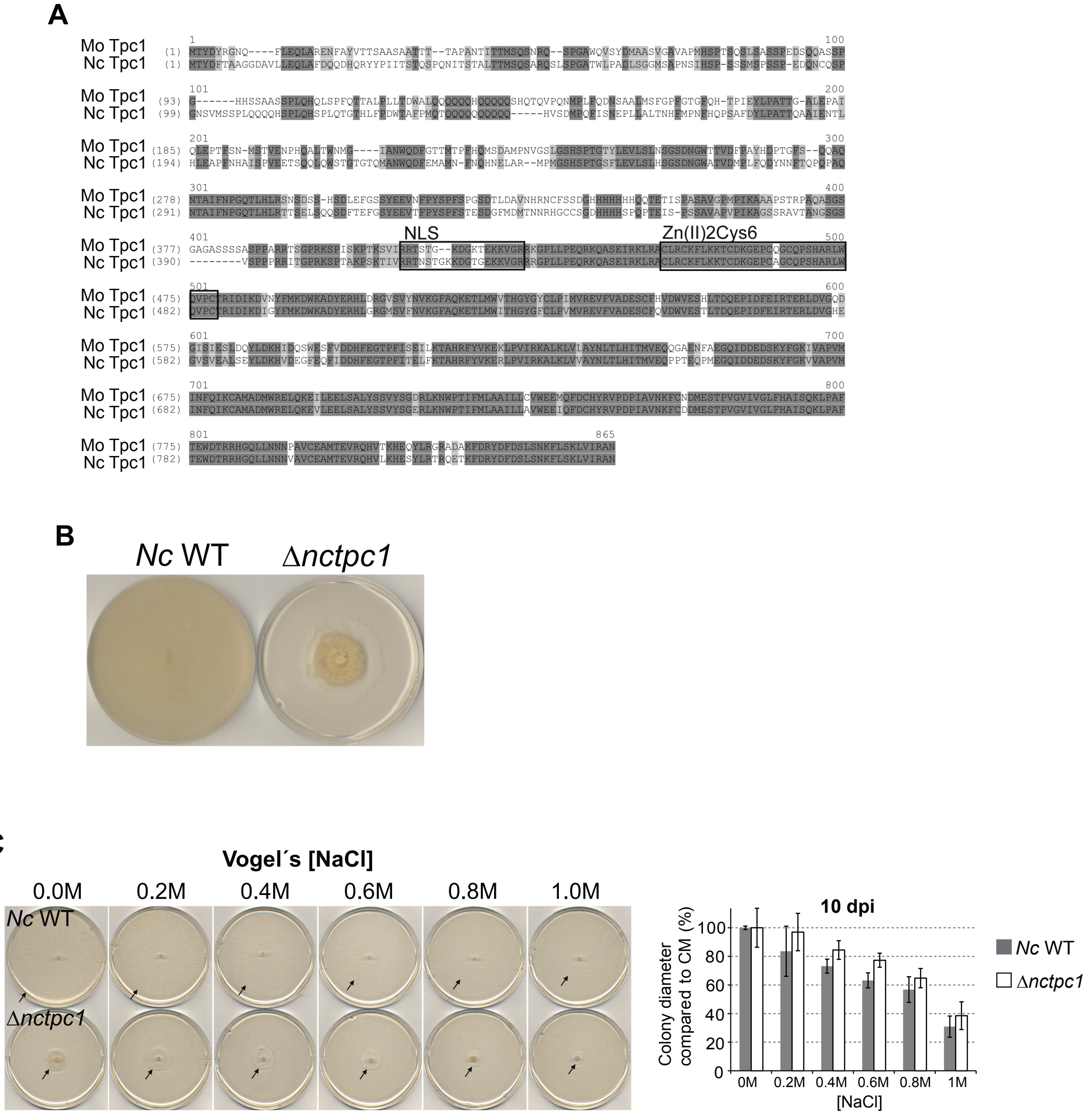

S6 Fig. *M. oryzae* Tpc1 has an orthologue in *N. crassa* with similar roles in polarized growth development. **(A)** Alignment of *M. oryzae* Tpc1 (MGG_01285) with *N. crassa* Tpc1 (NCU05996). Amino acid residues of the nuclear localisation signal (NLS) and Zn(II)$_2$Cys$_6$ cluster domain are within boxes. The N-terminus of these proteins presents a lower degree of similarity (46%), whereas the C-terminus (between 423 - 878 amino acid positions in the alignment) is 86% similar. This region included the putative nuclear localisation signal (NLS) and the Zn(II)$_2$Cys$_6$ binuclear cluster DNA binding domain. **(B)** Vegetative growth and colony morphology of *NcTPC1* deletion mutant. The *NcTPC1* KO mutant colony was severely reduced in vegetative growth and the branching of the vegetative hyphae was different to that observed for the isogenic *N. crassa* wild-type (WT) strain. **(C)** Colony images of Vogel's medium plates with increased concentrations of sodium chloride (NaCl). Colony edge is indicated with a black arrow. Diameter of WT and *NcTPC1* KO colonies reveals the increased tolerance to NaCl. Error bars represent the standard deviation of three independent biological replicas.
