# Supplementary material for: Tpc1 is an important Zn(II)2Cys6 transcriptional regulator required for polarized growth and virulence in the rice blast fungus
Source: PLoS Pathog. 2017 Jul 24;13(7):e1006516. doi: 10.1371/journal.ppat.1006516 (PMC5542705; doi:10.1371/journal.ppat.1006516)
Supplement: S7 Fig — (PDF) [file ppat.1006516.s007.pdf]

S7 Figure

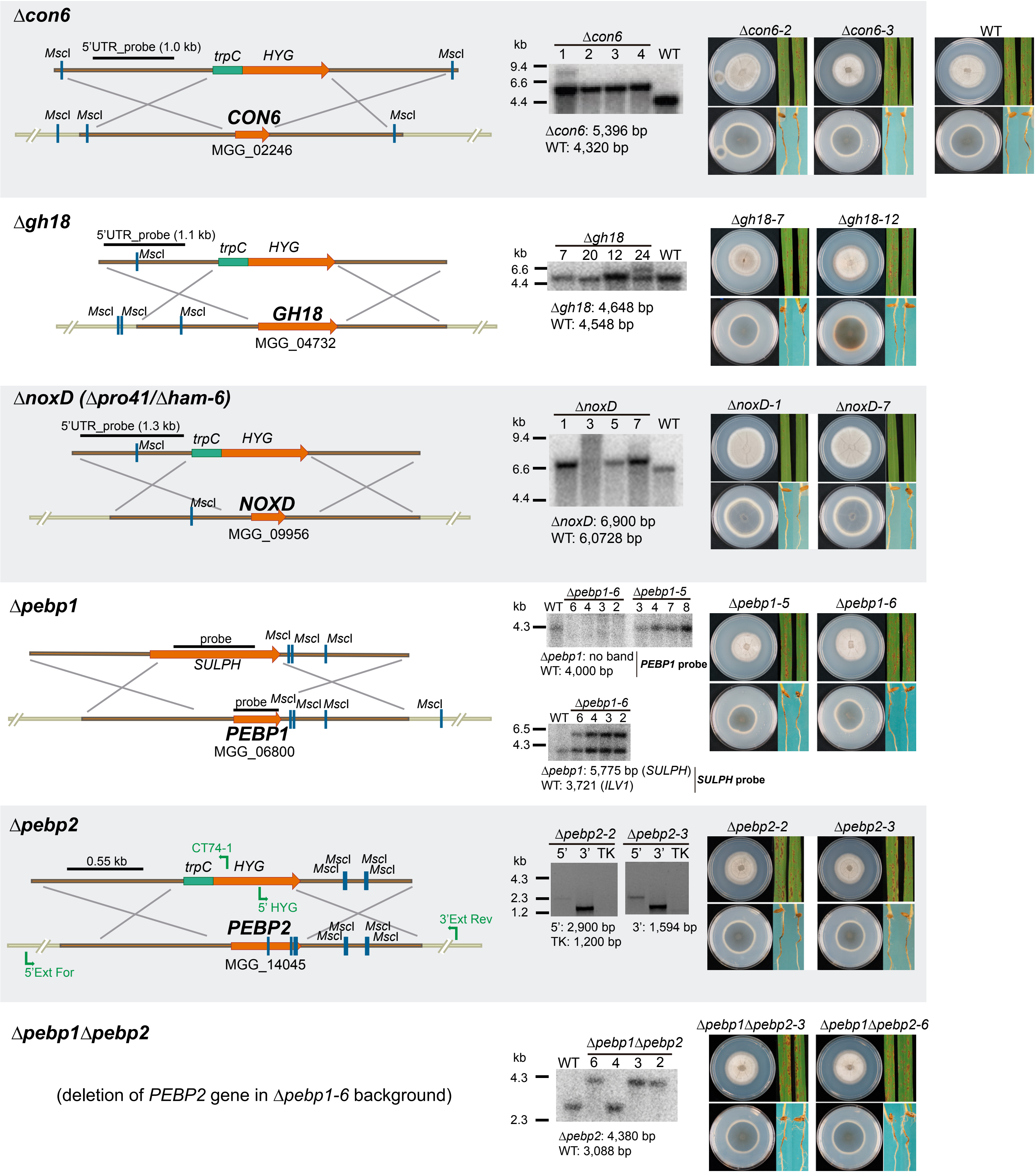

S7 Fig. Gene replacement of *M. oryzae CON6*, *GH18*, *NOXD*, *PEBP1* and *PEBP2* genes. Left panels, schematic diagram showing targeted gene deletion strategy. Middle panels, confirmation of gene replacements by Southern blotting or PCR. Right panels, growth of two deletion mutants on CM and infections tests in rice. Δ*con6*, Δ*gh18*, Δ*noxD*, Δ*pebp1* and Δ*pebp1*Δ*pebp2* were confirmed by Southern using genomic DNA digested with *Msc*I. Only Δ*pebp2* was confirmed by PCR with primers indicated in the diagram. Expected bands sizes are shown. Primers HSVTK and 3'Andy-T3 were used to detect presence of the timidine kinase gene (lane TK); lack of a PCR band indicates absence of ectopic insertions.
