# Supplementary material for: Tpc1 is an important Zn(II)2Cys6 transcriptional regulator required for polarized growth and virulence in the rice blast fungus
Source: PLoS Pathog. 2017 Jul 24;13(7):e1006516. doi: 10.1371/journal.ppat.1006516 (PMC5542705; doi:10.1371/journal.ppat.1006516)
Supplement: S8 Fig — (PDF) [file ppat.1006516.s008.pdf]

S8 Figure

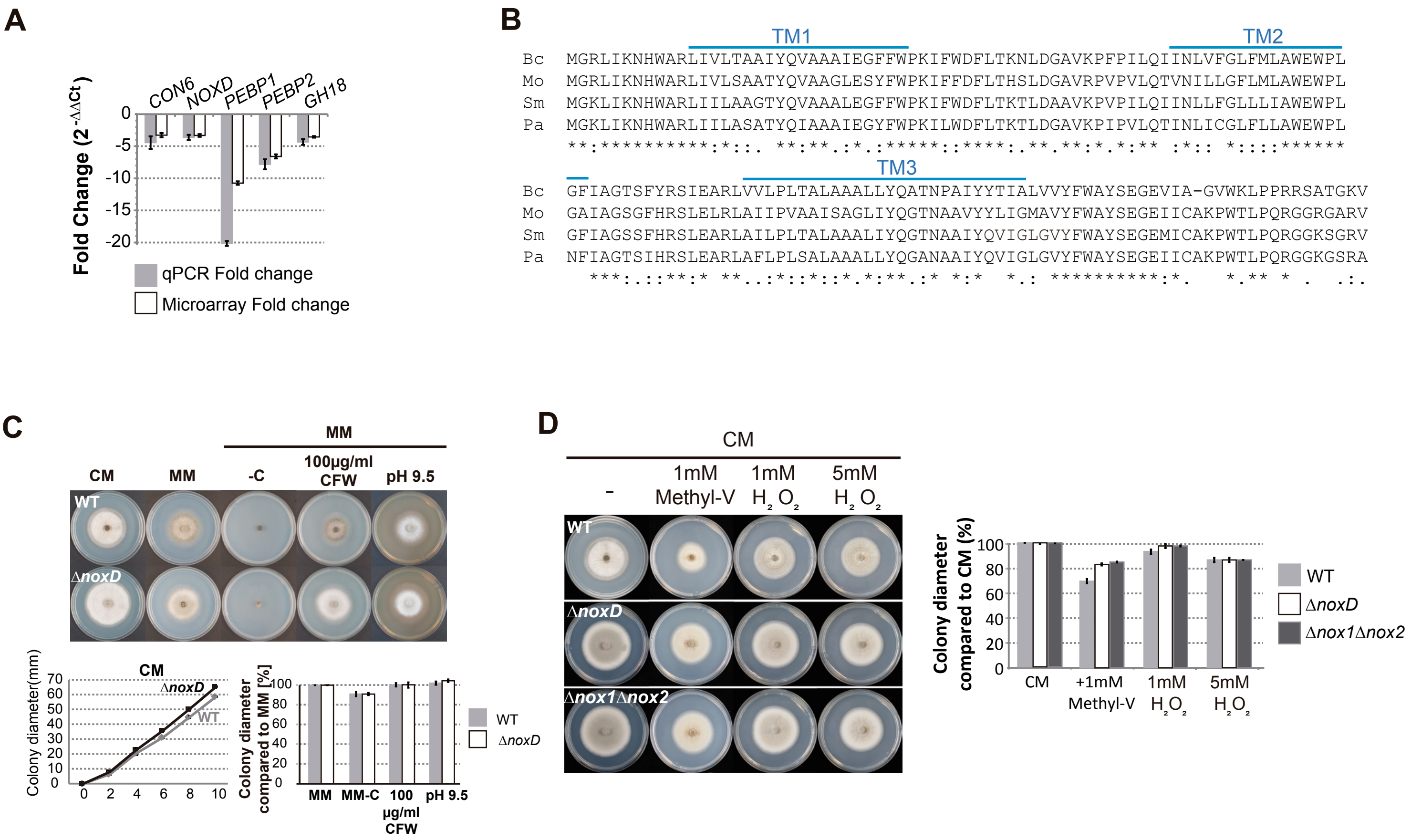

S8 Fig. NoxD is not involved in *M. oryzae* response to stress conditions. (A) Comparison of expression levels of five Tpc1-dependent genes. qPCR experiments confirmed microarray expression changes for selected genes. qPCR data were normalized against actin and wild type (WT) expression of the gene. **(B)** Fungal NoxD proteins were aligned using CLUSTAL Omega (EMBL-EBI server). Bc, *Botrytis cinerea* (EMR81583); Pa, *Podospora anserina* (CDP23073); Sm, *Sordaria macrospora* (CAL68654); Mo, *Magnaporthe oryzae* (MGG_09956.8). TM, transmembrane regions predicted using *M. oryzae* NoxD and TMHMM Server v. 2.0 (http://www.cbs.dtu.dk/services/TMHMM/). TargetP 1.1 localizes *M. oryzae* NoxD in the secretory pathway although lacks a clear signal peptide prediction. **(C)** Growth tests on CM and MM. The Δ*noxD* mutant grows slightly faster on CM while no differential growth of Δ*noxD* is observed upon exposure to carbon starvation, calcofluor white and pH 9.5. **(D)** Effect of different oxidative stresses in WT, Δ*noxD* and Δ*nox1*Δ*nox2* strains. Both mutants were more resistant than WT on complete medium (CM) supplemented with 1mM Methyl-V and 1mM H_2O_2 but not on CM with 5 mM H_2O_2. Growth was monitored at 8 dpi. CM is taken as reference for each strain.
