# Supplementary material for: Tpc1 is an important Zn(II)2Cys6 transcriptional regulator required for polarized growth and virulence in the rice blast fungus
Source: PLoS Pathog. 2017 Jul 24;13(7):e1006516. doi: 10.1371/journal.ppat.1006516 (PMC5542705; doi:10.1371/journal.ppat.1006516)
Supplement: S1 Table — (PDF) [file ppat.1006516.s009.pdf]

S1 Table. List of characterized transcriptional regulators in *M. oryzae.*

Protein name	Gene locus	aa	Function	DNA-binding domain	References
MSTU1/MoSTU1	MGG_00692.8	618	orthologue of *Aspergillus nidulans* StuA; involved in conidiation, growth, appressorium development and pathogenicity (mobilization of lipids and glycogen required for appressorial turgor generation).	APSES	[1]
MoSwi6	MGG_09869.8	895	homologue of *S. cerevisiae* Swi6; a downstream effector of MAPK Slt2 signalling; required for hyphal and conidial morphogenesis, appressorial function and pathogenicity. Interacts *in vivo* and *in vitro* with *M. oryzae* MAPK Mps1.	APSES	[2]
NUT1	MGG_02755.8	956	orthologue of *Neurospora crassa* NIT-2 and *A. nidulans* AREA; fully complements Δ*area* mutant of *A. nidulans*; required for expression of nitrogen-regulated genes; on leaves, infection efficiency of Δ*nut1* is not affected although Δ*nut1* produces smaller lesions; on roots Δ*nut1*is non pathogenic [3,4].	GATA Zn-F	[5]
MoWC-1*	MGG_03538.8	1101	homologue of *N. crassa* white collar-1; involved in light regulation	GATA Zn-F	[6]
MoMCM1	MGG_02773.8	224	Mst12-interacting protein and orthologous to yeast Mcm1; required for male fertility, microconidium production and virulence.	MADS-box	[7]
MIG1	MGG_01204.8	702	yeast RLM1 homologue; nuclear localised; interacts with MAPK MPS1 in Y2H; Δ*mig1* has normal growth rate, forms normal appressoria, penetrates developing primary infectious hyphae but it is nonpathogenic and fails to infect leaves through wounds.	MADS-box	[8]
COS1 =CON1	MGG_03977.8	491	Conidiophore stalk-less1; involved in conidiation and mycelial infection.	C2H2 Zn-F	[9,10]
MoCrz1*	MGG_05133.8	726	calcineurin-responsive transcription factor; involved in fungal growth and pathogenicity. MoCrz1 regulates expression of genes implicated in calcium signaling, small molecule transport, ion homeostasis, cell wall synthesis/maintenance, and fungal virulence.	C2H2 Zn-F	[11,12,13]
CON7*	MGG_05287.8	412	nuclear localised protein; involved in appressorium formation, proper cell wall formation, and *in planta* growth. *CON7* gene was missannotated and consequently not present in the Fungal Transcription Factor Database.	C2H2 Zn-F	[14]
TDG2/MoMsn2	MGG_00501.8	548	orthologue of AAB04132 *Nectria haematococca*; regulated by Tra1; necessary for adhesion, germination, aerial hyphal growth, conidial production, and plant infection.	C2H2 Zn-F	[15,16]
MoCDTF1	MGG_11346.8	1456	no homologue in yeast; nuclear localised;regulated by cAMP/PKA; required for growth, pigmentation, conidia and conidiophore formation, sexual development, appressorium formation from mycelium, and pathogenicity.	C2H2 Zn-F-like	[17]
PIG1	MGG_07215.8	973	required for transcription of melanin biosynthesis genes; developmentally regulated.	C2H2 Zn-F, Zn2Cys6	[18]
**TPC1***	MGG_01285.8	839	required for polarised growth, cell patterning and virulence.	Zn2Cys6	this study
TDG3	MGG_06832.8	639	orthologue of Yrm1p Q12340 *S. cerevisiae*; regulated by Tra1; no phenotype found.	Zn2Cys6	[15]
Tra1*	MGG_10197.8	726	regulated by Con7; involved in attachment, germination, appressorium formation and virulence; interacts with MoOsm1 kinase *in vivo*	Zn2Cys6; Myb	[15]
Far1; Far2	MGG_01836.8; MGG_08199.8	957; 1009	involved in growth on long chain fatty acid, acetate and short chain fatty acids; required for differential expression of genes involved in fatty acid b-oxidation, acetyl-CoA translocation, peroxisomal biogenesis, and the glyoxylate cycle in response to the presence of lipids	Zn2Cys6	[19]

Gpf1*	MGG_17841.8	700	necessary for growth, conidial germination, appressorium formation, stress response and plant infection	Zn2Cys6	[20]
Cnf2*	MGG_15023.8	963	involved in plant infection and stress response	Zn2Cys6	[20]
MoCod1	MGG_05343.8	753	expression was notably induced during conidiation; necessary for conidiation and pathogenicity due to defects in appressorium formation and invasive growth	Zn2Cys6	[21]
MoCod2	MGG_09263.8	1226	expression was notably induced during conidiation; critical regulator in conidiation and pathogenicity, not required for conidial germination and appressorium formation.	Zn2Cys6	[21]
Xlr1	MGG_01414.8	1009	XlnR homologue; involved in the transcriptional control of the pentose catabolic pathway, but not hemi-cellulolytic enzymes; *M. oryzae* Xlr1 regulates growth on D-xylose and xylan, but not on L-arabinose and arabinan.	Zn2Cys6; Myb	[22]
Ara1	MGG_06954.8	707	Transcriptional regulation of L-arabinose release and catabolism; functional analogm of AraR from *A. niger*	Zn2Cys6	[23]
MoMyb1	MGG_06898.8	395	involved in vegetative growth, cell wall biogenesis, conidiation and conidiophore development; mutant is pathogenic on leaves but not on roots	Myb	[24]
HTF1= MoHOX2	MGG_00184.8	692	involved in conidiogenesis and regulation of the expression of conidiation-related genes	Homeobox	[25,26]
HTF2= MoHOX3	MGG_01730.8	1185	∆Mohox3 has no defects in growth, conidiation, conidium size, conidial germination, appressorium formation, and pathogenicity.	C2H2 Zn-F, Homeobox	[25,26]
MST12=MoHOX8	MGG_12958.8	715	required for penetration and invasive growth; SteA	C2H2 Zn-F, Homeobox	[27,28]
HTF3= MoHOX1	MGG_04853.8	625	required for hyphal growth. The ∆Mohox1 mutant presents increase melanin pigmentation compared to wild type.	Homeobox	[25,26]
HTF4= MoHOX4	MGG_06285.8	977	involved in conidial size and hyphal growth.	Homeobox	[25,26]
HTF5= MoHOX5	MGG_07437.8	714	∆Mohox5 has no defects in growth, conidiation, conidium size, conidial germination, appressorium formation, and pathogenicity.	Homeobox	[25,26]
HTF6= MoHOX6	MGG_11712.8	685	involved in conidial size and hyphal growth.	Homeobox	[25,26]
PTH12=MoHOX7	MGG_12865.8	470	essential for appressorium formation.	Homeobox	[25,26,29]
MobZIP13/MoHAPX	MGG_05959.8	639	hap4 domain , orthologue of *A. nidulans* HapX; involved in iron homeostasis and infection	bZIP	[30]
MoATF1	MGG_08212.8	526	homologue of *S. pombe* ATF/CREB; involved in regulation of oxidative stress response. The mutant is severely impaired in enzymatic activity of extracellular laccases and peroxidases.	bZIP	[31]
MoAP1*	MGG_12814.8	576	orhtologue of *S. cerevisiae* Yap1; involved in oxidative stress response and pathogenicity; MoAP1 localises in the nucleus upon exposure to H_2O_2.	bZIP	[32]
MoHac1	MGG_09010.8	[illegible]31/556	involved in the endoplasmic reticulum stress response through a conserved unfolded protein response pathway; two alternatively spliced transcripts.	bZIP	[33]

MobZIP22/MoMETR	MGG_14561.8	302	orthologue of AnMetR (48%) and NcCyc3 (51%); involved in amino acid metabolism, sulfate assimilation, growth and differentiation, and plant infection	bZIP	[30,33]
MoBzip10	MGG_06131.8	240/324	required for appressorium function and invasive hyphal growth.	bZIP	[33]
MoBzip5	MGG_03288	616	required for appressorium formation	bZIP	[33]
MoMeaB	MGG_05306	414	involved in nutrient uptake and nitrogen utilization.	bZIP	[33]
**Other transcriptional regulators**					
MoSOM1*	MGG_04708.8	737/762	weak homology with *S. cerevisiae* Flo8 (e-04); interacts with MoSTU1 and MoCDTF1 in yeast two hibrid; nuclear localised; regulated by cAMP/PKA; five spliced variants; complements *flo8* yeast mutant; required for growth, pigmentation, conidia and conidiophore formation, sexual development, appressorium formation from mycelium, and pathogenicity.	LisH (e0.08)	[17]
MoSFL1	MGG_06971.8	600	Phosphorylated *in vitro* by PMK1; it complements the flocculation defects of the yeast Δsfl1 mutant; required for invasive growth in penetration assays with rice leaf sheaths; transcription repression domain.	HSF-like domain	[34]
MoLDB1	MGG_01057.6	806	necessary for vegetative growth, infection-related morphogenesis and pathogenicity	LIM	[35]
MoTup1	MGG_08829.8	607	homologue of *S. cerevisiae* Tup1 transcriptional repressor; involved in growth, conidiogenesis and plant infection.	Tup1 domain	[36]
Mnh6	MG04489.8	101	non-histone protein involved in mycelial growth, conidiation, appressorium development, plant penetration, and infectious growth in host cells.	HMG1 box	[37]
COM1	MGG_01215.8	774	putative transcriptional regulator present in ascomycetes; involved in conidial development and invasive growth.	RNA Pol II elongation factor domain	[4,38]
MoATH10	MGG_05185.8	562	involved in pigmentation, conidiation and virulence; the mutant shows defects in invasive growth inside plant cells, but not in appressorium-mediated penetration	AT-hook	[39]
MoGti1*	MGG_08850.8	464	necessary for conidiogenesis, appressorial formation, invasive hyphae growth and pathogenicity; regulated by MoAP1; nuclear localisation regulated by Pmk1; involved in supression of plant defense responses	Gti1/Pac2	[40]
MoPac2*	MGG_06564.8	463	regulated by MoAP1; mainly cytoplasmic protein; required for conidiogenesis and full pathogenicity	Gti1/Pac2	[40]
Nmr1-3	MGG_00156; MGG_02860; MGG_09705	321; 317; 321	transcriptional inhibitors; regulate carbon catabolite repression in *M. oryzae*	NmrA-like	[41,42]

ACR1	MGG_09847.8	862	homologue to medA (a developmental regulator of *A. nidulans* conidiation); involved in conidiophore architecture and pathogenicity.	unknown domain	[43,44]

***:available transcriptome and/or proteome data**

REFERENCES (S1 Table)

1. Nishimura M, Fukada J, Moriwaki A, Fujikawa T, Ohashi M, et al. (2009) Mstu1, an APSES Transcription Factor, Is Required for Appressorium-Mediated Infection in *Magnaporthe grisea*. Bioscience Biotechnology and Biochemistry 73: 1779-1786.
2. Qi ZQ, Wang Q, Dou XY, Wang W, Zhao Q, et al. (2012) MoSwi6, an APSES family transcription factor, interacts with MoMps1 and is required for hyphal and conidial morphogenesis, appressorial function and pathogenicity of *Magnaporthe oryzae*. Molecular Plant Pathology 13: 677-689.
3. Dufresne M, Osbourn AE (2001) Definition of tissue-specific and general requirements for plant infection in a phytopathogenic fungus. Molecular Plant-Microbe Interactions 14: 300-307.
4. Tucker SL, Besi MI, Galhano R, Franceschetti M, Goetz S, et al. (2010) Common Genetic Pathways Regulate Organ-Specific Infection-Related Development in the Rice Blast Fungus. Plant Cell 22: 953-972.
5. Froeliger EH, Carpenter BE (1996) *NUT1*, a major nitrogen regulatory gene in *Magnaporthe grisea*, is dispensable for pathogenicity. Molecular General Genetics 251: 647-656.
6. Lee K, Singh P, Chung WC, Ash J, Kim TS, et al. (2006) Light regulation of asexual development in the rice blast fungus, *Magnaporthe oryzae*. Fungal Genetics and Biology 43: 694-706.
7. Zhou X, Liu W, Wang C, Xu Q, Wang Y, et al. (2011) A MADS-box transcription factor MoMcm1 is required for male fertility, microconidium production and virulence in *Magnaporthe oryzae*. Molecular Microbiology 80: 33-53.
8. Mehrabi R, Ding S, Xu JR (2008) MADS-box transcription factor Mig1 is required for infectious growth in *Magnaporthe grisea*. Eukaryotic Cell 7: 791-799.
9. Zhou ZZ, Li GH, Lin CH, He CZ (2009) Conidiophore Stalk-less1 Encodes a Putative Zinc-Finger Protein Involved in the Early Stage of Conidiation and Mycelial Infection in *Magnaporthe oryzae*. Molecular Plant-Microbe Interactions 22: 402-410.
10. Li X, Han X, Liu Z, He C (2013) The function and properties of the transcriptional regulator COS1 in *Magnaporthe oryzae*. Fungal Biology 117: 239-249.
11. Choi J, Kim Y, Kim S, Park J, Lee YH (2009) *MoCRZ1*, a gene encoding a calcineurin-responsive transcription factor, regulates fungal growth and pathogenicity of *Magnaporthe oryzae*. Fungal Genetics and Biology 46: 243-254.
12. Zhang H, Zhao Q, Liu K, Zhang Z, Wang Y, et al. (2009) MgCRZ1, a transcription factor of *Magnaporthe grisea*, controls growth, development and is involved in full virulence. FEMS Microbiology Letters 293: 160-169.
13. Kim S, Hu JN, Oh Y, Park J, Choi J, et al. (2010) Combining ChIP-chip and Expression Profiling to Model the MoCRZ1 Mediated Circuit for Ca2+/Calcineurin Signaling in the Rice Blast Fungus. PLoS Pathogens 6: e1000909.
14. Odenbach D, Breth B, Thines E, Weber RW, Anke H, et al. (2007) The transcription factor Con7p is a central regulator of infection-related morphogenesis in the rice blast fungus *Magnaporthe grisea*. Molecular Microbiology 64: 293-307.
15. Breth B, Odenbach D, Yemelin A, Schlinck N, Schroder M, et al. (2013) The role of the Tra1p transcription factor of *Magnaporthe oryzae* in spore adhesion and pathogenic development. Fungal Genetics and Biology 57: 11-22.
16. Zhang H, Zhao Q, Guo X, Guo M, Qi Z, et al. (2014) Pleiotropic function of the putative zinc-finger protein MoMsn2 in *Magnaporthe oryzae*. Molecular Plant-Microbe Interactions 27: 446-460.
17. Yan X, Li Y, Yue X, Wang C, Que Y, et al. (2011) Two Novel Transcriptional Regulators Are Essential for Infection-related Morphogenesis and Pathogenicity of the Rice Blast Fungus *Magnaporthe oryzae*. PLoS Pathog 7: e1002385.
18. Tsuji G, Kenmochi Y, Takano Y, Sweigard J, Farrall L, et al. (2000) Novel fungal transcriptional activators, Cmr1p of *Colletotrichum lagenarium* and Pig1p of *Magnaporthe grisea*, contain Cys_2His_2 zinc finger and $Zn(II)_2Cys_6$ binuclear cluster DNA-binding motifs and regulate transcription of melanin biosynthesis genes in a developmentally specific manner. Molecular Microbiology 38: 940-954.
19. bin Yusof MT, Kershaw MJ, Soanes DM, Talbot NJ (2014) FAR1 and FAR2 regulate the expression of genes associated with lipid metabolism in the rice blast fungus *Magnaporthe oryzae*. PLoS ONE 9: e99760.
20. Lu J, Cao H, Zhang L, Huang P, Lin F (2014) Systematic analysis of Zn2Cys6 transcription factors required for development and pathogenicity by high-throughput gene knockout in the rice blast fungus. PLoS Pathog 10: e1004432.
21. Chung H, Choi J, Park SY, Jeon J, Lee YH (2013) Two conidiation-related Zn(II)2Cys6 transcription factor genes in the rice blast fungus. Fungal Genetics and Biology 61: 133-141.

22. Battaglia E, Klaubauf S, Vallet J, Ribot C, Lebrun MH, et al. (2013) Xlr1 is involved in the transcriptional control of the pentose catabolic pathway, but not hemi-cellulolytic enzymes in *Magnaporthe oryzae*. Fungal Genetics and Biology 57: 76-84.
23. Klaubauf S, Zhou M, Lebrun MH, de Vries RP, Battaglia E (2016) A novel L-arabinose-responsive regulator discovered in the rice-blast fungus *Pyricularia oryzae* (*Magnaporthe oryzae*). FEBS Lett 590: 550-558.
24. Dong Y, Zhao Q, Liu X, Zhang X, Qi Z, et al. (2015) MoMyb1 is required for asexual development and tissue-specific infection in the rice blast fungus *Magnaporthe oryzae*. BMC Microbiology 15: 37.
25. Kim S, Park SY, Kim KS, Rho HS, Chi MH, et al. (2009) Homeobox Transcription Factors Are Required for Conidiation and Appressorium Development in the Rice Blast Fungus *Magnaporthe oryzae*. PLoS Genetics 5: e1000757.
26. Liu WD, Xie SY, Zhao XH, Chen X, Zheng WH, et al. (2010) A Homeobox Gene Is Essential for Conidiogenesis of the Rice Blast Fungus *Magnaporthe oryzae*. Molecular Plant-Microbe Interactions 23: 366-375.
27. Park G, Bruno KS, Staiger CJ, Talbot NJ, Xu J-R (2004) Independent genetic mechanisms mediate turgor generation and penetration peg formation during plant infection in the rice blast fungus. Molecular Microbiology 53: 1695-1707.
28. Park G, Xue C, Zheng L, Lam S, Xu JR (2002) MST12 Regulates Infectious Growth but not Appressorium Formation in the Rice Blast Fungus *Magnaporthe grisea*. Molecular Plant-Microbe Interactions 15: 183-192.
29. Sweigard JA, Carroll AM, Farrall L, Chumley FG, Valent B (1998) *Magnaporthe grisea* Pathogenicity Genes Obtained Through Insertional Mutagenesis. Molecular Plant-Microbe Interactions 11: 404-412.
30. Kong S, Park SY, Lee YH (2015) Systematic characterization of the bZIP transcription factor gene family in the rice blast fungus, *Magnaporthe oryzae*. Environmental Microbiology 17: 1425-1443.
31. Guo M, Guo W, Chen Y, Dong SM, Zhang X, et al. (2010) The Basic Leucine Zipper Transcription Factor Moatf1 Mediates Oxidative Stress Responses and Is Necessary for Full Virulence of the Rice Blast Fungus *Magnaporthe oryzae*. Molecular Plant-Microbe Interactions 23: 1053-1068.
32. Guo M, Chen Y, Du Y, Dong YH, Guo W, et al. (2011) The bZIP Transcription Factor MoAP1 Mediates the Oxidative Stress Response and Is Critical for Pathogenicity of the Rice Blast Fungus *Magnaporthe oryzae*. PLoS Pathogens 7: e1001302.
33. Tang W, Ru Y, Hong L, Zhu Q, Zuo R, et al. (2015) System-wide characterization of bZIP transcription factor proteins involved in infection-related morphogenesis of *Magnaporthe oryzae*. Environmental Microbiology 17: 1377-1396.
34. Li GT, Zhou XY, Kong LG, Wang YL, Zhang H, et al. (2011) MoSfl1 Is Important for Virulence and Heat Tolerance in *Magnaporthe oryzae*. PLoS ONE 6: e19951.
35. Li Y, Liang S, Yan X, Wang H, Li D, et al. (2010) Characterization of MoLDB1 required for vegetative growth, infection-related morphogenesis, and pathogenicity in the rice blast fungus *Magnaporthe oryzae*. Molecular Plant-Microbe Interactions 23: 1260-1274.
36. Chen Y, Zhai S, Sun Y, Li M, Dong Y, et al. (2015) MoTup1 is required for growth, conidiogenesis and pathogenicity of *Magnaporthe oryzae*. Molecular Plant Pathology.
37. Lu JP, Feng XX, Liu XH, Lu Q, Wang HK, et al. (2007) Mnh6, a nonhistone protein, is required for fungal development and pathogenicity of *Magnaporthe grisea*. Fungal Genetics and Biology 44: 819-829.
38. Yang J, Zhao XY, Sun J, Kang ZS, Ding SL, et al. (2010) A Novel Protein Com1 Is Required for Normal Conidium Morphology and Full Virulence in *Magnaporthe oryzae*. Molecular Plant-Microbe Interactions 23: 112-123.
39. Shin J-H, Han J-H, Kim K (2014) Genome-wide analyses of DNA-binding proteins harboring AT-hook motifs and their functional roles in the rice blast pathogen, *Magnaporthe oryzae*. Genes & Genomics 36: 871-881.
40. Chen Y, Zhai S, Zhang H, Zuo R, Wang J, et al. (2013) Shared and distinct functions of two Gti1/Pac2 family proteins in growth, morphogenesis and pathogenicity of *Magnaporthe oryzae*. Environmental Microbiology 16: 788-801.
41. Wilson RA, Gibson RP, Quispe CF, Littlechild JA, Talbot NJ (2010) An NADPH-dependent genetic switch regulates plant infection by the rice blast fungus. Proceedings of the National Academy of Sciences of the United States of America 107: 21902-21907.
42. Fernandez J, Wright JD, Hartline D, Quispe CF, Madayiputhiya N, et al. (2012) Principles of Carbon Catabolite Repression in the Rice Blast Fungus: Tps1, Nmr1-3, and a MATE-Family Pump Regulate Glucose Metabolism during Infection. PLoS Genetics 8: ARTN e1002673.
43. Lau GW, Hamer JE (1998) Acropetal: a genetic locus required for conidiophore architecture and pathogenicity in the rice blast fungus. Fungal Genetics and Biology 24: 228-239.
44. Nishimura M, Hayashi N, Jwa NS, Lau GW, Hamer JE, et al. (2000) Insertion of the LINE Retrotransposon MGL Causes a Conidiophore Pattern Mutation in *Magnaporthe grisea*. Molecular Plant-Microbe Interactions 13: 892-894.
