# Supplementary material for: Tpc1 is an important Zn(II)2Cys6 transcriptional regulator required for polarized growth and virulence in the rice blast fungus
Source: PLoS Pathog. 2017 Jul 24;13(7):e1006516. doi: 10.1371/journal.ppat.1006516 (PMC5542705; doi:10.1371/journal.ppat.1006516)
Supplement: S2 Table — (PDF) [file ppat.1006516.s010.pdf]

S2 Table. Complete list of $Zn(II)_2Cys_6$ binuclear family of transcriptional regulators present in *M. oryzae* genome.

	Gene locus	Motifs*	aa
**1**	MGG_00021	1	376
**2**	MGG_00032	2	552
**3**	MGG_00049	2	708
**4**	MGG_00096	1	590
**5**	MGG_00318	1	863
**6**	MGG_00320	1	518
**7**	MGG_00329	2	708
**8**	MGG_00417	2	464
**9**	MGG_00494	0	676
**10**	MGG_00672	1	740
**11**	MGG_00934	1	995
**12 (Tpc1)**	**MGG_01285**	1	**839**
**13**	MGG_01414	2	995
**14**	MGG_01486	2	1,074
**15**	MGG_01518	2	988
**16**	MGG_01624	2	1,023
**17**	MGG_01734	1	698
**18**	MGG_01777	2	960
**19**	MGG_01779	1	637
**20**	MGG_01833	2	781
**21**	MGG_01836	1	941
**22**	MGG_01870	1	504
**23**	MGG_01887	2	1017
**24**	MGG_01946	1	522
**25**	MGG_02089	1	518
**26**	MGG_02226	2	849
**27**	MGG_02289	1	475
**28**	MGG_02377	2	883
**29**	MGG_02408	2	928

	Gene locus	Motifs	aa
**30**	MGG_02595	1	694
**31**	MGG_02866	2	759
**32**	MGG_02879	2	814
**33**	MGG_02880	1	827
**34**	MGG_02962	1	482
**35**	MGG_03055	1	721
**36**	MGG_03183	1	478
**37**	MGG_03463	1	731
**38**	MGG_03669	2	819
**39**	MGG_03711	1	364
**40**	MGG_03763	2	592
**41**	MGG_03848	1	742
**42**	MGG_03939	2	1,022
**43**	MGG_04108	2	912
**44**	MGG_04141	1	678
**45**	MGG_04213	1	589
**46**	MGG_04326	1	772
**47**	MGG_04360	1	644
**48**	MGG_04387	2	667
**49**	MGG_04571	1	666
**50**	MGG_04674	1	841
**51**	MGG_04843	1	599
**52**	MGG_04933	1	560
**53**	MGG_05033	1	742
**54**	MGG_05153	1	552
**55**	MGG_05343	2	724
**56**	MGG_05434	0	663
**57**	MGG_05459	1	760
**58**	MGG_05578	1	499

	Gene locus	Motifs	aa
**59**	MGG_05659	1	486
**60**	MGG_05683	1	973
**61**	MGG_05724	2	713
**62**	MGG_05829	2	656
**63**	MGG_05845	1	622
**64**	MGG_05891	1	585
**65**	MGG_05939	2	725
**66**	MGG_06243	2	1,114
**67**	MGG_06279	1	969
**68**	MGG_06312	1	813
**69**	MGG_06355	2	879
**70**	MGG_06416	2	658
**71**	MGG_06455	1	731
**72**	MGG_06492	2	776
**73**	MGG_06550	1	640
**74**	MGG_06626	1	580
**75**	MGG_06778	2	724
**76**	MGG_06832	1	692
**77**	MGG_06954	1	670
**78**	MGG_07063	1	640
**79**	MGG_07131	1	842
**80**	MGG_07149	0	727
**81**	MGG_07215	1	973
**82**	MGG_07218	1	450
**83**	MGG_07368	1	630
**84**	MGG_07386	1	709
**85**	MGG_07450	1	873
**86**	MGG_07458	1	875
**87**	MGG_07534	1	584

*: 0, $Zn(II)_2Cys_6$ domain not annotated in INTERPRO; 1, Zn(II)2Cys6 domain; 2, presence of additional domains other than $Zn(II)_2Cys_6$

	Gene locus	Motifs*	aa
**88**	MGG_07549	1	570
**89**	MGG_07636	2	616
**90**	MGG_07681	2	819
**91**	MGG_07777	2	742
**92**	MGG_07800	1	741
**93**	MGG_07830	1	473
**94**	MGG_07845	1	564
**95**	MGG_08058	0	337
**96**	MGG_08093	1	719
**97**	MGG_08094	1	645
**98**	MGG_08130	3	702
**99**	MGG_08168	1	717
**100**	MGG_08185	1	564
**101**	MGG_08199	2	1,009
**102**	MGG_08314	2	894
**103**	MGG_08361	1	528
**104**	MGG_08618	2	616
**105**	MGG_08753	1	316
**106**	MGG_08777	2	823
**107**	MGG_08784	3	971
**108**	MGG_08917	1	648
**109**	MGG_08974	2	704
**110**	MGG_09002	1	215
**111**	MGG_09027	1	770
**112**	MGG_09118	1	747
**113**	MGG_09263	1	1,099
**114**	MGG_09273	1	820
**115**	MGG_09276	1	971
**116**	MGG_09312	1	457

	Gene locus	Motifs	aa
**117**	MGG_09676	1	704
**118**	MGG_09780	1	539
**119**	MGG_09829	1	787
**120**	MGG_09950	1	957
**121**	MGG_10197	1	713
**122**	MGG_10212	2	651
**123**	MGG_10246	1	468
**124**	MGG_10307	1	751
**125**	MGG_10422	0	703
**126**	MGG_10528	2	1,002
**127**	MGG_10529	1	686
**128**	MGG_10694	1	755
**129**	MGG_10806	1	524
**130**	MGG_11116	2	809
**131**	MGG_11119	2	1,024
**132**	MGG_11160	1	657
**133**	MGG_11192	1	1,103
**134**	MGG_11247	0	613
**135**	MGG_11724	1	362
**136**	MGG_11764	1	582
**137**	MGG_12037	1	760
**138**	MGG_12097	1	793
**139**	MGG_12339	1	729
**140**	MGG_12349	1	659
**141**	MGG_12424	0	608
**142**	MGG_12776	2	729
**143**	MGG_13350	2	471
**144**	MGG_13360	1	597
**145**	MGG_13385	1	811

	Gene locus	Motifs	aa
**146**	MGG_13629	2	867
**147**	MGG_13894	1	610
**148**	MGG_13919	2	776
**149**	MGG_13927	1	498
**150**	MGG_13994	1	680
**151**	MGG_14175	2	886
**152**	MGG_14728	1	533
**153**	MGG_14816	1	664
**154**	MGG_14852	1	721
**155**	MGG_15021	1	485
**156**	MGG_15023	1	951
**157**	MGG_15085	2	880
**158**	MGG_15093	1	609
**159**	MGG_15139	1	1,167
**160**	MGG_15759	1	335
**161**	MGG_15915	2	675
**162**	MGG_16161	2	871
**163**	MGG_16444	2	900
**164**	MGG_16477	2	770
**165**	MGG_16756	2	668
**166**	MGG_16835	1	531
**167**	MGG_17012	1	766
**168**	MGG_17060	1	821
**169**	MGG_17264	1	419
**170**	MGG_17623	2	656
**171**	MGG_17669	2	925
**172**	MGG_17821	1	664
**173**	MGG_17841	1	555
**174**	MGG_18016	1	130
**175**	MGG_18117	0	202

*: 0, $Zn(II)_2Cys_6$ domain not annotated in INTERPRO; 1, Zn(II)2Cys6 domain; 2, presence of additional domains other than $Zn(II)_2Cys_6$
