# Supplementary material for: Tpc1 is an important Zn(II)2Cys6 transcriptional regulator required for polarized growth and virulence in the rice blast fungus
Source: PLoS Pathog. 2017 Jul 24;13(7):e1006516. doi: 10.1371/journal.ppat.1006516 (PMC5542705; doi:10.1371/journal.ppat.1006516)
Supplement: S3 Table — (PDF) [file ppat.1006516.s011.pdf]

S3 Table. List of differentially expressed genes in Δ*tpc1* identified by transcriptome analysis

[1]Using a cut-off value of two-fold change with FDR LiMMA<0.01, 400 genes were up/down regulated. Gene probes had at least an average log2 signal intensity larger than 8.

[2]Promoter regions of underline genes contain a potential Zn2(Cys)6 binuclear cluster binding domain (Supplemental Figure S6). The five genes selected for gene replacement are indicated in bold.

[3]*S. cerevisiae* orthologues unless indicated.

[4]References for *M. oryzae* genes

Fold change[1]	GENE ID[2]	PROTEIN DESCRIPTION / ORTHOLOGUES[3]	GO term name and number	REFERENCES[4]
**Signaling (3 down-regulated; 10 up-regulated)**				
-10,7	**MGG_06800**	Pebp1; Phosphatidylethanolamine-binding protein PEBP		this study
-6,6	**MGG_14045**	Pebp2; Phosphatidylethanolamine-binding protein PEBP		this study
-5,9	MGG_04787	Leucine carboxyl methyltransferase, its activity may affect the heteromultimeric composition of protein phosphatase 2A	methyltransferase activity GO:0008168; methylation GO:0032259; transferase activity GO:0016740	
7,0	MGG_01779	Zn_2C_6 fungal-type transcriptional regulator; not required for conidiation, growth and virulence but affected in stress response	Nucleus GO:0005634; zinc ion binding GO:0008270; regulation of transcription, DNA-templated GO:0006355; sequence-specific DNA binding transcription factor activity GO:0003700; sequence-specific DNA binding RNA polymerase II transcription factor activity GO:0000981	[1]
4,9	MGG_14499	Serine/threonine protein kinase; overexpressed after the transition from darkness to light[2] / some similarity with Ime2 (e-3) and Rim11 (3,2e-3) of *S. cerevisiae,* kinases that regulate activation of meiosis in yeast.	ATP binding GO:0005524; kinase activity GO:0016301; phosphorylation GO:0016310; protein kinase activity GO:0004672; protein serine/threonine kinase activity GO:0004674; protein phosphorylation GO:0006468; transferase activity, transferring phosphorus-containing groups GO:0016772	
3,6	MGG_00070	Zn_2C_6 fungal-type DNA-binding domain; pathogenicity, appressorium formation, germination and conidiation affected in the mutant; not listed in [1]	Binding GO:0005488; nucleus GO:0005634; zinc ion binding GO:0008270; regulation of transcription, DNA-templated GO:0006355; sequence-specific DNA binding transcription factor activity GO:0003700; sequence-specific DNA binding RNA polymerase II transcription factor activity GO:0000981	[4]
3,5	MGG_00080	C_2H_2 Zinc finger DNA-binding domain	nucleic acid binding GO:0003676; zinc ion binding GO:0008270; intracellular GO:0005622; metal ion binding GO:0046872	
2,3	MGG_09138	glutathione S-transferase; participate in the detoxification of reactive electrophillic compounds by catalysing their conjugation to glutathione.	cytoplasmic sequestering of protein GO:0051220; negative regulation of nucleic acid-templated transcription GO:1903507; phosphoprotein binding GO:0051219; response to aluminum ion GO:0010044; cytoplasmic sequestering of transcription factor GO:0042994; glutathione peroxidase activity GO:0004602; soluble fraction GO:0005625; transcription corepressor activity GO:0003714; cytosol GO:0005829; protein urmylation GO:0032447;	
2,2	MGG_06120	Homeodomain-like domain; Myb domain; overexpressed in planta and during germination and ionic stress (TF052) [3]	DNA binding GO:0003677	
2,2	MGG_01475	NTD80 domain; similar to yeast NTD80 (e-10), and *N. crassa* Fsd-1 (e-100), which is involved in female sexual development and ascospore maturation	DNA binding GO:0003677; regulation of transcription, DNA-templated GO:0006355; sequence-specific DNA binding transcription factor activity GO:0003700	
2,2	MGG_01624	FZC3; Zn_2C_6 fungal-type transcriptional regulator; mutant not affected in conidiation, growth, virulence or stress response; overexpressed during conidiation, germination, appressorium development, in planta and oxidative stress (TF170) [3]; not listed in [1]	metal ion binding GO:0046872; nucleus GO:0005634; DNA binding GO:0003677; zinc ion binding GO:0008270; transcription, DNA-templated GO:0006351; regulation of transcription, DNA-templated GO:0006355; sequence-specific DNA binding transcription factor activity GO:0003700	[1]

2,2	MGG_02055	APSES and ZnF C_2H_2 domains; transcriptional regulator	DNA binding GO:0003677; nucleic acid binding GO:0003676; zinc ion binding GO:0008270; regulation of transcription, DNA-templated GO:0006355; intracellular GO:0005622; sequence-specific DNA binding transcription factor activity GO:0003700; metal ion binding GO:0046872	
2,0	MGG_02061	CAMK protein kinase; mutant not affected in pathogenicity	ATP binding GO:0005524; kinase activity GO:0016301; phosphorylation GO:0016310; protein kinase activity GO:0004672; protein serine/threonine kinase activity GO:0004674; protein phosphorylation GO:0006468; transferase activity, transferring phosphorus-containing groups GO:0016772	[5]

**Metabolism (66 down-regulated; 61 up-regulated)**				
-9,8	MGG_15388	Lactose permease; TM	Membrane GO:0016020; integral component of membrane GO:0016021; transport GO:0006810; transmembrane transport GO:0055085; transporter activity GO:0005215; transmembrane transporter activity GO:0022857; substrate-specific transmembrane transporter activity GO:0022891	
-8,9	MGG_08359	Flavonol synthase; Oxoglutarate/iron-dependent oxygenase	oxidoreductase activity GO:0016491; oxidation-reduction process GO:0055114; iron ion binding GO:0005506; oxidoreductase activity, acting on paired donors, with incorporation or reduction of molecular oxygen, 2-oxoglutarate as one donor, and incorporation of one atom each of oxygen into both donors GO:0016706	
-8,7	MGG_07883	Short-chain dehydrogenase-reductase; NAD(P)-binding domain	nucleotide binding GO:0000166; metabolic process GO:0008152; oxidoreductase activity GO:0016491; oxidation-reduction process GO:0055114	
-8,0	MGG_08363	Alcohol dehydrogenase GroES-like/NAD(P)-binding domain; ApdC (*A. nidulans* ; e-64), member of the aspyridone (apd) gene cluster[6]	nucleotide binding GO:0000166; oxidoreductase activity GO:0016491; oxidation-reduction process GO:0055114; zinc ion binding GO:0008270	
-8,0	MGG_15100	Polyketide synthase; NAD(P)-binding domain; syn6	nucleotide binding GO:0000166; transferase activity GO:0016740; catalytic activity GO:0003824; metabolic process GO:0008152; binding GO:0005488; biosynthetic process GO:0009058; ACP phosphopantetheine attachment site binding involved in fatty acid biosynthetic process GO:0000036; phosphopantetheine binding GO:0031177	
-6,8	MGG_06950	L-serine dehydratase; Cha1p (*S. cerevisie* ; e-53)	catalytic activity GO:0003824; metabolic process GO:0008152; pyridoxal phosphate binding GO:0030170; cellular amino acid metabolic process GO:0006520	
-6,9	MGG_05010	P-loop containing nucleoside triphosphate hydrolase		
-6,1	MGG_03036	NmrA-like/NAD(P)-binding domain	nucleotide binding GO:0000166	
-5,9	MGG_06340	Butirosin biosynthesis, BtrG-like domain	glutathione biosynthetic process GO:0006750; gamma-glutamylcyclotransferase activity GO:0003839	
-4,9	MGG_08498	Cytochrome P450; oxidoreductase activity; TM	metal ion binding GO:0046872; oxidoreductase activity GO:0016491; oxidation-reduction process GO:0055114; heme binding GO:0020037; iron ion binding GO:0005506; monooxygenase activity GO:0004497; electron carrier activity GO:0009055; oxidoreductase activity, acting on paired donors, with incorporation or reduction of molecular oxygen GO:0016705	
-4,7	MGG_13895	Fructose-bisphosphate aldolase	catalytic activity GO:0003824; zinc ion binding GO:0008270; carbohydrate metabolic process GO:0005975; aldehyde-lyase activity GO:0016832	
-4,5	MGG_10893	Nicotinic acid transporter Tna1 (S. cerevisiae; e-54); TM	integral component of membrane GO:0016021; transmembrane transport GO:0055085	
-4,2	MGG_07419	Tyrosinase; SignalP-noTM	metabolic process GO:0008152; oxidoreductase activity GO:0016491; oxidation-reduction process GO:0055114; metal ion binding GO:0046872	

-4,2	MGG_00097	Putative NADP(+) coupled glycerol dehydrogenase; Gcy1 (*N.crassa* ; e-143); it can bind mRNA	oxidoreductase activity GO:0016491; oxidation-reduction process GO:0055114; mycelium development GO:0043581	
-4,1	MGG_08486	Beta-lactamase-related; SignalP-noTM		
-4,0	MGG_05479	GH43 domain; xylosidase/arabinosidase-Glycoside hydrolase	hydrolase activity, hydrolyzing O-glycosyl compounds GO:0004553; carbohydrate metabolic process GO:0005975	[7]
-4,0	MGG_08342	Phosphorylcholine phosphatase-like domain; SignalP-noTM		
-3,9	MGG_02530	Sugar/inositol transporter; TM; Hxt11 (S. cerevisiae; e-39)	Membrane GO:0016020; integral component of membrane GO:0016021; transport GO:0006810; transmembrane transport GO:0055085; transporter activity GO:0005215; transmembrane transporter activity GO:0022857; substrate-specific transmembrane transporter activity GO:0022891	
-3,7	MGG_10533	Agmatinase 1; Ureohydrolase; SignalP-TM	metal ion binding GO:0046872; hydrolase activity GO:0016787; hydrolase activity, acting on carbon-nitrogen (but not peptide) bonds, in linear amidines GO:0016813	
-3,6	MGG_09307	Hexose transporter; TM ; Stl1/2 (S. cerevisiae; e-31)	Membrane GO:0016020; integral component of membrane GO:0016021; transport GO:0006810; transmembrane transport GO:0055085; transporter activity GO:0005215; transmembrane transporter activity GO:0022857; substrate-specific transmembrane transporter activity GO:0022891	
-3,5	MGG_08291	Cupredoxin; SignalP-noTM; paralogue of MGG_06653	interaction with host via protein secreted by type II secretion system GO:0052051	
-3,5	MGG_04997	Oligopeptide transporter OPT; TM ; Opt1 (S. cerevisiae; e-116)	transmembrane transport GO:0055085; nucleus GO:0005634; cytosol GO:0005829	
-3,4	MGG_06653	Cupredoxin, multicopper oxidase; paralogue of MGG_08291	mycelium development GO:0043581	
-3,4	MGG_07247	Cytochrome P450; SignalP-TM	metal ion binding GO:0046872; oxidation-reduction process GO:0055114; heme binding GO:0020037; iron ion binding GO:0005506; monooxygenase activity GO:0004497; electron carrier activity GO:0009055; oxidoreductase activity, acting on paired donors, with incorporation or reduction of molecular oxygen GO:0016705	
-3,3	MGG_08474	Fatty acid desaturase; TM	lipid metabolic process GO:0006629	
-3,3	MGG_10860	Sterol 24-C-methyltransferase; ERGosterol biosynthesis methyltransferase; Erg6 (*S. cerevisiae* ; e-99)	transferase activity GO:0016740; metabolic process GO:0008152; methyltransferase activity GO:0008168; methylation GO:0032259; steroid biosynthetic process GO:0006694; mitochondrial outer membrane GO:0005741; sterol 24-C-methyltransferase activity GO:0003838; lipid particle GO:0005811	
-3,2	MGG_03263	Aldehyde/histidinol dehydrogenase; Ald3/5 (*S. cerevisiae* ; e-72)	metabolic process GO:0008152; oxidoreductase activity GO:0016491; oxidation-reduction process GO:0055114; oxidoreductase activity, acting on the aldehyde or oxo group of donors, NAD or NADP as acceptor GO:0016620	
-3,2	MGG_17097	Major facilitator superfamily; TM ; previously MGG_03409; Tna1 (S. cerevisiae; e-42)	integral component of membrane GO:0016021; transmembrane transport GO:0055085	
-3,1	MGG_08937	Sugar-inositol transporter; TM ; Hxt1/4 (S.cerevisiae; e-52)	Membrane GO:0016020; integral component of membrane GO:0016021; transport GO:0006810; transmembrane transport GO:0055085; transporter activity GO:0005215; transmembrane transporter activity GO:0022857; substrate-specific transmembrane transporter activity GO:0022891	
-3,1	MGG_07431	Glutamine amidotransferase type 1		

-3,0	MGG_02817	Glutamate decarboxylase; Gad1 (*S. cerevisiae* ; e-122); T-DNA mutant is not affected in virulence	catalytic activity GO:0003824; lyase activity GO:0016829; pyridoxal phosphate binding GO:0030170; glutamate decarboxylase activity GO:0004351; glutamate metabolic process GO:0006536; carboxy-lyase activity GO:0016831; carboxylic acid metabolic process GO:0019752; cytoplasm GO:0005737; cellular response to oxidative stress GO:0034599; glutamate catabolic process GO:0006538; calmodulin binding GO:0005516	[4]
-2,7	MGG_03648	Xyr1; NAD(P)H-dependent D-xylose reductase; similar to yeast Gre3	oxidoreductase activity GO:0016491; oxidation-reduction process GO:0055114; glucose 1-dehydrogenase (NADP+) activity GO:0047935; galactose catabolic process GO:0019388; D-xylose:NADP reductase activity GO:0032866; cellular response to osmotic stress GO:0071470; arabinose catabolic process GO:0019568; alditol:NADP+ 1-oxidoreductase activity GO:0004032; mRNA binding GO:0003729	[8,9]
-2,7	MGG_03038	P-loop_NTPase	ADP binding GO:0043531; mycelium development GO:0043581	
-2,6	MGG_00973	FAD binding domain-containing protein;SignalP-noTM	oxidoreductase activity GO:0016491; oxidation-reduction process GO:0055114; flavin adenine dinucleotide binding GO:0050660; UDP-N-acetylmuramate dehydrogenase activity GO:0008762; oxidoreductase activity, acting on CH-OH group of donors GO:0016614; catalytic activity GO:0003824; oxidoreductase activity GO:0016491	
-2,6	MGG_06095	Cysteine dioxygenase	oxidation-reduction process GO:0055114; iron ion binding GO:0005506; oxidoreductase activity, acting on single donors with incorporation of molecular oxygen, incorporation of two atoms of oxygen GO:0016702; cysteine dioxygenase activity GO:0017172; L-cysteine metabolic process GO:0046439	
-2,6	MGG_04345	Cytochrome P450 17A1; 1 TM	metal ion binding GO:0046872; oxidoreductase activity GO:0016491; oxidation-reduction process GO:0055114; heme binding GO:0020037; iron ion binding GO:0005506; monooxygenase activity GO:0004497; electron carrier activity GO:0009055; oxidoreductase activity, acting on paired donors, with incorporation or reduction of molecular oxygen GO:0016705	
-2,6	MGG_11327	Amino-acid permease; TM; similar to yeast Gap1 (e-124)	Membrane GO:0016020; integral component of membrane GO:0016021; transport GO:0006810; transmembrane transport GO:0055085; amino acid transmembrane transport GO:0003333; amino acid transport GO:0006865; amino acid transmembrane transporter activity GO:0015171	
-2,5	MGG_13764	cupredoxin	oxidoreductase activity GO:0016491; oxidation-reduction process GO:0055114; copper ion binding GO:0005507	
-2,5	MGG_10005	glycerol kinase; there are two in M.oryzae (MGG_01281); similar to yeast Gut1 (e-67)	kinase activity GO:0016301; phosphorylation GO:0016310; transferase activity GO:0016740; carbohydrate metabolic process GO:0005975; phosphotransferase activity, alcohol group as acceptor GO:0016773; glycerol kinase activity GO:0004370; glycerol-3-phosphate metabolic process GO:0006072	
-2,5	MGG_10408	FAD binding domain-containing protein; SignalP - noTM	catalytic activity GO:0003824; oxidoreductase activity GO:0016491; oxidation-reduction process GO:0055114; flavin adenine dinucleotide binding GO:0050660; UDP-N-acetylmuramate dehydrogenase activity GO:0008762; oxidoreductase activity, acting on CH-OH group of donors GO:0016614	

-2,5	MGG_00892	ATP synthase subunit 9; 2 TM ; similar to yeast Oli1	membrane GO:0016020; integral component of membrane GO:0016021; transport GO:0006810; ion transport GO:0006811; hydrogen ion transmembrane transporter activity GO:0015078; ATP hydrolysis coupled proton transport GO:0015991; proton-transporting two-sector ATPase complex, proton-transporting domain GO:0033177: ATP synthesis coupled proton transport GO:0015986; proton transport GO:0015992; lipid binding GO:0008289; proton-transporting ATP synthase complex, coupling factor F(o) GO:0045263	
-2,5	MGG_10859	cytochrome P450	oxidation-reduction process GO:0055114; peroxidase activity GO:0004601; response to oxidative stress GO:0006979; heme binding GO:0020037; iron ion binding GO:0005506; electron carrier activity GO:0009055; oxidoreductase activity, acting on paired donors, with incorporation or reduction of molecular oxygen GO:0016705	
-2,4	MGG_09757	Neutral alpha-glucosidase; GH31	catalytic activity GO:0003824; hydrolase activity, hydrolyzing O-glycosyl compounds GO:0004553; carbohydrate metabolic process GO:0005975; carbohydrate binding GO:0030246; carbohydrate metabolic process GO:0005975; catalytic activity GO:0003824	
-2,4	MGG_00743	Twin-arginine translocation pathway signal	metabolic process GO:0008152; catalytic activity GO:0003824; epoxide hydrolase activity GO:0004301; soluble fraction GO:0005625	
-2,4	MGG_04340	transferase	transferase activity, transferring acyl groups other than amino-acyl groups GO:0016747; mycelium development GO:0043581	
-2,4	MGG_10106	NmrA domain; NAD(P)-binding	nucleotide binding GO:0000166	
-2,3	MGG_09852	Sugar transporter; similar to yeast Stl1 (e-123); TM	Membrane GO:0016020; integral component of membrane GO:0016021; transport GO:0006810; transmembrane transport GO:0055085; carbohydrate transport GO:0008643; transporter activity GO:0005215; transmembrane transporter activity GO:0022857; substrate-specific transmembrane transporter activity GO:0022891	
-2,3	MGG_10751	Peroxisomal copper amine oxidase	metal ion binding GO:0046872; oxidoreductase activity GO:0016491; oxidation-reduction process GO:0055114; copper ion binding GO:0005507; quinone binding GO:0048038; primary amine oxidase activity GO:0008131; amine metabolic process GO:0009308	
-2,2	MGG_12175	Gramicidin S synthetase 1; AMP-dependent synthetase/Ligase; DUF4009	catalytic activity GO:0003824; metabolic process GO:0008152; ligase activity GO:0016874; cofactor binding GO:0048037; ACP phosphopantetheine attachment site binding involved in fatty acid biosynthetic process GO:0000036; phosphopantetheine binding GO:0031177	
-2,2	MGG_10334	Mannitol 2-dehydrogenase	coenzyme binding GO:0050662; metabolic process GO:0008152; catalytic activity GO:0003824; oxidoreductase activity GO:0016491; oxidation-reduction process GO:0055114; oxidoreductase activity, acting on the CH-OH group of donors, NAD or NADP as acceptor GO:0016616	
-2,2	MGG_07982	Cytochrome P450 monooxygenase	metal ion binding GO:0046872; oxidoreductase activity GO:0016491; oxidation-reduction process GO:0055114; heme binding GO:0020037; iron ion binding GO:0005506; monooxygenase activity GO:0004497; electron carrier activity GO:0009055	
-2,2	MGG_03793	2,3-dihydroxybenzoic acid decarboxylase	catalytic activity GO:0003824; metabolic process GO:0008152	
-2,2	MGG_10583	12-oxophytodienoate reductase 1	catalytic activity GO:0003824; oxidoreductase activity GO:0016491; oxidation-reduction process GO:0055114; FMN binding GO:0010181	

-2,2	MGG_06888	Glutamine synthetase	nucleotide binding GO:0000166; ATP binding GO:0005524; catalytic activity GO:0003824; ligase activity GO:0016874; nitrogen compound metabolic process GO:0006807; glutamate-ammonia ligase activity GO:0004356; glutamine biosynthetic process GO:0006542; cytoplasm GO:0005737	
-2,2	MGG_08173	NADP-dependent malic enzyme	nucleotide binding GO:0000166; metal ion binding GO:0046872; oxidoreductase activity GO:0016491; oxidoreductase activity, acting on the CH-OH group of donors, NAD or NADP as acceptor GO:0016616; oxidation-reduction process GO:0055114; NAD binding GO:0051287; malate metabolic process GO:0006108; malic enzyme activity GO:0004470	
-2,1	MGG_06644	Tartrate transporter	integral component of membrane GO:0016021; transmembrane transport GO:0055085	
-2,1	MGG_10561	tyrosinase; SignalP - noTM	metal ion binding GO:0046872; metabolic process GO:0008152; oxidoreductase activity GO:0016491; oxidation-reduction process GO:0055114	
-2,1	MGG_07200	Plasma membrane ATPase; TM ; similar to yeast Pma1	nucleotide binding GO:0000166; ATP binding GO:0005524; metal ion binding GO:0046872; catalytic activity GO:0003824; metabolic process GO:0008152; membrane GO:0016020; integral component of membrane GO:0016021; hydrolase activity GO:0016787; obsolete ATP catabolic process GO:0006200; ATPase activity GO:0016887; cation transport GO:0006812; ATP biosynthetic process GO:0006754; ATPase activity, coupled to transmembrane movement of ions, phosphorylative mechanism GO:0015662;	
-2,1	MGG_08281	Polyketide synthase	transferase activity GO:0016740; catalytic activity GO:0003824; metabolic process GO:0008152; hydrolase activity, acting on ester bonds GO:0016788; binding GO:0005488; biosynthetic process GO:0009058; cofactor binding GO:0048037; ACP phosphopantetheine attachment site binding involved in fatty acid biosynthetic process GO:0000036; phosphopantetheine binding GO:0031177	
-2,1	MGG_11916	CAP20; transaldolase	mycelium development GO:0043581	
-2,1	MGG_14886	sphingolipid C9-methyltransferase ; TM	transferase activity GO:0016740; lipid biosynthetic process GO:0008610; methyltransferase activity GO:0008168; methylation GO:0032259; lipid biosynthetic process GO:0008610	
-2,1	MGG_08468	Fatty acyl-CoA hydrolase; SignalP - noTM	hydrolase activity GO:0016787	
-2,0	MGG_04225	MFS quinate transporter; 12 TM	Membrane GO:0016020; integral component of membrane GO:0016021; transport GO:0006810; transmembrane transport GO:0055085; transporter activity GO:0005215; transmembrane transporter activity GO:0022857; substrate-specific transmembrane transporter activity GO:0022891	
-2,0	MGG_07793	Dehydrogenase/reductase SDR family member 13	nucleotide binding GO:0000166; metabolic process GO:0008152; oxidoreductase activity GO:0016491; oxidation-reduction process GO:0055114	
-2,0	MGG_07457	cyclase	tryptophan catabolic process to kynurenine GO:0019441; arylformamidase activity GO:0004061	
-2,0	MGG_01114	NADP-dependent mannitol dehydrogenase; SignalP-noTM	nucleotide binding GO:0000166; metal ion binding GO:0046872; oxidoreductase activity GO:0016491; oxidation-reduction process GO:0055114; zinc ion binding GO:0008270	
56,1	MGG_09601	Glycoside hydrolase, GH31; Alpha-xylosidase; carbohydrate metabolism; Rot2 (*S. cerevisiae* ; e-25)	catalytic activity GO:0003824; hydrolase activity, hydrolyzing O-glycosyl compounds GO:0004553; carbohydrate metabolic process GO:0005975; carbohydrate binding GO:0030246	

12,3	MGG_09602	Copper amine oxidase/Copper amine oxidase; SignalP-noTM; co-immunoprecipitates with the histone deacetylase complex Tig1 [10]	oxidation-reduction process GO:0055114; copper ion binding GO:0005507; quinone binding GO:0048038; primary amine oxidase activity GO:0008131; amine metabolic process GO:0009308	
10,3	MGG_09608	Glycoside hydrolase GH28, Pectin lyase fold	metabolic process GO:0008152; hydrolase activity GO:0016787; carbohydrate metabolic process GO:0005975; hydrolase activity, acting on glycosyl bonds GO:0016798; polygalacturonase activity GO:0004650	
6,4	MGG_05046	adenine phosphoribosyltransferase; catalyzes the formation of AMP from adenine and 5-phosphoribosylpyrophosphate: it also contains a higher eukaryotic phosphomevalonate kinase/FAD linked oxidase; overexpressed after the transition from darkness to light [2]; APT1 (e-11)	transferase activity GO:0016740; catalytic activity GO:0003824; oxidoreductase activity GO:0016491; oxidation-reduction process GO:0055114; cytoplasm GO:0005737; flavin adenine dinucleotide binding GO:0050660; UDP-N-acetylmuramate dehydrogenase activity GO:0008762; oxidoreductase activity, acting on CH-OH group of donors GO:0016614; nucleoside metabolic process GO:0009116; transferase activity, transferring glycosyl groups GO:0016757; phosphomevalonate kinase activity GO:0004631; cholesterol biosynthetic process GO:0006695; cellular nitrogen compound biosynthetic process GO:0044271; cellular nitrogen compound biosynthetic process GO:0044271	
6,0	MGG_03348	phosphate-repressible phosphate permease; N.crassa Pho4 (NCU09564) and yeast PHO89 (e-130); Pho4 is a sodium-phosphate symporter	membrane GO:0016020; inorganic phosphate transmembrane transporter activity GO:0005315; phosphate ion transport GO:0006817; sodium:inorganic phosphate symporter activity GO:0015319; phosphate ion transmembrane transport GO:0035435; sodium ion transmembrane transport GO:0035725; transmembrane transport GO:0055085; plasma membrane GO:0005886; identical protein binding GO:0042802	
5,7	MGG_05045	GTP cyclohydrolase I; catalyses the biosynthesis of formic acid and dihydroneopterin triphosphate from GTP; Nitrile oxidoreductase; Fol2 (e-42)	Cytoplasm GO:0005737; hydrolase activity GO:0016787; GTP cyclohydrolase I activity GO:0003934; tetrahydrofolate biosynthetic process GO:0046654	
5,4	MGG_17476	Dienelactone hydrolase; play a crucial role in chlorocatechol degradation via the modified ortho cleavage pathway; *S. cerevisiae* AIM2 (e-23)	hydrolase activity GO:0016787	
5,0	MGG_08890	uncharacterized protein	cellular nitrogen compound biosynthetic process GO:0044271	
4,8	MGG_00402	polyketide synthase; short-chain dehydrogenase.reductase SDR/Glucose.ribitol dehydrogenase/NAD(P)-binding domain	nucleotide binding GO:0000166; metabolic process GO:0008152; oxidoreductase activity GO:0016491; oxidation-reduction process GO:0055114	
4,7	MGG_11496	RTA-like protein; TM; S. cerevisiae RSB1 (e-16) putative integral membrane transporter or flippase that may transport long chain bases (LCBs) from the cytoplasmic side toward the extracytoplasmic side of the membrane	integral component of membrane GO:0016021; response to stress GO:0006950	
4,7	MGG_08725	dehydrogenase, NAD-binding/NAD(P)-binding domain; SER3 (e-26), 3-phosphoglycerate dehydrogenase; catalyzes the first step in serine and glycine biosynthesis	nucleotide binding GO:0000166; metabolic process GO:0008152; oxidoreductase activity GO:0016491; oxidoreductase activity, acting on the CH-OH group of donors, NAD or NADP as acceptor GO:0016616; oxidation-reduction process GO:0055114; cofactor binding GO:0048037; NAD binding GO:0051287; mycelium development GO:0043581	
4,6	MGG_04126	CYS-14; TM, similar to S. cerevisiae Sul1 (e-139) and Sul2 (e-138); high affinity sulfate permease of the SulP anion transporter family	Membrane GO:0016020; integral component of membrane GO:0016021; transport GO:0006810; transmembrane transport GO:0055085; transporter activity GO:0005215; secondary active sulfate transmembrane transporter activity GO:0008271; sulfate transport GO:0008272; sulfate transmembrane transporter activity GO:0015116	
4,1	MGG_01511	MFS transporter; ABC transporter Flr1 (S.cerevisiae; e-81); TM	integral component of membrane GO:0016021; transmembrane transport GO:0055085; fluconazole transport GO:0015903; drug transmembrane transport GO:0006855; fluconazole transporter activity GO:0015244; plasma membrane GO:0005886	

3,7	MGG_03476	Protein-L-isoaspartate O-methyltransferase	transferase activity GO:0016740; methyltransferase activity GO:0008168; methylation GO:0032259; cellular protein modification process GO:0006464; protein-L-isoaspartate (D-aspartate) O-methyltransferase activity GO:0004719; cytoplasm GO:0005737; nucleus GO:0005634; cytosol GO:0005829	
3,5	MGG_11021	ATPase, AAA+ type; it is overexpressed after the transition from darkness to light[9]	nucleotide binding GO:0000166; ATP binding GO:0005524; nucleoside-triphosphatase activity GO:0017111; mycelium development GO:0043581	
3,4	MGG_05398	uncharacterized protein	cellular nitrogen compound biosynthetic process GO:0044271	
3,4	MGG_09607	Maltose permease; TM ; MAL31 (e-92) member of the 12 transmembrane domain superfamily of sugar transporters	membrane GO:0016020; integral component of membrane GO:0016021; transport GO:0006810; transmembrane transport GO:0055085; transporter activity GO:0005215; transmembrane transporter activity GO:0022857; substrate-specific transmembrane transporter activity GO:0022891	
3,3	MGG_17802	Aminotransferase, class V; Cysteine desulfurase; NFS1 (e-4); in yeast involved in iron-sulfur cluster (Fe/S) biogenesis and in thio-modification of mitochondrial and cytoplasmic tRNAs; essential protein located predominantly in mitochondria	catalytic activity GO:0003824; metabolic process GO:0008152; pyridoxal phosphate binding GO:0030170; nucleus GO:0005634; cytosol GO:0005829; mycelium development GO:0043581; ergothioneine biosynthetic process GO:0052699; hercynylcysteine sulfoxide lyase activity (ergothioneine-forming) GO:1990411; selenoneine biosynthetic process GO:1903257	
3,3	MGG_00020	SUMF1; required for post-translational sulfatase modification; Sulfatase-modifying factor enzyme	binding GO:0005488	
3,3	MGG_00832	Cytochrome P450; TM; ERG5 (e-6) C-22 sterol desaturase; a cytochrome P450 enzyme involved in ergosterol biosynthesis	metal ion binding GO:0046872; oxidoreductase activity GO:0016491; oxidation-reduction process GO:0055114; heme binding GO:0020037; iron ion binding GO:0005506; monooxygenase activity GO:0004497; electron carrier activity GO:0009055; oxidoreductase activity, acting on paired donors, with incorporation or reduction of molecular oxygen GO:0016705; mycelium development GO:0043581; membrane GO:0016020	
3,2	MGG_06373	Aromatic-ring hydroxylase-like-metabolic process,oxidoreductase activity	metabolic process GO:0008152; oxidoreductase activity GO:0016491; oxidation-reduction process GO:0055114	
3,1	MGG_10274	Short-chain dehydrogenase reductase SDR/Glucose-ribitol dehydrogenase/NAD(P)-binding domain	nucleotide binding GO:0000166; metabolic process GO:0008152; oxidoreductase activity GO:0016491; oxidation-reduction process GO:0055114; mycelium development GO:0043581	
3,1	MGG_04304	Short-chain dehydrogenase.reductase SDR/Glucose.ribitol dehydrogenase/NAD(P)-binding domain; YMR226C (e-16) is a NADP(+)-dependent serine dehydrogenase and carbonyl reductase; acts on serine, L-allo-threonine, and other 3-hydroxy acids	nucleotide binding GO:0000166; metabolic process GO:0008152; oxidoreductase activity GO:0016491; oxidation-reduction process GO:0055114	
3,1	MGG_05554	Alcohol dehydrogenase superfamily, zinc-type/GroES-like/NAD(P)-binding domain; ADH3 (e-7) Mitochondrial alcohol dehydrogenase involved in the shuttling of mitochondrial NADH to the cytosol under anaerobic conditions and ethanol production	nucleotide binding GO:0000166; oxidoreductase activity GO:0016491; oxidation-reduction process GO:0055114; zinc ion binding GO:0008270	
3,0	MGG_09359	Alcohol dehydrogenase superfamily, zinc-type/GroES-like/NAD(P)-binding domain; Xyl2 (e-7) xylitol dehydrogenase; converts xylitol to D-xylulose; expression induced by xylose, even though this pentose sugar is not well utilized by *S. cerevisiae*	nucleotide binding GO:0000166; oxidoreductase activity GO:0016491; oxidation-reduction process GO:0055114; zinc ion binding GO:0008270	
3,0	MGG_16375	Aldo-keto reductase/NADP-dependent oxidoreductase domain	oxidoreductase activity GO:0016491; oxidation-reduction process GO:0055114	
3,0	MGG_02210	type 2 phosphatidic acid phosphatase (PAP), enzymatic activity with a central role in the synthesis of phospholipids and triacylglycerol through its product diacylglycerol, and it also generates and/or degrades lipid-signalling molecules.	catalytic activity GO:0003824; oxidation-reduction process GO:0055114; membrane GO:0016020; peroxidase activity GO:0004601	

2,8	MGG_06326	Vacuolar ATP synthase V0 subunit; TM	Membrane GO:0016020; integral component of membrane GO:0016021; transport GO:0006810; ion transport GO:0006811; hydrogen ion transmembrane transporter activity GO:0015078; ATP hydrolysis coupled proton transport GO:0015991; proton-transporting two-sector ATPase complex, proton-transporting domain GO:0033177; proton-transporting V-type ATPase, V0 domain GO:0033179; proton transport GO:0015992	
2,7	MGG_05555	monooxygenase	oxidation-reduction process GO:0055114; monooxygenase activity GO:0004497; oxidoreductase activity, acting on paired donors, with incorporation or reduction of molecular oxygen GO:0016705	
2,7	MGG_10710	Oxidoreductase	metabolic process GO:0008152; oxidoreductase activity GO:0016491; oxidation-reduction process GO:0055114	
2,7	MGG_08074	NADP-specific glutamate dehydrogenase	nucleotide binding GO:0000166; oxidoreductase activity GO:0016491; oxidation-reduction process GO:0055114; cellular amino acid metabolic process GO:0006520; oxidoreductase activity, acting on the CH-NH2 group of donors, NAD or NADP as acceptor GO:0016639; cytosol GO:0005829; mitochondrion GO:0005739; nucleus GO:0005634	
2,6	MGG_16853	Putative uncharacterized protein ; previously MGG_05055	oxidation-reduction process GO:0055114; flavin adenine dinucleotide binding GO:0050660; oxidoreductase activity, acting on CH-OH group of donors GO:0016614; alcohol metabolic process GO:0006066; choline dehydrogenase activity GO:0008812	
2,6	MGG_07219	Conidial yellow pigment biosynthesis polyketide synthase	transferase activity GO:0016740; catalytic activity GO:0003824; metabolic process GO:0008152; hydrolase activity, acting on ester bonds GO:0016788; binding GO:0005488; biosynthetic process GO:0009058; cofactor binding GO:0048037; ACP phosphopantetheine attachment site binding involved in fatty acid biosynthetic process GO:0000036; phosphopantetheine binding GO:0031177	
2,6	MGG_08297	NADH:flavin oxidoreductase/NADH oxidase	catalytic activity GO:0003824; oxidoreductase activity GO:0016491; oxidation-reduction process GO:0055114; FMN binding GO:0010181	
2,6	MGG_09376	FAD binding domain-containing protein; SignalP - noTM	catalytic activity GO:0003824; oxidoreductase activity GO:0016491; oxidation-reduction process GO:0055114; flavin adenine dinucleotide binding GO:0050660; UDP-N-acetylmuramate dehydrogenase activity GO:0008762; oxidoreductase activity, acting on CH-OH group of donors GO:0016614	
2,5	MGG_03823	NADH oxidase	catalytic activity GO:0003824; oxidoreductase activity GO:0016491; oxidation-reduction process GO:0055114; FMN binding GO:0010181; mycelium development GO:0043581	
2,5	MGG_16812	FAD linked oxidase	catalytic activity GO:0003824; oxidoreductase activity GO:0016491; oxidation-reduction process GO:0055114; flavin adenine dinucleotide binding GO:0050660; UDP-N-acetylmuramate dehydrogenase activity GO:0008762; oxidoreductase activity, acting on CH-OH group of donors GO:0016614	
2,4	MGG_09333	Nucleoside-diphosphate-sugar epimerase; SignalP - noTM	nucleotide binding GO:0000166	
2,4	MGG_03662	Phosphoadenosine phosphosulfate reductase	catalytic activity GO:0003824; metabolic process GO:0008152; oxidation-reduction process GO:0055114; phosphoadenylyl-sulfate reductase (thioredoxin) activity GO:0004604; cysteine biosynthetic process GO:0019344; sulfate assimilation, phosphoadenylyl sulfate reduction by phosphoadenylyl-sulfate reductase (thioredoxin) GO:0019379; nucleus GO:0005634; cytosol GO:0005829	

2,4	MGG_09007	Alcohol dehydrogenase; GroES-like chaperone domain	nucleotide binding GO:0000166; oxidoreductase activity GO:0016491; oxidation-reduction process GO:0055114; zinc ion binding GO:0008270; endoplasmic reticulum GO:0005783; Golgi apparatus GO:0005794; cytoplasm GO:0005737	
2,5	MGG_00156	Nmr1; lack of Nmr1 has no effect in disease symptoms production; targeted deletion of *NMR1* partially restores Δtps1 pathogenicity defects	nucleotide binding GO:0000166; negative regulation of nitrogen compound metabolic process GO:0051172	[11]
2,4	MGG_05059	SDH1; scytalone dehydratase, enzyme involved in melanin synthesis	melanin metabolic process GO:0006582; scytalone dehydratase activity GO:0030411	[12]
2,4	MGG_08943	ATPase	ATP binding GO:0005524	
2,3	MGG_05940	Short-chain dehydrogenase/reductase SDR	nucleotide binding GO:0000166; metabolic process GO:0008152; oxidoreductase activity GO:0016491; oxidation-reduction process GO:0055114	
2,3	MGG_09189	Cellobiose dehydrogenase; SignalP - noTM	oxidation-reduction process GO:0055114; flavin adenine dinucleotide binding GO:0050660; oxidoreductase activity, acting on CH-OH group of donors GO:0016614	
2,3	MGG_08656	multidrug resistance MdtG protein domain; TM	integral component of membrane GO:0016021; plasma membrane GO:0005886; transmembrane transport GO:0055085; transporter activity GO:0005215	
2,3	MGG_07216	Versicolorin reductase; homologue of Buf1, involved in melanin synthesis	nucleotide binding GO:0000166; metabolic process GO:0008152; oxidoreductase activity GO:0016491; oxidation-reduction process GO:0055114	
2,3	MGG_12983	Short-chain dehydrogenase/reductase SDR	nucleotide binding GO:0000166; metabolic process GO:0008152; oxidoreductase activity GO:0016491; oxidation-reduction process GO:0055114	
2,3	MGG_04736	NAD(P)-binding domain	nucleotide binding GO:0000166; mycelium development GO:0043581	
2,2	MGG_12228	Alcohol dehydrogenase	nucleotide binding GO:0000166; oxidoreductase activity GO:0016491; oxidation-reduction process GO:0055114; zinc ion binding GO:0008270	
2,2	MGG_05331	Protein-S-isoprenylcysteine O-methyltransferase	transferase activity GO:0016740; integral component of membrane GO:0016021; methyltransferase activity GO:0008168; methylation GO:0032259; protein C-terminal S-isoprenylcysteine carboxyl O-methyltransferase activity GO:0004671; C-terminal protein methylation GO:0006481; cellular protein localization GO:0034613; endoplasmic reticulum GO:0005783	
2,2	MGG_08989	Short-chain dehydrogenase/reductase SDR	nucleotide binding GO:0000166; metabolic process GO:0008152; oxidoreductase activity GO:0016491; oxidation-reduction process GO:0055114	
2,2	MGG_13464	Laccase; SignalP - noTM	metal ion binding GO:0046872; oxidoreductase activity GO:0016491; oxidation-reduction process GO:0055114	
2,2	MGG_17812	Alcohol dehydrogenase; GroES-like chaperone domain; renamed from MGG_09328	nucleotide binding GO:0000166; oxidoreductase activity GO:0016491; oxidation-reduction process GO:0055114; zinc ion binding GO:0008270; mycelium development GO:0043581	
2,1	MGG_17542	Putative uncharacterized protein	oxidoreductase activity GO:0016491; oxidation-reduction process GO:0055114	
2,1	MGG_02095	NmrA-like domain		
2,1	MGG_10961	FAD linked oxidase; SignalP - noTM	catalytic activity GO:0003824; oxidoreductase activity GO:0016491; oxidation-reduction process GO:0055114; flavin adenine dinucleotide binding GO:0050660; UDP-N-acetylmuramate dehydrogenase activity GO:0008762; oxidoreductase activity, acting on CH-OH group of donors GO:0016614	
2,1	MGG_01368	Short-chain dehydrogenase/reductase SDR	nucleotide binding GO:0000166; metabolic process GO:0008152; oxidoreductase activity GO:0016491; oxidation-reduction process GO:0055114	
2,1	MGG_10316	MFS (major facilitator superfamily) domain; TM	integral component of membrane GO:0016021; transmembrane transport GO:0055085	
2,0	MGG_11468	NmrA-like domain	nucleotide binding GO:0000166	

2,0	MGG_06740	Purine transporter, homology with *A. nidulans* AzgA (e-162)	Membrane GO:0016020; Transport GO:0006810; transmembrane transport GO:0055085; transporter activity GO:0005215	

**Secreted/cell wall-related (100 down-regulated; 40 up-regulated)**				
-41,8	MGG_08355	uncharacterised protein; SignalP-noTM		
-21,5	MGG_08944	uncharacterized protein; SignalP-noTM		
-19,1	MGG_05805	uncharacterised protein; SignalP-noTM		
-17,3	MGG_07356	uncharacterised protein; SignalP-noTM		
-13,9	MGG_09019	secreted phospholipase A2, SignalP-noTM; paralogue of MGG_01367, also down in *Δtpc1*	A deletion mutant on one phospholipase A2 (MGG_14014, MoPplA) in *M. oryzae* exhibits retarded growth and conidial germination, reduced conidiation, appressorial turgor pressure and pathogenicity to rice CO-39 [13]	
-13,8	MGG_09321	uncharacterised protein; SignalP-noTM		
-12,1	MGG_05406	uncharacterised protein; SignalP-TM		
-10,9	MGG_01941	FAD binding domain; FAD linked oxidase; SignalP-noTM. The UDP-N-acetylmuramate dehydrogenase participates in aminosugars metabolism. One of the most common aminosugars is N-Acetyl-D-glucosamine, the main component of chitin.	catalytic activity GO:0003824; oxidoreductase activity GO:0016491; oxidation-reduction process GO:0055114; flavin adenine dinucleotide binding GO:0050660; UDP-N-acetylmuramate dehydrogenase activity GO:0008762; oxidoreductase activity, acting on CH-OH group of donors GO:0016614	
-8,3	MGG_12068	uncharacterised protein; SignalP-noTM		
-8,0	MGG_07630	uncharacterised protein; SignalP-noTM		
-8,0	MGG_10394	uncharacterised protein; SignalP-noTM		
-7,9	MGG_02987	carboxylesterase, para-nitrobenzyl esterase; SignalP-noTM; paralogue of MGG_12798 and MGG_00593, also down in *Δtpc1*		
-7,2	MGG_03593	uncharacterised protein; SignalP-noTM	mycelium development GO:0043581	
-6,7	MGG_07246	uncharacterised protein; SignalP-noTM	interaction with host via protein secreted by type II secretion system GO:0052051	
-6,2	MGG_00703	DUF3129, SignalP-noTM; paralogue of Mas3/Gas1	mycelium development GO:0043581	[14]
-5,5	MGG_08501	Fringe-like domain (a beta-1,3-N-acetylglucosaminyltransferase enzyme); SignalP-noTM	Membrane GO:0016020; transferase activity, transferring glycosyl groups GO:0016757	
-5,4	MGG_07868	Glycosyl hydrolase family 10; SignalP-noTM. Closely related to MGG_14243; xylanase GH10; specifically expressed in the compatible interaction; PHI-base[5] number:2209	catalytic activity GO:0003824; metabolic process GO:0008152; hydrolase activity GO:0016787; hydrolase activity, hydrolyzing O-glycosyl compounds GO:0004553; carbohydrate metabolic process GO:0005975; hydrolase activity, acting on glycosyl bonds GO:0016798; cation binding GO:0043169; endo-1,4-beta-xylanase activity GO:0031176; polysaccharide catabolic process GO:0000272	[7,15]
-5,4	MGG_09252	uncharacterised protein; SignalP-noTM		
-5,2	MGG_03806	Calcium-binding EF-hand-like domain; SignalP-noTM	calcium ion binding GO:0005509	
-5,1	MGG_15353	ECM (extracellular matrix)-Hyaluronan synthase domain; nucleotide diphospho sugar transferase domain: domain with a Rossmann like fold and can be found in diverse glycosyltransferases		
-5,0	MGG_07623	Cbp2; chitin-binding protein; SignalP-noTM; not expressed in *Δcon7* [16]	chitin-binding GO:0008061	
-4,9	MGG_04547	GH61; SignalP-noTM. Endoglucanase II		
-4,8	MGG_02884	MoFLP1; FAS1 cell adhesion domain; SignalP-noTM		[17]

-4,7	MGG_12798	Carboxylesterase, type B/Lipase GDXG; SignalP-noTM; paralogue of MGG_02987 and MGG_00593 also down in *Δtpc1*	metabolic process GO:0008152; hydrolase activity GO:0016787	
-4,7	MGG_03436	ECM (extracellular matrix)-Cell wall galactomannoprotein; highly up-regulated in appressorium[18]; mannosylated protein		
-4,9	MGG_07625	uncharacterised protein; SignalP-noTM		
-4,5	MGG_04258	uncharacterised protein; SignalP-noTM		
-4,5	MGG_08373	uncharacterised protein; SignalP-noTM		
-4,4	MGG_09875	DUF3129; CAS1 domain; SignalP-noTM; paralogue of Mas3/Gas1		[14]
-4,4	MGG_13993	uncharacterised protein; SignalP-noTM		
-4,4	MGG_13971	Glycosyltransferase AER61; Catalyzes the transfer of a single N-acetylglucosamine from UDP-GlcNAc to a serine or threonine residue in extracellular proteins resulting in their modification with a beta-linked N-acetylglucosamine (O-GlcNAc)	transferase activity, transferring glycosyl groups GO:0016757	
-4,2	MGG_07631	GH61; Fungal cellulose binding domain-containing protein; signalP-noTM		
-4,2	MGG_00283	uncharacterised protein; SignalP-noTM		
-4,2	MGG_09817	Serine-type endopeptidase; SignalP-noTM	hydrolase activity GO:0016787; proteolysis GO:0006508; peptidase activity GO:0008233; serine-type peptidase activity GO:0008236; serine-type endopeptidase activity GO:0004252	
-4,0	MGG_02295	NAD(P)-binding domain; SignalP-noTM	nucleotide binding GO:0000166	
-4,0	MGG_15751	uncharacterised protein; SignalP-TM		
-4,0	MGG_09998	uncharacterised protein; SignalP-TM	interaction with host via protein secreted by type II secretion system GO:0052051	
-4,0	MGG_08830	uncharacterised protein; SignalP-noTM		
-4,0	MGG_04757	uncharacterised protein; SignalP-noTM		
-3,8	MGG_09352	Minor extracellular protease vpr; serine-type endopeptidase activity Peptidase S8/S53; DUF1034; SignalP-noTM	Membrane GO:0016020; hydrolase activity GO:0016787; proteolysis GO:0006508; peptidase activity GO:0008233; serine-type peptidase activity GO:0008236; serine-type endopeptidase activity GO:0004252; cell wall GO:0005618	
-3,7	MGG_05785	Bas113; GH32 Glycosyl hydrolases family 32; SignalP-noTM	metabolic process GO:0008152; hydrolase activity GO:0016787; hydrolase activity, hydrolyzing O-glycosyl compounds GO:0004553; carbohydrate metabolic process GO:0005975; hydrolase activity, acting on glycosyl bonds GO:0016798	[19]
-3,6	MGG_16585	uncharacterised protein; SignalP-TM		
-3,6	MGG_08497	uncharacterised protein; SignalP-TM		
-3,6	MGG_07969	similar to Bas2(e-29); SignalP-noTM		[20]
-3,6	**MGG_04732**	GH18; chitinase-Glycoside hydrolase, family 18; SignalP-noTM	catalytic activity GO:0003824; metabolic process GO:0008152; hydrolase activity GO:0016787; hydrolase activity, hydrolyzing O-glycosyl compounds GO:0004553; carbohydrate metabolic process GO:0005975; hydrolase activity, acting on glycosyl bonds GO:0016798; cation binding GO:0043169; chitinase activity GO:0004568; chitin catabolic process GO:0006032	this study
-3,4	MGG_00593	Carboxylesterase, type B; SignalP-noTM; paralogue of MGG_02987 and MGG_12798	hydrolase activity GO:0016787	
-3,3	**MGG_09956**	SignalP-TM; similar to cell fusion protein Ham6 (*N. crassa;* e-79)[21], and NoxD/Pro41[22,23]		this study

-3,1	MGG_07311	uncharacterised protein; SignalP-noTM		
-3,1	MGG_00282	endopeptidase; subtilisin-related; DUF1034; no predicted SignalP-no TM	Membrane GO:0016020; proteolysis GO:0006508; serine-type endopeptidase activity GO:0004252; cell wall GO:0005618	
-3,0	MGG_02531	Minor extracellular protease vpr; peptidase, subtilisin-related; SignalP-noTM; this gene is down regulated in Mosom1 mutant[24]; gene knockout shows no detectable changes in conidial germination and appressorium formation but reduced disease symptoms	hydrolase activity GO:0016787; proteolysis GO:0006508; peptidase activity GO:0008233; serine-type peptidase activity GO:0008236; serine-type endopeptidase activity GO:0004252	[25]
-3,0	MGG_05608	uncharacterised protein; SignalP-noTM		
-2,9	MGG_07624	uncharacterized protein; SignalP - noTM		
-2,9	MGG_02309	carboxypeptidase S1; similar to CPY (e-34), a broad-specificity C-terminal exopeptidase involved in non-specific protein degradation in the vacuole; SignalP-noTM		
-2,9	MGG_06234	uncharacterized protein; SignalP - noTM	interaction with host via protein secreted by type II secretion system; GO:0052051	
-2,9	MGG_03439	acid phosphatase; SurE-like nucleotidase; SignalP - noTM	hydrolase activity GO:0016787	
-2,9	MGG_12337	DUF3129; SignalP-noTM; paralogue of Mas3/Gas1		[14]
-2,9	MGG_03995	carboxypeptidase S1'; similar to yeast CPY (e-40), a broad-specificity C-terminal exopeptidase; SignalP-noTM	proteolysisnGO:0006508; carboxypeptidase activity GO:0004180; serine-type carboxypeptidase activity GO:0004185	
-2,9	MGG_00992	uncharacterized protein; SignalP-noTM		
-2,8	MGG_04603	uncharacterized protein; SignalP - noTM		
-2,8	MGG_00245	uncharacterized protein; SignalP-noTM	viral capsid GO:0019028	
-2,8	MGG_09818	peptidase; SignalP - TM	Proteolysis GO:0006508; aspartic-type endopeptidase activity GO:0004190	
-2,8	MGG_10330	glutamate carboxypeptidase 2; SignalP - noTM	Proteolysis GO:0006508; peptidase activity GO:0008233; carboxypeptidase activity GO:0004180; mycelium development GO:0043581	
-2,7	MGG_08415	Serin endopeptidase; SignalP - noTM	Membrane GO:0016020; Proteolysis GO:0006508; serine-type endopeptidase activity GO:0004252; cell wall GO:0005618; proteolysis GO:0006508	
-2,7	MGG_01532	uncharacterized protein; SignalP-noTM		
-2,7	MGG_16033	zinc metalloprotease; previously MGG_02107; fibronectin type3 and M28 peptidase domain; SignalP - noTM		
-2,6	MGG_02339	Carboxypeptidase S1; SignalP - noTM	Proteolysis GO:0006508; carboxypeptidase activity GO:0004180; serine-type carboxypeptidase activity GO:0004185	
-2,6	MGG_09641	CW_beta-glucan_synthesis; SignalP - noTM	mycelium development GO:0043581	
-2,6	MGG_00659	Glucan 1,3-beta-glucosidase; pectate-lyase fold; SignalP - TM		
-2,6	MGG_05092	uncharacterized protein; SignalP - noTM		
-2,5	MGG_05539	Carboxypeptidase 2; SignalP-noTM	Proteolysis GO:0006508; zinc ion binding GO:0008270; carboxypeptidase activity GO:0004180; metallocarboxypeptidase activity GO:0004181	
-2,5	MGG_09468	AB_hydrolase; SignalP - noTM		
-2,5	MGG_08480	Alpha/beta hydrolase; SignalP - noTM		
-2,5	MGG_02234	uncharacterized protein; SignalP - noTM	interaction with host via protein secreted by type II secretion system GO:0052051	

-2,4	MGG_01149	Extracellular CFEM domain; SignalP-noTM		
-2,4	MGG_07646	Alpha-glucuronidase; SignalP - noTM	catalytic activity GO:0003824; extracellular region GO:0005576; carbohydrate metabolic process GO:0005975; cation binding GO:0043169; xylan catabolic process GO:0045493; alpha-glucuronidase activity GO:0046559;	
-2,4	MGG_06224	uncharacterized protein; SignalP - noTM		
-2,3	MGG_09844	uncharacterized protein; SignalP - TM		
-2,3	MGG_08748	peptidase M6-like; SignalP - noTM	Proteolysis GO:0006508; peptidase activity GO:0008233	
-2,2	MGG_17532	DUF1349; SignalP - no TM; previously MGG_00114		
-2,2	MGG_09314	Lipolytic enzyme; esterase; SignalP - TM	metabolic process GO:0008152; hydrolase activity GO:0016787; hydrolase activity, hydrolyzing O-glycosyl compounds GO:0004553; carbohydrate metabolic process GO:0005975; hydrolase activity, acting on glycosyl bonds GO:0016798	
-2,2	MGG_01863	Aminopeptidase Y; SignalP-noTM	Membrane GO:0016020; calcium ion binding GO:0005509; proteolysis GO:0006508; peptidase activity GO:0008233; aminopeptidase activity GO:0004177; mannosyl-oligosaccharide 1,2-alpha-mannosidase activity GO:0004571	
-2,2	MGG_02756	uncharacterized protein; SignalP - noTM		
-2,2	MGG_08971	uncharacterized protein; SignalP - noTM		
-2,2	MGG_04078	DUF1996; SignalP - noTM		
-2,2	MGG_01692	Alpha carbonic anhydrase; SignalP-noTM		
-2,1	MGG_02368	galactose oxidase; Kelch and DUF1929 domains; SignalP - no TM	mycelium development GO:0043581	
-2,1	MGG_09428	uncharacterized protein; SignalP - TM		
-2,1	MGG_10318	uncharacterized protein; SignalP - noTM		
-2,1	MGG_02582	galactose oxidase/kelch, beta-propeller; SignalP - 1TM		
-2,1	MGG_07748	uncharacterized protein; SignalP - noTM		
-2,1	MGG_06303	AB-hydrolase; SignalP - TM	metabolic process GO:0008152; hydrolase activity GO:0016787; endoplasmic reticulum GO:0005783; cytoplasm GO:0005737	
-2,1	MGG_09639	Alpha-1,3-glucan synthase AgsB (*A. nidulans* ; e0.0); SignalP - TM	catalytic activity GO:0003824; carbohydrate metabolic process GO:0005975; biosynthetic process GO:0009058; cation binding GO:0043169	
-2,1	MGG_03671	uncharacterized protein; SignalP - noTM		
-2,1	MGG_01001	SignalP-noTM; GH81; similar to *A. nidulans* engA (e0.0), a 1,3-beta-glucosidase with a role in carbon starvation-induced autolytic cell wall degradation[26]	cell wall macromolecule catabolic process GO:0016998; endo-1,3(4)-beta-glucanase activity GO:0033903	
-2,1	MGG_01367	Secreted phospholipase A2; SignalP- 1 TM ; paralogue of MGG_09019 that is also down in Δtpc1		
-2,1	MGG_10440	Endoribonuclease L-PSP; SignalP - noTM	cellular nitrogen compound biosynthetic process GO:0044271; deaminase activity GO:0019239	
-2,0	MGG_09272	Beta-glucosidase 1; SignalP - noTM	metabolic process GO:0008152; hydrolase activity GO:0016787; hydrolase activity, hydrolyzing O-glycosyl compounds GO:0004553; carbohydrate metabolic process GO:0005975; hydrolase activity, acting on glycosyl bonds GO:0016798	

-2,0	MGG_16250	AB hydrolase; SignalP - noTM	mycelium development GO:0043581	
-2,0	MGG_12712	uncharacterized protein; SignalP - noTM		
-2,0	MGG_12468	uncharacterized protein; SignalP - TM	mycelium development GO:0043581	
9,1	MGG_08253	uncharacterised protein; SignalP-noTM		
8,7	MGG_14057	lipase; AB hydrolase; SignalP - noTM	lipid catabolic process GO:0016042; triglyceride lipase activity GO:0004806	
7,5	MGG_00311	Peptidase G1/Concanavalin A-like lectin/glucanase; SignalP-noTM; regulated by PdeH; mutant has no virulence defects	Proteolysis GO:0006508; aspartic-type endopeptidase activity GO:0004190; interaction with host via protein secreted by type II secretion system GO:0052051	[27]
6,6	MGG_14053	uncharacterised protein; SignalP-noTM		
4,2	MGG_03685	Avr-Pi54 allele; SignalP-noTM		[28]
4,0	MGG_06647	Peptidase A1; SignalP-noTM	Proteolysis GO:0006508; aspartic-type endopeptidase activity GO:0004190	
3,8	MGG_02139	uncharacterised protein; SignalP-noTM		
3,7	MGG_07558	uncharacterised protein; SignalP-noTM		
3,5	MGG_09106	uncharacterised protein; SignalP-noTM		
3,5	MGG_01944	uncharacterised protein; SignalP-noTM		
3,5	MGG_07643	uncharacterised protein; SignalP-noTM		
3,4	MGG_08045	uncharacterized protein; SignalP-noTM		
3,3	MGG_12582	uncharacterized protein; SignalP-noTM		
3,2	MGG_07644	uncharacterized protein; SignalP-noTM		
3,2	MGG_09849	uncharacterized protein; SignalP-noTM		
3,0	MGG_06771	Chitin-binding domain; SignalP-no TM	chitin binding GO:0008061	
2,9	MGG_07571	LysM domain-containing protein; mutant shows no defects; SignalP-noTM	cell wall macromolecule catabolic process GO:0016998	[27]
2,7	MGG_18035	uncharacterized protein; SignalP - TM		
2,7	MGG_03495	uncharacterized protein; SignalP - noTM		
2,6	MGG_15902	uncharacterized protein; SignalP - TM	mycelium development GO:0043581	
2,6	MGG_15427	uncharacterized protein; SignalP - noTM		
2,6	MGG_08774	Chitin deacetylase; SignalP - no TM	catalytic activity GO:0003824; carbohydrate metabolic process GO:0005975; hydrolase activity, acting on carbon-nitrogen (but not peptide) bonds GO:0016810	
2,5	MGG_08451	uncharacterized protein; SignalP - noTM		
2,5	MGG_09569	uncharacterized protein; SignalP - noTM		
2,5	MGG_02754	Carbohydrate-binding WSC domain; SignalP - noTM		
2,5	MGG_05871	Pth11; CFEM protein; G-protein couple receptor; required for appressorium formation and virulence; SignalP - TM; regulated by Tra1 transcription factor[29]	cellular nitrogen compound biosynthetic process GO:0044271	[30]
2,4	MGG_12551	uncharacterized protein; SignalP - TM		

2,4	MGG_17824	uncharacterized protein; SignalP - noTM		
2,3	MGG_07411	uncharacterized protein; SignalP - noTM		
2,3	MGG_02989	uncharacterized protein; SignalP - noTM		
2,3	MGG_14712	uncharacterized protein; SignalP - noTM; reannotated from MGG_00209		
2,3	MGG_01255	Cellobiose dehydrogenase, extracellular flavocytochrome that degrades both cellulose and lignin; SignalP - TM	catalytic activity GO:0003824; carbohydrate binding GO:0030246; carbohydrate catabolic process GO:0016052	
2,3	MGG_05982	uncharacterized protein; SignalP - noTM	cellular nitrogen compound biosynthetic process GO:0044271	
2,2	MGG_10467	uncharacterized protein; SignalP - noTM		
2,2	MGG_03347	uncharacterized protein; SignalP - noTM		
2,2	MGG_10001	uncharacterized protein; SignalP - noTM		
2,2	MGG_00815	uncharacterized protein; SignalP - noTM		
2,1	MGG_07766	uncharacterized protein; SignalP - noTM		
2,1	MGG_02097	uncharacterized protein; SignalP - noTM		
2,0	MGG_01764	Polysaccharide lyase; SignalP - noTM		
**Others (19 down-regulated; 35 up-regulated)**				
-6,8	MGG_08360	Serine hydrolase FSH		
-5,4	MGG_07615	Methyltransferase	methyltransferase activity GO:0008168; methylation GO:0032259	
-5,1	MGG_00099	HAD-superfamily hydrolase; Cut1 (*N. crassa* ; e0.0)	metabolic process GO:0008152; hydrolase activity GO:0016787; mycelium development GO:0043581	
-4,6	MGG_14657	S-adenosyl-L-methionine-dependent methyltransferase-like		
-3,3	MGG_02942	Ribonuclease Inhibitor (CATH Superfamily 3.80.10.10)		
-3,3	MGG_06751	Uncharacterised protein family UPF0761; TM	ribonuclease activity GO:0004540	
-3,3	**MGG_02246**	Conidiation-specific protein 6; Con6 (*N. crassa*)	Nucleus GO:0005634; cytosol GO:0005829	this study; [31]
-2,9	MGG_05855	peptidase; 1 TM	peptidase activity GO:0008233	
-2,7	MGG_13765	peptidase S28	Proteolysis GO:0006508; serine-type peptidase activity GO:0008236	
-2,7	MGG_10103	Het domain (heterokaryon incompatibility)		
-2,4	MGG_05505	Vacuole effluxer Atg22 like domain; 1 TM ; autophagy protein		
-2,4	MGG_10657	acyl transferase; TM	transferase activity, transferring acyl groups other than amino-acyl groups GO:0016747	
-2,3	MGG_09681	6-blade_b-propeller_TolB-like		
-2,2	MGG_08019	F-box domain-containing protein	mycelium development GO:0043581	
-2,1	MGG_04582	Glycoside hydrolase; similar to yeast SCW11; T-DNA mutant shows reduced virulence	catalytic activity GO:0003824; carbohydrate metabolic process GO:0005975; cation binding GO:0043169; mycelium development GO:0043581; carbohydrate metabolic process GO:0005975; pathogenesis GO:0009405; spore germination GO:0009847; cytokinesis, completion of separation GO:0007109; fungal-type cell wall GO:0009277	[4]

-2,1	MGG_08592	RNA-binding protein; survival motor neuron (SMN) domain involved in the assembly of spliceosomal small nuclear ribonucleoproteins (snRNPs)	Nucleus GO:0005634; mRNA processing GO:0006397; cytoplasm GO:0005737; RNA binding GO:0003723	
-2,1	MGG_16393	aminoacyl-tRNA hydrolase	aminoacyl-tRNA hydrolase activity GO:0004045; mitochondrial translation GO:0032543	
-2,1	MGG_08034	Rhodanese-like protein		
-2,0	MGG_11237	dsRNA-binding-like domain		
12,9	MGG_03212	uncharacterised protein; orthologue of *N. crassa* annotated as a methyltransferase (metabolism of beta-lactams)	mycelium development GO:0043581	
12,1	MGG_05719	Heat shock protein Hsp20/HSP20-like chaperone	response to stress GO:0006950	
7,2	MGG_09227	uncharacterised protein; TM ; *N. crassa* orthologue NCU03679 localises in developing septa[31]		
6,3	MGG_06790	Clr5 domain; This domain is found at the N terminus of the Clr5 protein, which is involved in silencing in fission yeast.		
4,2	MGG_15049	OTT_1508-like nucleic acid/nucleotide deaminase		
4,2	MGG_03329	HSP20-like chaperone; mutant defective in pathogenicity, appressorium formation, and conidial morphology	response to stress GO:0006950; mycelium development GO:0043581	[4]
4,1	MGG_09600	Heterokaryon incompatibility region	lipid catabolic process GO:0016042; triglyceride lipase activity GO:0004806	
3,3	MGG_11445	Clr5 domain; This domain is found at the N terminus of the Clr5 protein, which is involved in silencing in fission yeast.		
3,2	MGG_07250	DEAD/DEAH box helicase; promote translational arrest, polysome disassembly and decapping; Mtr4 (e-19) , Ski2 (e-19)	ATP binding GO:0005524; hydrolase activity GO:0016787; nucleic acid binding GO:0003676; helicase activity GO:0004386; ATP-dependent helicase activity GO:0008026; cytosol GO:0005829	
3,1	MGG_11084	chromosomal proteins modulating gene activities and/or chromatin structure - SET-domain; SET6 (e-6) SET domain protein of unknown function; deletion heterozygote is sensitive to compounds that target ergosterol biosynthesis	Binding GO:0005488	
2,7	MGG_04358	HSP20-like chaperone		
2,7	MGG_02648	dynamin GTPase involved in membrane transport between compartments through the scission of a wide range of vesicles and organelles. The dynamin family are also microtubule-associated force-producing proteins which are involved in the production of microtubule bundles.	nucleotide binding GO:0000166; GTPase activity GO:0003924; GTP binding GO:0005525; obsolete GTP catabolic process GO:0006184	
2,7	MGG_06739	SAM-dependent methyl transferase domain		
2,6	MGG_07560	peptidase A1	Proteolysis GO:0006508; aspartic-type endopeptidase activity GO:0004190	
2,5	MGG_00768	cyclin-like F-box domain		
2,5	MGG_00018	Integral membrane protein; Major Facilitator Superfamily	transmembrane transport GO:0055085; integral component of membrane GO:0016021; mycelium development GO:0043581	
2,5	MGG_00836	NUDIX hydrolase domain	hydrolase activity GO:0016787	
2,5	MGG_17265	Metallothionein domain	metal ion binding GO:0046872	
2,4	MGG_01294	PAZ and PIWI domains found in Argonaute protein family; DUF1785	nucleic acid binding GO:0003676	
2,4	MGG_08113	Marvel (MAL and related proteins for vesicle trafficking and membrane link) domain; TM	mycelium development GO:0043581; membrane GO:0016020	

2,4	MGG_17222	glutathione S-transferase; participates in the detoxification of reactive electrophillic compounds by catalysing their conjugation to glutathione; reannotated form MGG_10108		
2,4	MGG_07159	Stc1 domain; in *S. pombe* , proteins containing this domain act as protein linkers, which link the chromatin modifying CLRC complex to RNAi by tethering it to the RITS complex.		
2,3	MGG_07227	Cys-rich domain; TM		
2,3	MGG_07594	methyltransferase type 11	metabolic process GO:0008152; methyltransferase activity GO:0008168; methylation GO:0032259	
2,2	MGG_09400	NUDIX hydrolase domain		
2,1	MGG_00675	cyclin-like F-box domain		
2,1	MGG_09384	Chloramphenicol acetyltransferase-like domain		
2,1	MGG_05825	Transferase	transferase activity GO:0016740; transferase activity, transferring acyl groups other than amino-acyl groups GO:0016747	
2,1	MGG_08052	glycosyl transferase; TM	transferase activity, transferring glycosyl groups GO:0016757	
2,0	MGG_03639	Arginine-tRNA ligase	nucleotide binding GO:0000166; ATP binding GO:0005524; Cytoplasm GO:0005737; arginine-tRNA ligase activity GO:0004814; arginyl-tRNA aminoacylation GO:0006420	
2,0	MGG_02400	pirin family, highly conserved nuclear proteins that may function as transcriptional regulators with a role in apoptosis; TM		
2,0	MGG_12911	tRNA 2'-phosphotransferase 1	transferase activity GO:0016740; transferase activity, transferring phosphorus-containing groups GO:0016772; tRNA splicing, via endonucleolytic cleavage and ligation GO:0006388	
2,0	MGG_01796	Anaphase-promoting complex, subunit Cdc26	anaphase-promoting complex GO:0005680; regulation of mitotic metaphase/anaphase transition GO:0030071; anaphase-promoting complex-dependent proteasomal ubiquitin-dependent protein catabolic process GO:0031145	
2,0	MGG_02114	dynamin GTPase involved in membrane transport between compartments through the scission of a wide range of vesicles and organelles. The dynamin family are also microtubule-associated force-producing proteins which are involved in the production of microtubule bundles.	nucleotide binding GO:0000166; GTPase activity GO:0003924; GTP binding GO:0005525; obsolete GTP catabolic process GO:0006184	
2,0	MGG_02065	Kinesin light chain, variant	catalytic activity GO:0003824; binding GO:0005488; nucleoside metabolic process GO:0009116; ADP binding GO:0043531	

**unknown (27 down-regulated; 39 up-regulated)**				
-8,5	MGG_10245	DUF3328; TM		
-5,3	MGG_15150	uncharacterised protein		
-5,0	MGG_03403	uncharacterised protein		
-4,9	MGG_03383	uncharacterised protein		
-4,4	MGG_15129	uncharacterised protein		
-4,1	MGG_08207	DUF3176; TM		

-3,7	MGG_11809	DUF3405; TM		
-3,6	MGG_14523	uncharacterised protein		
-3,3	MGG_11255	uncharacterised protein		
-3,2	MGG_06081	uncharacterised protein		
-3,0	MGG_16612	uncharacterized protein		
-2,8	MGG_10006	uncharacterized protein		
-2,6	MGG_13716	uncharacterized protein; TM		
-2,6	MGG_03504	DUF3129		
-2,6	MGG_04120	uncharacterized protein		
-2,6	MGG_00947	uncharacterized protein		
-2,5	MGG_05747	uncharacterized protein		
-2,5	MGG_00513	uncharacterized protein		
-2,5	MGG_01986	DUF1996; TM		
-2,3	MGG_04339	uncharacterized protein; 7 TM		
-2,3	MGG_09571	uncharacterized protein		
-2,3	MGG_09469	WD40 repeat domain		
-2,2	MGG_01940	uncharacterized protein; TM		
-2,1	MGG_08936	uncharacterized protein		
-2,1	MGG_16497	uncharacterized protein		
-2,1	MGG_10257	uncharacterized protein; TM		
-2,0	MGG_04181	uncharacterized protein		
6,2	MGG_15337	Zygote-specific protein; TM		
5,8	MGG_09612	uncharacterized protein; TM ; fumarase folding)		
3,9	MGG_17073	uncharacterized protein	mycelium development GO:0043581	
3,7	MGG_02956	uncharacterized protein		
3,7	MGG_18055	uncharacterized protein		
3,6	MGG_02206	uncharacterized protein		
3,5	MGG_01177	uncharacterized protein; TM	integral component of membrane GO:0016021	
3,3	MGG_16965	uncharacterized protein; TM		
3,2	MGG_03629	uncharacterized protein		
3,0	MGG_14358	uncharacterized protein	zinc ion binding GO:0008270; intracellular GO:0005622	
2,9	MGG_05131	uncharacterized protein		
2,9	MGG_03864	uncharacterized protein; TM		

2,8	MGG_16099	uncharacterized protein	nucleotide binding GO:0000166	
2,7	MGG_11684	uncharacterized protein; TM		
2,7	MGG_16028	uncharacterized protein		
2,7	MGG_00798	uncharacterized protein		
2,6	MGG_10921	uncharacterized protein		
2,6	MGG_17107	uncharacterized protein		
2,6	MGG_09604	uncharacterized protein; TM		
2,5	MGG_04595	uncharacterized protein		
2,5	MGG_05106	uncharacterized protein; TM		
2,4	MGG_00071	uncharacterized protein		
2,4	MGG_02208	uncharacterized protein; TM		
2,3	MGG_00287	uncharacterized protein		
2,3	MGG_13772	uncharacterized protein		
2,3	MGG_10788	uncharacterized protein		
2,3	MGG_03279	uncharacterized protein		
2,2	MGG_10480	uncharacterized protein; TM		
2,2	MGG_08916	uncharacterized protein		
2,2	MGG_09752	uncharacterized protein		
2,2	MGG_03358	uncharacterized protein; TM		
2,2	MGG_08026	uncharacterized protein; TM		
2,1	MGG_05143	uncharacterized protein		
2,1	MGG_02105	uncharacterized protein		
2,1	MGG_09412	uncharacterized protein		
2,1	MGG_17400	uncharacterized protein; previously MGG_11531		
2,0	MGG_00433	uncharacterized protein		
2,0	MGG_04910	uncharacterized protein;		
2,0	MGG_04729	uncharacterized protein		

REFERENCES (S3 Table)

1. Lu J, Cao H, Zhang L, Huang P, Lin F (2014) Systematic analysis of Zn2Cys6 transcription factors required for development and pathogenicity by high-throughput gene knockout in the rice blast fungus. PLoS Pathog 10: e1004432.
2. Kim S, Singh P, Park J, Park S, Friedman A, et al. (2011) Genetic and molecular characterization of a blue light photoreceptor MGWC-1 in *Magnaporthe oryzae* . Fungal Genetics and Biology 48: 400-407.
3. Park SY, Choi J, Lim SE, Lee GW, Park J, et al. (2013) Global Expression Profiling of Transcription Factor Genes Provides New Insights into Pathogenicity and Stress Responses in the Rice Blast Fungus. PLoS Pathogens 9: e1003350.
4. Jeon J, Park SY, Chi MH, Choi J, Park J, et al. (2007) Genome-wide functional analysis of pathogenicity genes in the rice blast fungus. Nature Genetics 39: 561-565.
5. Villalba F, Collemare J, Landraud P, Lambou K, Brozek V, et al. (2008) Improved gene targeting in *Magnaporthe grisea* by inactivation of MgKU80 required for non-homologous end joining. Fungal Genetics and Biology 45: 68-75.
6. Andersen MR, Nielsen JB, Klitgaard A, Petersen LM, Zachariasen M, et al. (2013) Accurate prediction of secondary metabolite gene clusters in filamentous fungi. Proceedings of the National Academy of Sciences of the United States of America 110: E99-E107.
7. Nguyen QB, Itoh K, Van Vu B, Tosa Y, Nakayashiki H (2011) Simultaneous silencing of endo-beta-1,4 xylanase genes reveals their roles in the virulence of *Magnaporthe oryzae* . Molecular Microbiology 81: 1008-1019.

8. Battaglia E, Klaubauf S, Vallet J, Ribot C, Lebrun MH, et al. (2013) Xlr1 is involved in the transcriptional control of the pentose catabolic pathway, but not hemi-cellulolytic enzymes in *Magnaporthe oryzae* . Fungal Genetics and Biology 57: 76-84.
9. Klaubauf S, Ribot C, Melayah D, Lagorce A, Lebrun MH, et al. (2013) The pentose catabolic pathway of the rice blast fungus *Magnaporthe oryzae* involves a novel pentose reductase restricted to few fungal species. FEBS Lett 587: 1346-1352.
10. Ding SL, Liu WD, Iliuk A, Ribot C, Vallet J, et al. (2010) The Tig1 Histone Deacetylase Complex Regulates Infectious Growth in the Rice Blast Fungus *Magnaporthe oryzae* . Plant Cell 22: 2495-2508.
11. Wilson RA, Gibson RP, Quispe CF, Littlechild JA, Talbot NJ (2010) An NADPH-dependent genetic switch regulates plant infection by the rice blast fungus. Proceedings of the National Academy of Sciences of the United States of America 107: 21902-21907.
12. Chumley FG, Valent B (1990) Genetic analysis of melanin deficient, nonpathogenic mutants of *Magnaporthe grisea* . Molecular Plant-Microbe Interactions 3: 135-143.
13. Liu XH, Zhuang FL, Lu JP, Lin FC (2011) Identification and molecular cloning MoplaA gene, a homologue of Homo sapiens PLAA, in *Magnaporthe oryzae* . Microbiological Research 167: 8-13.
14. Xue C, Park G, Choi W, Zheng L, Dean RA, et al. (2002) Two Novel Fungal Virulence Genes Specifically Expressed in Appressoria of the Rice Blast Fungus. Plant Cell 14: 2107-2119.
15. Kim SG, Wang Y, Lee KH, Park Z-Y, Park J, et al. (2013) In-depth insight into in vivo apoplastic secretome of rice-*Magnaporthe oryzae* interaction. Journal of proteomics 78: 58-71.
16. Odenbach D, Breth B, Thines E, Weber RW, Anke H, et al. (2007) The transcription factor Con7p is a central regulator of infection-related morphogenesis in the rice blast fungus *Magnaporthe grisea* . Molecular Microbiology 64: 293-307.
17. Liu TB, Chen GQ, Min H, Lin FC (2009) MoFLP1, encoding a novel fungal fasciclin-like protein, is involved in conidiation and pathogenicity in *Magnaporthe oryzae* . Journal of Zhejiang University-Science B 10: 434-444.
18. Oh Y, Donofrio N, Pan HQ, Coughlan S, Brown DE, et al. (2008) Transcriptome analysis reveals new insight into appressorium formation and function in the rice blast fungus *Magnaporthe oryzae* . Genome Biology 9: R85.
19. Giraldo MC, Dagdas YF, Gupta YK, Mentlak TA, Yi M, et al. (2013) Two distinct secretion systems facilitate tissue invasion by the rice blast fungus *Magnaporthe oryzae* . Nature Communications 4: 1996.
20. Mosquera G, Giraldo MC, Khang CH, Coughlan S, Valent B (2009) Interaction Transcriptome Analysis Identifies *Magnaporthe oryzae* BAS1-4 as Biotrophy-Associated Secreted Proteins in Rice Blast Disease. Plant Cell 21: 1273-1290.
21. Fu C, Ao J, Dettmann A, Seiler S, Free SJ (2014) Characterization of the *Neurospora crassa* Cell Fusion Proteins, HAM-6, HAM-7, HAM-8, HAM-9, HAM-10, AMPH-1 and WHI-2. Plos One 9: e107773.
22. Lacaze I, Lalucque H, Siegmund U, Silar P, Brun S (2014) Identification of NoxD/Pro41 as the homologue of the p22(phox) NADPH oxidase subunit in fungi. Molecular Microbiology 95: 1006-1024.
23. Siegmund U, Marschall R, Tudzynski P (2015) BcNoxD, a putative ER protein, is a new component of the NADPH oxidase complex in *Botrytis cinerea* . Molecular Microbiology 95: 988-1005.
24. Yan X, Li Y, Yue X, Wang C, Que Y, et al. (2011) Two Novel Transcriptional Regulators Are Essential for Infection-related Morphogenesis and Pathogenicity of the Rice Blast Fungus *Magnaporthe oryzae* . PLoS Pathog 7: e1002385.
25. Guo M, Chen Y, Du Y, Dong YH, Guo W, et al. (2011) The bZIP Transcription Factor MoAP1 Mediates the Oxidative Stress Response and Is Critical for Pathogenicity of the Rice Blast Fungus *Magnaporthe oryzae* . PLoS Pathogens 7: e1001302.
26. Szilagyi M, Kwon NJ, Dorogi C, Pocsi I, Yu JH, et al. (2010) The extracellular beta-1,3-endoglucanase EngA is involved in autolysis of *Aspergillus nidulans* . Journal of Applied Microbiology 109: 1498-1508.
27. Zhang HF, Liu KY, Zhang X, Tang W, Wang JS, et al. (2011) Two Phosphodiesterase Genes, PDEL and PDEH, Regulate Development and Pathogenicity by Modulating Intracellular Cyclic AMP Levels in *Magnaporthe oryzae* . Plos One 6: e17241.

28. Devanna NB, Vijayan J, Sharma TR (2014) The Blast Resistance Gene Pi54 of Cloned from *Oryza officinalis* Interacts with Avr-Pi54 through Its Novel Non-LRR Domains. Plos One 9.
29. Breth B, Odenbach D, Yemelin A, Schlinck N, Schroder M, et al. (2013) The role of the Tra1p transcription factor of *Magnaporthe oryzae* in spore adhesion and pathogenic development. Fungal Genetics and Biology 57: 11-22.
30. DeZwaan TM, Carroll AM, Valent B, Sweigard JA (1999) *Magnaporthe grisea* Pth11p Is a Novel Plasma Membrane Protein That Mediates Appressorium Differentiation in Response to Inductive Substrate Cues. Plant Cell 11: 2013-2030.
31. White BT, Yanofsky C (1993) Structural Characterization and Expression Analysis of the *Neurospora* Conidiation Gene Con-6. Developmental Biology 160: 254-264.
32. Lai J, Ng SK, Liu FF, Patkar RN, Lu Y, et al. (2010) Marker fusion tagging, a new method for production of chromosomally encoded fusion proteins. Eukaryot Cell 9: 827-830.
