# Supplementary material for: Tpc1 is an important Zn(II)2Cys6 transcriptional regulator required for polarized growth and virulence in the rice blast fungus
Source: PLoS Pathog. 2017 Jul 24;13(7):e1006516. doi: 10.1371/journal.ppat.1006516 (PMC5542705; doi:10.1371/journal.ppat.1006516)
Supplement: S4 Table — (PDF) [file ppat.1006516.s012.pdf]

S4 Table. List of primers used in this study.

**General primers**	
M13F	5’ CGCCAGGGTTTTCCCAGTCACGAC 3’
M13R	5’ AGCGGATAACAATTTCACACAGGA 3’
HY	5’ GGATGCCTCCGCTCGAAGTA 3’
YG	5’ CGTTGCAAGACCTGCCTGAA 3’
**PCR-amplified hygromycin probe for Southern**	
M13F	5’ CGCCAGGGTTTTCCCAGTCACGAC 3’
M13R	5’ AGCGGATAACAATTTCACACAGGA 3’
**PCR-amplified probes for Southerns**	
B1RN-MGG_14045	GGGGACTGCTTTTTTGTACAAACTTGTTTGGAAACCTTTGGGTGAA
PEBP2-F-probe	ATTACGGACACAATGCCGAC
5' -PEBP1-Probe	GCGCACATCATGCAAATCTTGG
3' PEBP1-Probe	TTAACCCATCAAGACCGATGC
TPC1-Fw-probe	AGAACACGGCCATCTTCAAC
TPC1-Rv-probe	ACAGGCAGCTTCTCCTTGAC
B4-TPC1	5' GGGGACAACTTTGTATAGAAAAGTTGCTGTGCATCCTGAAGCCTGGAAAC 3'
B1RNTPC1KO	5' GGGGACTGCTTTTTTGTACAAACTTGCATGTTGCTGAAAGTCGGCTCC 3'
B4-MGG_09956	GGGGACAACTTTGTATAGAAAAGTTGACTCGCTCGCCTCGCTTGAT
B1RN-MGG_09956	GGGGACTGCTTTTTTGTACAAACTTGTGTGTCGTTTTTTTTTTCGTTGTT
B4-MGG_02246	GGGGACAACTTTGTATAGAAAAGTTGCACAGGTTCTCACCGATGCC
B1RN-MGG_02246	GGGGACTGCTTTTTTGTACAAACTTGATTGATGGTTGGGGGTGGTT
B4-MGG_04732	GGGGACAACTTTGTATAGAAAAGTTGGCTTCAGCATTCACCCTGTA
B1RN-MGG_04732	GGGGACTGCTTTTTTGTACAAACTTGGTTTGCTGGAGTCCTTAATT
**Primers for qPCR analysis**	
PRO41-q-F	CCTGATGTTAGCGTGGGAGT
PRO41-q-R	TAGTAGACGGCAGCGTTGGT
PEBP2-q-F	ATGGTGGACCCAGACCTGAC
PEBP2-q-R	CCTGGTTGATGCTGACGTTG
PEBP1-q-F	GCCAGAGCACTGACAACCAA
PEBP1-q-R	ATGTAGGGCGCCAAGGTATG
CON6-q-F	CTCGAGGAGCAGTTTGATGG
3'-CON6-Probe	GCTCCTTGGCAGACTCCTTG
qTPC1 For	TTTCCAACACACGCCCATTG
qTPC1 Rev	TCCTGCCAATTTGCAATGCC
GH18-q-F	GCTACTTTGCCGGCTACCAC
GH18-q-R	GTCTCGGCAAACGCGTACTT
qMst12F	5' CCCGACCTCCGAGGGTGGAT 3'
qMst12R	5' CATGCCACCGGCATTGATGG 3'
qTPC1 For	5' TTTCCAACACACGCCCATTG 3'
qTPC1 Rev	5' TCCTGCCAATTTGCAATGCC 3'
PRO41-q-F	5' CCTGATGTTAGCGTGGGAGT 3'
PRO41-q-R	5' TAGTAGACGGCAGCGTTGGT 3'
**Primers for PCR mutants analysis**	
ext-5'-CON6	CATCCCGTGGCAGGTCGC
ext-3'-CON6	CGCAAGTCTTGGCACCAGTCC
5'-rev-PEBP1	GCCGTTTACGTCAAAGCTG
ext-5'-PEBP1	GAGATGCAGAGGAAAGTGGC
ext-5'-PEBP2	GTAGCGATCAAAATCCGAGG
ext-3'-PEBP2	CGCTTGGACGACGACCCG
ext-5'-PRO41	GACGGCTGTCATGCGACTAG,
ext-3'-PRO41	GGCTGGGCTTCGTTCAGGATG
CT74-1	CACGCCATGTAGTGTATTGACC
AndyF_T3	AATTAACCCTCACTAAAGGGA
5-HYGR	GCCGATAGTGGAAACCGACGC
HSVtk-Probe	CCGAGCCGATGACTTACTGG
SU	5' GCTCCTCGATCAGAGTCTGAGGC 3'
UR	5' ACAGTCGGTTACCCTGGCGGTGC 3'
ext-5'-GH18	GGTAAAGCCTCCCCTCTCCCTC
CT74-3	GTAGAATAGGTAAGTCAGATTG
**Primers for analysis of T-DNA insertion in M1422**	
AT-RB	5' GATTGTCGTTTCCCGCCTTCAG 3'

AT-LB2	5' CCAGTACTAAAATCCAGATCCC 3'	
**Primers for generating delection mutants**		
B4-TPC1	5' GGGGACAACTTTGTATAGAAAAGTTGCTGTGCATCCTGAAGCCTGGAAAC 3'	
B1RNTPC1KO	5' GGGGACTGCTTTTTTGTACAAACTTGCATGTTGCTGAAAGTCGGCTCC 3'	
B2RCRTPC1KO	5' GGGGACAGCTTTCTTGTACAAAGTGGCCTTGCCTACAACCTGACGCTTC 3'	
B3-TPC1	5' GGGGACAACTTTGTATAATAAAGTTGAGCAGGTGTGAGGCGAGGTAGC 3'	
B4-MGG_09956	5' GGGGACAACTTTGTATAGAAAAGTTGACTCGCTCGCCTCGCTTGAT 3'	
B1RN-MGG_09956	5' GGGGACTGCTTTTTTGTACAAACTTGTGTGTCGTTTTTTTTTTCGTTGTT 3'	
B2RC-MGG_09956	5' GGGGACAGCTTTCTTGTACAAAGTGGGCTATTGCTTCGCGCCCGCC 3'	
B3-MGG_09956	5' GGGGACAACTTTGTATAATAAAGTTGGTTCTGCAAATGCTTCTTGAT 3'	
B4-MGG_02246	5' GGGGACAACTTTGTATAGAAAAGTTGCACAGGTTCTCACCGATGCC 3'	
B1RN-MGG_02246	5' GGGGACTGCTTTTTTGTACAAACTTGATTGATGGTTGGGGGTGGTT 3'	
B2RC-MGG_02246	5' GGGGACAGCTTTCTTGTACAAAGTGGAAGCAGACTACATTAGTCGC 3'	
B3-MGG_02246	5' GGGGACAACTTTGTATAATAAAGTTGCTCCCTACCTGCCTACCCTT 3'	
B4-MGG_06800	5' GGGGACAACTTTGTATAGAAAAGTTGATCCTGAAGACGAGGACGAG 3'	
B1RN-MGG_06800	5' GGGGACTGCTTTTTTGTACAAACTTGGGCTAGTCTAGTCTAGTTTG 3'	
B2RC-MGG_06800	5' GGGGACAGCTTTCTTGTACAAAGTGGGGTTATTTCGGGGCCACTCT 3'	
B3-MGG_06800	5' GGGGACAACTTTGTATAATAAAGTTGATCATTAGCCGCCATTCGCC 3'	
B4-MGG_14045	5' GGGGACAACTTTGTATAGAAAAGTTGGAAGAGCACTACAATAGACA 3'	
B1RN-MGG_14045	5' GGGGACTGCTTTTTTGTACAAACTTGTTTGGAAACCTTTGGGTGAA 3'	
B2RC-MGG_14045	5' GGGGACAGCTTTCTTGTACAAAGTGGTTTGTGAGTGATTATGGGTC 3'	
B3-MGG_14045	5' GGGGACAACTTTGTATAATAAAGTTGTGGAGGACTGCGCGACAGAA 3'	
B4-MGG_04732	GGGGACAACTTTGTATAGAAAAGTTGGCTTCAGCATTCACCCTGTA	
B1RN-MGG_04732	GGGGACTGCTTTTTTGTACAAACTTGGTTTGCTGGAGTCCTTAATT	
B3_MGG_04732:	GGGGACAACTTTGTATAATAAAGTTGTGTGAGCGATTAACGAAATG	
B2RC-MGG_04732:	GGGGACAGCTTTCTTGTACAAAGTGGGCTTGGAAATGTAGAGGTT	
**Primers for generation of GFP protein fusion constructs**		
7.GFP_F	5’ ATGGTGAGCAAGGGCGAGGAGCTG 3’	
TPC1_GFP_F	5’ CCTGCCAACAACCAATCCAC 3’	
TPC1_GFP_Fnest	5’ GTCGTGAACTTGATTGTGGGC 3’	
TPC1_GFP_R	5‘ CAGCTCCTCGCCCTTGCTCACCATATTGGCACGAATGACCAACTTG 3’	
GFP_4658_R	5’ GTCCATGCCGTGAGTGATCC 3’	
B1RC-TPC1	5' GGGGACTGCTTTTTTGTACAAACTTGGATTGGCACGAATGACCAACTTGG 3'	
B4-TPC1	5' GGGGACAACTTTGTATAGAAAAGTTGCTGTGCATCCTGAAGCCTGGAAAC 3'	
B2RC-TPC1	5' GGGGACAGCTTTCTTGTACAAAGTGGGACACTTGTCCGATTATCAGTGTC 3'	
B3-TPC1	5' GGGGACAACTTTGTATAATAAAGTTGAGCAGGTGTGAGGCGAGGTAGC 3'	
**Primers for generation of mRFP protein fusion constructs**		
B4-MGG_09956	5' GGGGACAACTTTGTATAGAAAAGTTGCAACCCTGCGTATCCGACGTTCTG 3'	
B1RN-MGG_09956	5' GGGGACTGCTTTTTTGTACAAACTTGTGACACGCGCACCACGCCC 3'	
B2RC-MGG_09956	5' GGGGACAGCTTTCTTGTACAAAGTGGGCTATTGCTTCGCGCCCGCC 3'	
B3-MGG_09956	5' GGGGACAACTTTGTATAATAAAGTTGCCCGTTCGTCACCCCGTCTATGC 3'	
B1-PRO41	5' GGGGACAAGTTTGTACAAAAAAGCAGGCTAACGAAAAAAAAAACGACACA 3'	
B2	5' GGGGACCACTTTGTACAAGAAAGCTGGGT 3'	
**primers for generation of Y2H constructs and NoxD:GFP**		
NoxD_EcoRI	5' GGAGGCCAGT**GAATTC**ATGGGAAGACTTATCAAGAACCAC 3'	
NoxD_SmaI	5' CGATGCCC A**CCCGGG**TTAGACACGCGCACCACGCCCTCC 3'	
Nox1_EcoRI	5' CATGGAGGCC**GAATTC**ATGTCGGTCGGAGAGTTCTTGGCT 3'	
Nox1_BamHI	5' GCAGGTCGAC**GGATCC**CTAGAAATGCTCCTTCCAGAAGCG 3'	
Nox2_EcoRI	5' CATGGAGGCC**GAATTC**ATGTCTGGATACGGCTACGGAGGA 3'	
Nox2_BamHI	5' GCAGGTCGAC**GGATCC**CTAGAAATTCTCCTTGCCCCATAC 3'	
NoxR_EcoRI	5' CATGGAGGCC**GAATTC**ATGTCGCTCAAGCAGGAGATAGAA 3'	
NoxR_BamHI	5' GCAGGTCG[illegible]	7
NoxD_GFPF	5' TAGAACTAGT**GGATC**TATCGAATGAACTGGTTGTTCGAG 3'	
NoxD_GFPR	5' GACACGCGCACCACGCCCTCCACG 3'	
GFPF	5' CGTGGTGCGCGTGTCATGGTGAGCAAGGGCGAGGAGCTG 3'	
TrpCR	5' **CGGTATCGATAAGCT**GTGGAGATGTGGAGTGGGCGCTTA 3'	
**primers used for ChIP analysis**		
qNoxD-F0	5' CGAGTCGGCGGTTGCTTGAT3' 3'	
qNoxD-R0	5' GAGCACCCGCGTGGTAATGG 3'	
qNoxD-F1	5' AGTCGCACTCCCCTCTTGTTTTC 3'	

qNoxD-R1	5' AGTACTATTGCGCAGGGCAAACA 3'
qNoxD-F2	5' TGTTTGCCCTGCGCAATAGTACT 3'
qNoxD-R2	5' TGCAAGACGACGTAACAGACGAG 3'
qNoxD-F3	5' TCGTCTGTTACGTCGTCTTGCAC 3'
qNoxD-R3	5' CCCCGTCGATATGAAAGAGTGCT 3'
qNoxD-F4	5' CCTGCACCTCATCCAATCTTTAAC 3'
qNoxD-R4	5' GCGCGGGTGAGAAAAAGAGACTA 3'
qNoxD-CDS-F	5' CCTGATGTTAGCGTGGGAGT 3'
qNoxD-CDS-R	5' TAGTAGACGGCAGCGTTGGT 3'
qNoxD-F5	5' CCGGGCTGGGTTGTTTCAGA 3'
qNoxD-R5	5' TCGGGAGGGTTTCGTTTGAATG 3'
**primers used for EMSA**	
Fwd EcoRI-TPC1	5' CGTG gaattc ATGACTTACGACTACCGAG 3'
Rev NotI-TPC1	5' CGTG gcggccgc ATTGGCACGAATGACCAAC 3'
Fwd BamHI-MST12	5' CGTG ggatcc ATGTATTCCCATCCACACAAC 3'
Rev NotI-MST12	5' CGTG gcggccgc CATCATGCCACCGGCATTG 3'
Fwd KpnI NoxD-Y probe1	5' CGTG ggtacc GTGTGTGTACCCCACAAG 3'
Rev KpnI NoxD-Z probe1	5' CGTG ggtacc GACGACGTAACAGACGAG 3'
Fwd KpnI NoxD-Z probe2	5' CGTG ggtacc CGCTTGCTCCTGGAC 3'
Rev KpnI NoxD-W probe2	5' CGTG ggtacc GTCGAAGTGAAGGTAGAATAG 3'
Rev KpnI NoxD probe3	5' CGTG ggtacc TGTGTCGTTTTTTTTTTCGTTG 3'
Fwd KpnI NoxD 516 probe3	5' CGTG ggtacc CCTGCACCTCATCCAATC 3'
Rev KpnI YdiU	5' CGTG ggtacc CTATGCAGATATGTCCTTCC 3'
Fwd KpnI YdiU 499	5' CGTG ggtacc GGAACAATAGTTATTGTAACTG 3'
